# Comprehensive analysis of SET domain gene family in foxtail millet identifies the putative role of *SiSET14* in abiotic stress tolerance

**DOI:** 10.1038/srep32621

**Published:** 2016-09-02

**Authors:** Chandra Bhan Yadav, Mehanathan Muthamilarasan, Anand Dangi, Shweta Shweta, Manoj Prasad

**Affiliations:** 1 National Institute of Plant Genome Research, Aruna Asaf Ali Marg, New Delhi – 110067, India

## Abstract

SET domain-containing genes catalyse histone lysine methylation, which alters chromatin structure and regulates the transcription of genes that are involved in various developmental and physiological processes. The present study identified 53 SET domain-containing genes in C_4_ panicoid model, foxtail millet (*Setaria italica*) and the genes were physically mapped onto nine chromosomes. Phylogenetic and structural analyses classified SiSET proteins into five classes (I–V). RNA-seq derived expression profiling showed that *SiSET* genes were differentially expressed in four tissues namely, leaf, root, stem and spica. Expression analyses using qRT-PCR was performed for 21 *SiSET* genes under different abiotic stress and hormonal treatments, which showed differential expression of these genes during late phase of stress and hormonal treatments. Significant upregulation of *SiSET* gene was observed during cold stress, which has been confirmed by over-expressing a candidate gene, *SiSET14* in yeast. Interestingly, hypermethylation was observed in gene body of highly differentially expressed genes, whereas methylation event was completely absent in their transcription start sites. This suggested the occurrence of demethylation events during various abiotic stresses, which enhance the gene expression. Altogether, the present study would serve as a base for further functional characterization of *SiSET* genes towards understanding their molecular roles in conferring stress tolerance.

Histone methylation mediated by SET domain-containing protein family plays a pivotal role in epigenetic regulation of gene expression^1^. These proteins share a highly conserved SET domain, and are initially identified in members of *Su*(*var*)*3-9*, *Enhancer of zeste (E*(*z*)) and *Trithorax (trx*) genes in *Drosophila melanogaster*^2^. SET domain-containing proteins are involved in methylation of lysine (K) residues in histone H3 (K4, K9, K27, and K36) and H4 (K20)^2^; however, H3K79 is an exception^3^. Each lysine can carry one, two or three methyl residue(s), known as mono-, di- and tri-methylation, respectively. In general, di-/tri- methylation of H3K4 and H3K36 may lead to transcriptional inactivation, whereas di-methylation of H3K9 and tri-methylation of H3K27 could promote gene silencing in plants and animals^4^. Noteworthy, proteins comprising SET domain have also been reported in viruses and prokaryotes^5^,^6^. The SET domain is approximately 130 amino acids in length, and contains two non-contiguous regions made by N- and C-terminal of the primary sequence, named SET-N and SET-C, respectively, and an insert region (SET-I)^7^.

SET domain protein methyltransferases potential players in regulation of chromatin structure and function^8^,^9^. These proteins catalyze the transfer of methyl groups from the co-factor S-adenosylmethionine (AdoMet) to ε-amino group of particular lysine residues of protein substrates including the N-terminal tails of histone (H3 or H4) and the large subunit of the Rubisco holoenzyme complex^10^. In plants, a number of SET genes have been identified and functionally characterized for their roles in growth and development, and stress response. In *Arabidopsis*, SET domain proteins are categorized into seven different classes based on their action on specific lysine residues. These include, (i) Enhancer of zeste [E(Z)] homologs (H3K27); (ii) ASH1 homologs (H3K36); (iii) trithorax homologs and related proteins (H3K4); (iv) proteins with a SET and PHD domain; (v) suppressor of variegation [Su(var)] homologs and relatives (H3K9); (vi) proteins with an interrupted SET domain; and (vii) RBCMT and other SET related proteins^11^.

In Arabidopsis, three E(Z) homologs were reported, which are named as CURLY LEAF (CLF), MEDEA (MEA), and SWINGER (SWN). The former plays role in regulating flowering time, and leaf and flower morphology by repressing the floral homeotic genes AGAMOUS (AG) and SHOOTMERISTEMLESS (STM)^12^,^13^. MEA maintains their imprinting in course of endosperm development, and methylation of H3K27 is linked to MEA silencing during vegetative development and male gametogenesis^14^,^15^. SWN shows partial functional redundancy with the other two homologs^16^,^17^,^18^. Among the monocots, SET domain-containing proteins have been characterized in rice and maize. In rice, *OsCLF* and *OsEZ1*, both of which are E(Z) homologs, are expressed preferentially in young seedlings and during reproductive development^19^. SDG714 and SDG728 encoding H3K9me2 histone methyltransferase show specific functions in chromatin modification and retrotransposon repression^20^. However, no such study has been performed in the C_4_ biofuel model, foxtail millet (*Setaria italica*). Foxtail millet is a C_4_ panicoid millet crop extensively cultivated in arid and semi-arid regions of the world, and it is well known for stress tolerance, better nitrogen use and water use efficiency^21^. Compared to other millet crops, extensive functional characterization of several gene families have been performed in foxtail millet to delineate their role in abiotic stress tolerance^21^; however, characterization of SET domain-containing gene family remains unstudied. In view of this, the present study has been undertaken to identify and analyze the expression of SET domain-containing gene family. In addition, overexpression of candidate gene in yeast and the methylation analysis have been performed.

## Results and Discussion

### SET domain-containing gene family in foxtail millet

SET domain-containing proteins that catalyses histone lysine methylation are key player in chromatin regulation which is essential to regulate genes in a variety of developmental and physiological processes. These proteins modify chromatin structure and thereby regulate eukaryotic gene transcription. Lysine methylation of histones plays a crucial role in various biological processes ranging from regulation of transcription to formation of heterochromatin^4^. Histone methylation in plants regulates a range of physiological processes such as gametogenesis, embryogenesis, seed development, flowering time, branching and floral identity^22^,^23^,^24^. In recent years, increasing evidences indicate that SET domain-containing proteins are encoded by large multigene family in plants and investigation of the SET domain gene family will facilitate understanding the epigenetic mechanism of gene regulation.

In this present study, 53 SET domain containing genes were identified in foxtail millet, and all the corresponding proteins comprised of three major conserved domains (PreSET, SET and PostSET). The identified genes were named *SiSET01* to *SiSET53* according to their position on chromosomes (Supplementary Table 1). Arabidopsis and rice genomes encode 49 and 34 SET domain-containing proteins, respectively^11^,^23^. In *Vitis vinifera*, 33 genes belonging to this family of chromatin remodelling factors were identified by Aquea *et al.*^25^. This number is also similar to that found in other organisms including zebrafish, human, and fruit fly which have 58, 47 and 29 SET domain-containing proteins, respectively^26^.

All the SiSET proteins contained SET domain which is a major player in methylation of lysine (K) residues in various histones, specifically K4, K9, K27, and K36 in histone H3 and K20 in histone H4. Multiple sequence alignment of SET domain of all SiSET proteins showed that 13 amino acids were highly conserved and a number of proteins have higher similarity (above (92%) with sorghum and maize (Supplementary Fig. 1). Besides SET domain, other important domains such as AWS, WIYLD, Pre-SET, SAD_SRA, Rubis-subs-bind, PWWP, FYRC, FYRN, PostSET, PHD, SAND, GYF, ZnF-C2H2, ZnF-MYND and TPR were identified to be present in SiSET proteins (Supplementary Table 2). Based on specific domains and their role in histone methylation, SiSET domain gene family was categorized into five classes (class I to V), following Pontvianne *et al.*^23^ in Arabidopsis and Lu *et al.*^27^ in rice. Interestingly, the classification identified a single gene in *S. italica* which belongs to class I EZ1 like proteins, whereas in Arabidopsis E(z) proteins, MEA (MEDEA), CLF (CURLY LEAF) and SWN (SWINGER) belong class I proteins. These proteins function similar to *Drosophila* and mammalian E(z) proteins in catalysing H3K27me3, which is associated with gene-repression^28^.

Class II proteins namely, SiSET02, SiSET03, *SiSET14*, SiSET19, SiSET23, SiSET24 and SiSET44 have a specific domain, ASH1 (ABSENT OF SMALL HOMEOTIC DISCS 1) which methylates both H3K4 and H3K36, and their specific role has been characterized in Arabidopsis. TRX (TRITHORAX) proteins (SiSET12, SiSET16: ATX2, SiSET28: ATX4 and SiSET27: ATX5) belong to class III. SiSET01, SiSET17, SiSET20, SiSET33, SiSET36, SiSET46 and SiSET49 to SiSET53 (ATX1-RELATED 5 and 6) are class IV which has peculiar function to add single methyl group to H3K27. Class V genes namely, SiSET18: SUVR4, SiSET22: SUVH4, SiSET26: SUVH1, SiSET29: SUVH4, SiSET30: SUVR3, SiSET32: SUVH4, SiSET39: SUVH5, SiSET41: SUVH9, SiSET42: SUVR4, SiSET44: SUVH6, SiSET47: SUVH1, SiSET48: SUVH1, (SU(VAR)3-9 members) are responsible for H3K9 methylation, which is related to heterochromatin formation. Similar studies were performed by Qian *et al.*^29^ in maize, where 43 ZmSET genes were identified. Similarly, 43 OsSET genes was identified in rice^27^, 49 genes in *Brassica rapa*^30^, 59 and 47 SET genes in poplar and Arabidopsis, respectively^23^,^31^.

The length of SiSET proteins were found to be highly variable ranging from 301 amino acids (SiSET51) to 2267 amino acids (SiSET36). Similarly, the pI and molecular weight were also ranged from 4.44 to 9.57 and 10613.3 to 255545.2 kDa, respectively. Protein instability index showed that all the SiSET proteins were unstable except SiSET21 (38.38), SiSET22 (35.41), SSET37 (36.29), SiSET45 (339.73) and SiSET53 (39.27) (Supplementary Table 1).

### Gene structure, chromosomal position and duplication

Considerable variations in gene length (ranging from 296 bp to 14268 bp) was observed in *SiSET* genes. The largest gene was 14 kb in length in *S. italica*, whereas 14.3 kb in *O. sativa*, 34.1 kb in *S. bicolor*, 44.5 kb in *Z. mays*, 16.0 kb in *B. distachyon*, 11.1 kb in *A. thaliana*, 29.7 kb in *G. max*, 22.1 kb in *S. lycopersicum*, 18.1 kb in *P. trichocarpa*, and 50.8 kb in *S. moellendorffii*. Minimum gene length ranges from 0.16 kb to 2.2 kb in all the ten plant species (Table 1). Maximum of 26 exons were observed in almost all the *SiSET* genes (Supplementary Fig. 2), while maximum exon numbers ranged from 24 to 26 in the reported plant species. Average number of exons per gene was also higher in *S. italica*, *P. trichocarpa* and *S. moellendorffii* (12.76, 12.44 and 16.77, respectively). Noteworthy, exon-intron ratio was maximum (2.7) in *S. moellendorffii*, whereas minimum in *G. max* (0.41), while it was 0.84 and 0.83 in *S. italica* and *O. sativa*, respectively.

**Figure 1:**
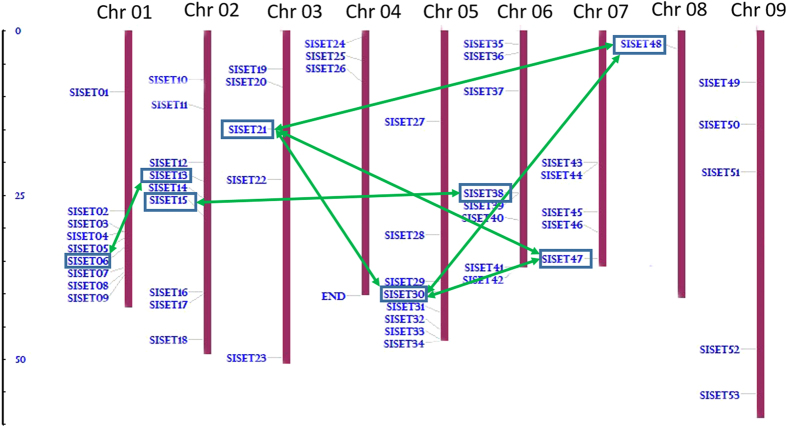
Physical map of foxtail millet showing the chromosomal location of *SiSET* genes. *SiSET* genes were individually mapped on their corresponding physical location on nine chromosomes. The vertical bars represent chromosomes and the scale at the left indicates the size (in Mbp).

Physical map was constructed by plotting the *SiSET* domain-containing genes on nine chromosomes of foxtail millet (Fig. 1), which showed that 53 *SiSET* genes were distributed on the all 9 chromosomes. Chromosome 1 and 2 comprised a maximum of 9 *SiSET* genes (~52%), and a minimum of one gene was present in chromosome 8. Eight *SiSET* genes were found to be located on chromosome 5 and 6, where most of the genes were present at distal part of the chromosome. Considering the whole genome duplication events that have occurred during course of evolution, a comprehensive gene duplication analysis was also performed and we observed that five *SiSET* genes namely, *SiSET06, SiSET13, SiSET21, SiSET30, SiSET38* and *SiSET47* were segmentally duplicated (Fig. 1). Similar collinear duplication events have also been reported in Arabidopsis for *SDG7/24, SDG27/30*, and *SDG16/29* gene-pairs^32^. In maize, *Sdg125/Sdg126* gene-pairs were reported to be duplicated from the ancient allopolyploid origin of this species^33^.

### Phylogenetic analysis of SiSET proteins

**Figure 2:**
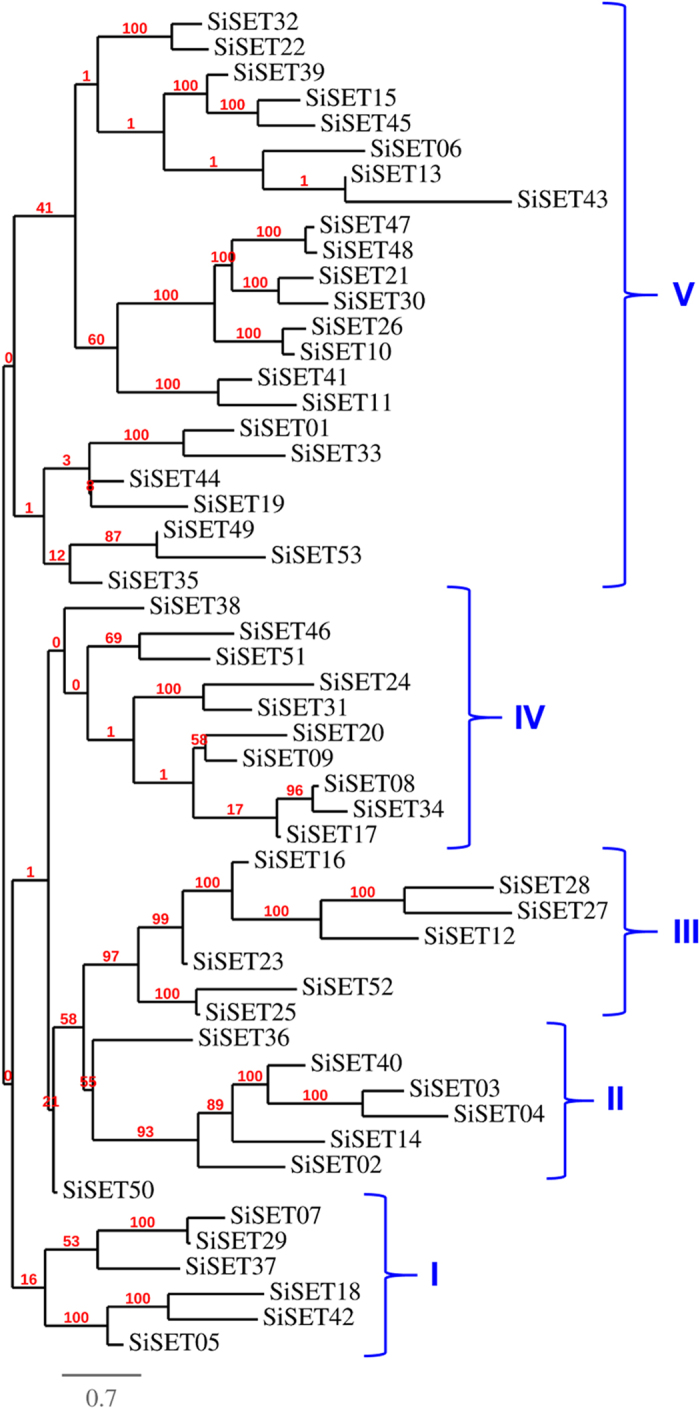
Phylogenetic relationship among SiSET proteins. The identified full-length amino acid sequences of SiSET proteins were used for construction of phylogenetic tree with RAxML v8.0.0 using maximum likelihood method.

Phylogenetic analysis grouped SiSET proteins into five classes (class I to V) (Fig. 2). Class I and III comprised of 6 and 7 proteins, respectively, which contained EZ1 and ATX domains. Class III and IV comprised of 7 and 10 proteins, respectively, and a maximum of 23 SiSET proteins belong to Class V. Similarly, phylogenetic tree was constructed with SET proteins from foxtail millet, Arabidopsis, Brachypodium, maize and rice, which showed clustering of class-specific proteins demonstrating the evolutionary conservedness among these proteins (Supplementary Fig. 3). Further, MEME motif search tool was employed to identify the conserved motifs of SET domains from SiSET proteins. Six distinct motifs were identified and found to be located in SiSET proteins (Supplementary Fig. 4). Maximum of 48 out of 53 (92.3%) SET proteins have motif 1, 40 (76.9%) have motif 2 and 50 (96.1%) SiSET proteins have motif 3. Approximately, 40–50% of SiSET proteins have motif 4, 5 and 6 (Supplementary Fig. 4). All SET proteins were searched for homology signal for their presence in plant and animal kingdom. Phylogenetic tree constructed with all species showed that all the SiSETs displays strong intense signal based on their conservation. Seven *SiSET* genes were well conserved among dicot, monocot and fungi. Fifty percent of SiSET were conserved in algae and bryophytes. *SiSET04, SiSET14, SiSET30, SiSET39* and *SiSET43* showed similarity with *Plasmodium,* oomycetes, fungus, members of Euglenozoa and Amoebozoa (Supplementary Fig. 5).

### Gene ontology annotation of *SiSET* gene family

Gene ontology analysis was performed to identify various biological processes and molecular functions in which for SiSET proteins have roles (Supplementary Fig. 6). As the genes of this family contain SET domain which is known to induce lysine methylation modifications, most of the *SiSET* genes were predicted to possess catalytic activity, with a biological role in gene silencing, chromatin binding, zinc ion binding activity in gene regulation. The molecular functions are mostly restricted to methyl transferase and other catalytic activities (Supplementary Fig. 6). SiSET proteins were found to participate in diverse biological activities such as histone lysine methylation, virus-induced gene silencing, production of miRNAs involved in gene silencing by miRNA, production of ta-siRNAs involved in RNA interference, regulation of DNA replication, cytokinesis by cell plate formation, regulation of transcription, DNA-dependent regulation of G2/M transition of mitotic cell cycle, DNA endoreduplication, pollen development, anther dehiscence, histone H3-K27 methylation, carpel development, vegetative to reproductive phase transition of meristem and stamen development (Supplementary Fig. 6). Similar GO annotations were reported in case of rice *SET* genes by Lu *et al.*^27^. In order to delineate the precise biological functions and molecular processes of *SiSET* genes, co-expression networks were constructed (Supplementary Fig. 7). Biological process categorized cellular and metabolic processes of these SiSET proteins, which showed that predominant of these proteins were involved in chromatin modification, histone methylation and histone modification. Further, the co-expression network also showed the molecular processes of these proteins, which are observed to possess histone binding and methyltransferase activities. These corroborated well with the biological processes predicted using co-expression network (Supplementary Fig. 7). Similarly, in rice, the co-expression network analysis provided clues on the involvement of OsSET proteins in regulation of cell cycle by histone modification and reproductive development^27^.

### Identification of orthologous genes and their evolutionary relationships

**Figure 3:**
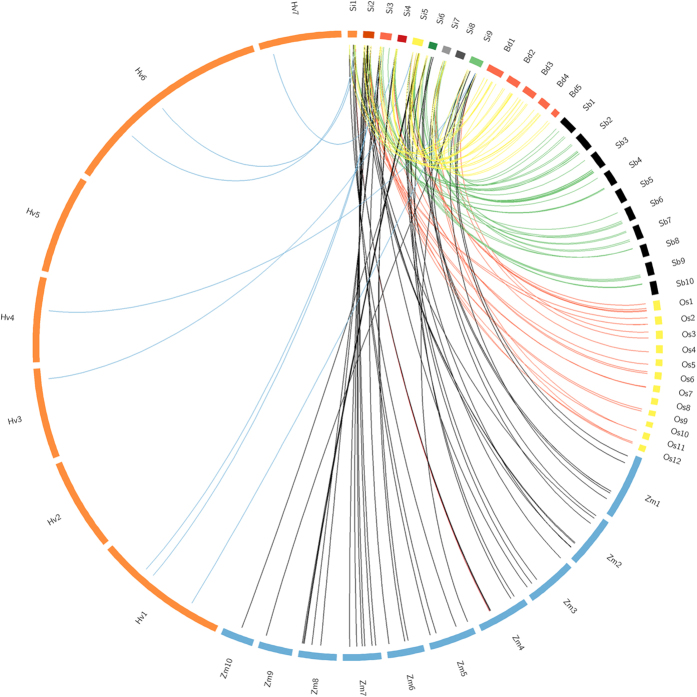
Comparative physical map. Comparative map showing degree of orthologous relationship between *SiSET* genes of foxtail millet (Si) and sorghum (Sb), maize (Zm), rice (Os), barley (Hv) and *Brachypodium* (Bd).

Orthologous gene-pairs for 48 *SiSET* genes (~90%) were identified in *Sorghum bicolor*, *Zea mays, Oryza sativa*, and *Brachypodium distachyon*, which showed maximum synteny with *S. bicolor* (37 genes; 77%), followed by *Z. mays* (34; 70%), *O. sativa* (30; 62.5%), and *B. distachyon* (27; 56.2%) (Fig. 3). The decrease in synteny with increase in phylogenetic distance between the Poaceae members demonstrates the degree of genetic-relatedness of the corresponding genomes. This is in agreement to previous genome-wide analyses performed for *NAC*^34^, *WD40*^35^, *AP2/ERF*^36^, *C*_*2*_*H*_*2*_ *zinc finger* and *MYB* transcription factors^37^,^38^, *DCL, AGO* and *RDR*^39^, *14-3-3* 40, secondary cell wall genes and WRKY transcription factors^41^,^42^, and ADP-ribosylation factor^43^ in foxtail millet. The effect of Darwinian selection in divergence of these genes is examined by investigating the ratio of rate of non-synonymous substitution (Ka) to synonymous substitution (Ks) for all the orthologous gene pairs. The average Ka/Ks ratios of foxtail millet-sorghum, -maize, -rice and -*Brachypodium* were 0.8, 0.2, 0.6 and 0.5, respectively (Supplementary Table 3). The Ka/Ks ratio below 1 for all the orthologous gene-pairs demonstrated that the genes were under intense positive purifying selection, which accorded well with evolutionary analysis of *NAC*, *WD40*, *AP2/ERF*, *C*_*2*_*H*_*2*_ *zinc finger* and *MYB* transcription factors, *DCL, AGO* and *RDR*, *14-3-3*, secondary cell wall genes and WRKY transcription factors, and ADP-ribosylation factor gene families in foxtail millet^34^,^35^,^36^,^37^,^38^,^39^,^40^,^41^,^42^,^43^. The study also revealed the period of divergence of foxtail millet-sorghum, -maize, -rice and -*Brachypodium* as 25.8, 28, 36.5 and 50.7 million years ago, respectively. The evolutionary relationship and sequence identity along with genome evolution suggest that SiSET proteins may possess functions similar to their orthologs in sorghum, maize and rice.

### Promoter analysis

To understand the molecular mechanism of transcriptional regulation of SET genes in *Setaria*, *cis*-elements at the promoter regions were identified using 1.5 kb upstream sequences from the translation initiation codon of *SiSET* genes. Various *cis*-acting elements related to plant growth, development, and stress response were identified in the promoter region of *SiSET* genes (Supplementary Table 4). Many of the *cis*-acting elements were conserved in all the *SiSET* genes which include basic regulatory motifs such as TATA-box and CCAAT box. Stress-responsive motifs such as dehydration responsive *cis*-acting elements (ACGTATERD1), early responsive to dehydration (CURECORECR), copper-response element (WRKY71OS) and pathogenesis-related (GATABOX) were present in most of the upstream region of *SiSET* genes. Hormone-responsive *cis*-elements such as ABA-responsive (ARR1AT), cytokinin-regulated (MYBCORE), ABA-independent (WBOXATNPR1) and SA-responsive motif (TCA, CGTCA and TGACG) were also found at the promoter regions of *SiSET* genes. Similarly, the presence of various cis-acting elements such as MYB recognition, auxin responsive, gibberellin (GA) response, abscisic acid (ABA) responsive and E2F-binding site were reported in the promoter regions of *OsSET* genes of rice^27^. These elements act as key regulators for transcription factors which control various molecular mechanisms such as transcriptional regulation of genes required for DNA replication and cell cycle^44^.

### *In silico* tissue specific-expression profiling of *SiSET* genes

**Figure 4:**
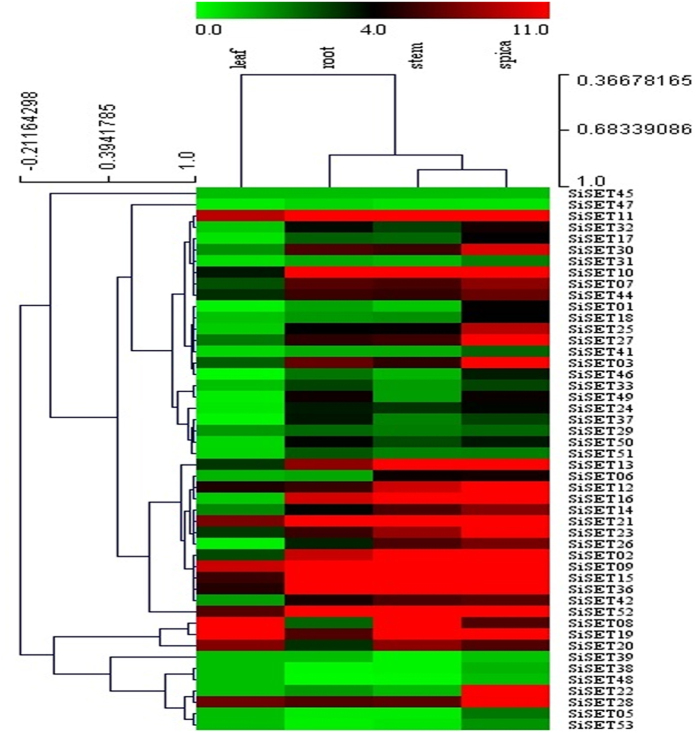
RNA-seq derived tissue-specific expression profiles of *SiSET* genes. Heat map showing the expression pattern of *SiSET* genes in four tissues namely leaf, root, stem and spica. The scale bar at the top represents relative expression values.

*SET* genes catalyze the transfer of methyl groups from the co-factor S-adenosylmethionine (AdoMet) to specific lysine residues of protein substrates, such as the N-terminal tails of histone (H3 or H4) and the large subunit of Rubisco through post-translational modifications such as acetylation, methylation, phosphorylation, ubiquitination, ribosylation and biotinylation. Change in histone modifications could lead to changes in gene transcription in plants. In view of this, expression pattern of all the identified *SiSET* genes was analyzed in four tissues namely root, leaf, stem and spica using the RNA-sequence data (Fig. 4). The heatmap showed that ~50% (25) of SET genes were highly expressed in spica, whereas in leaves, only 15% (8) genes showed higher level of expression. Moreover, ~61% (31) *SiSET* gene showed less or moderate expression in leaf. *SiSET09*, *SiSET11* and *SiSET22* were found to be highly expressed in all the four tissues. In addition, the expression profiles of segmentally duplicated genes were compared to analyse whether these genes show similar expression patterns. However, no such similarities were observed as the duplicated gene pairs also showed differential expression patterns among them. Altogether, differential expression of *SiSET* genes in root, leaf, stem and spica supports the assertion that these genes have roles in development. In addition, 20 *SiSET* genes showed high expression patterns during inflorescence and shoot development. Proliferation-differentiation cell transition, and gametogenesis and embryogenesis occur in these organs and these processes are reported to be under epigenetic control^22^.

### Expression profiling in response to abiotic stress and hormonal treatments and heterologous expression of *SiSET14* in yeast

**Figure 5:**
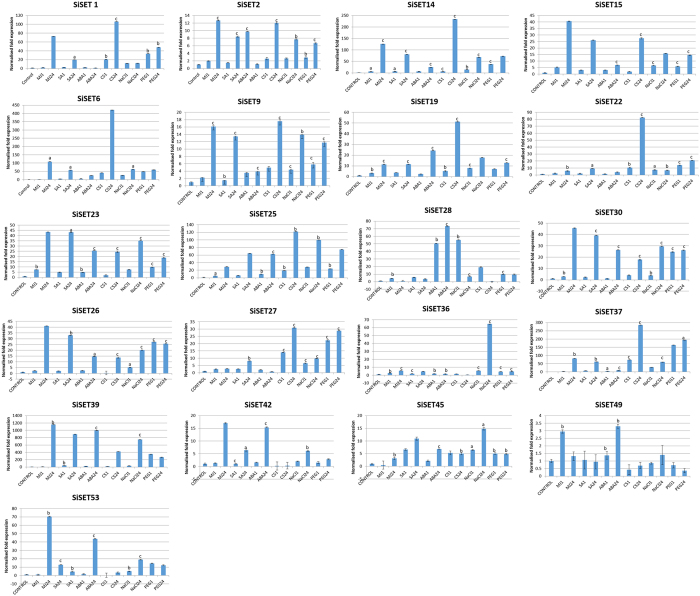
Expression profiles of SiSET genes in response to abiotic stress and hormonal treatments. Relative gene expression of candidate 21 SiSET genes analysed by qRT-PCR in response to salinity (NaCl), dehydration (PEG), cold stress and hormone (ABA, Salicylic acid and Methyl Jasmonate) treated conditions (1 h and 24 h samples) in foxtail millet has been shown. *Act2* was used as an internal control to normalize the data. The Y-axis indicates the relative expression level and error bars represent standard deviation calculated based on three technical replicates (^*a*^*P* < 0.05, ^*b*^*P* < 0.01, ^*c*^*P* < 0.001).

Expression patterns of twenty-one candidate *SiSET* genes showing differential expression patterns in heatmap was performed to understand the transcript abundance of respective genes generated in response to several abiotic stress (cold, salinity and dehydration) and hormonal treatments (methyl jasmonate, salicylic acid and abscisic acid) (Fig. 5). qRT-PCR results showed that 18 *SiSET* genes were highly expressed after 24 h of treatment with methyl jasmonate and salicylic acid, whereas lower level of expression was observed after an hour of treatment. On the contrary, no significant difference in expression pattern of *SiSET19*, *SiSET23*, *SiSET30* and *SiSET36* were observed in response to methyl jasmonate and salicylic acid at 1 h and 24 h post-treatment. Interestingly, most of the *SiSET* genes were up-regulated (10 to 400-fold change as compared to control) under cold stress treatment while *SiSET28* and *SiSET45* were down-regulated during late hour (24 h). The expression of *SiSET14*, *SiSET15*, *SiSET25*, *SiSET36* and *SiSET37* were up-regulated (10 to 600-fold change as compared to control) in response to salt treatment during late hour (24 h). Contrarily, up-regulation (55.2 folds) was observed in *SiSET28* during early hour (1 h) upon salt treatment. During dehydration stress, most of the *SiSET* genes were up-regulated except *SiSET49*, which was down-regulated (Fig. 5). Similarly, stress-induced expression of *SET* genes has been reported in many plant species including Arabidopsis, rice, maize and *Brassica rapa*^27^,^29^,^30^,^31^. In maize, five genes namely *Zmset3, Zmset27, Zmset29, Zmset31* and *Zmset40* exhibited differential expression in response to PEG and NaCl treatments^29^. Similarly, responses of OsSET genes under various phytohormone (including Naphthaleneacetic acid, NAA; Kinetin, KT; and GIBBERELLIC ACID; GA3) treatments showed that nine *OsSET* were differentially expressed^27^.

**Figure 6:**
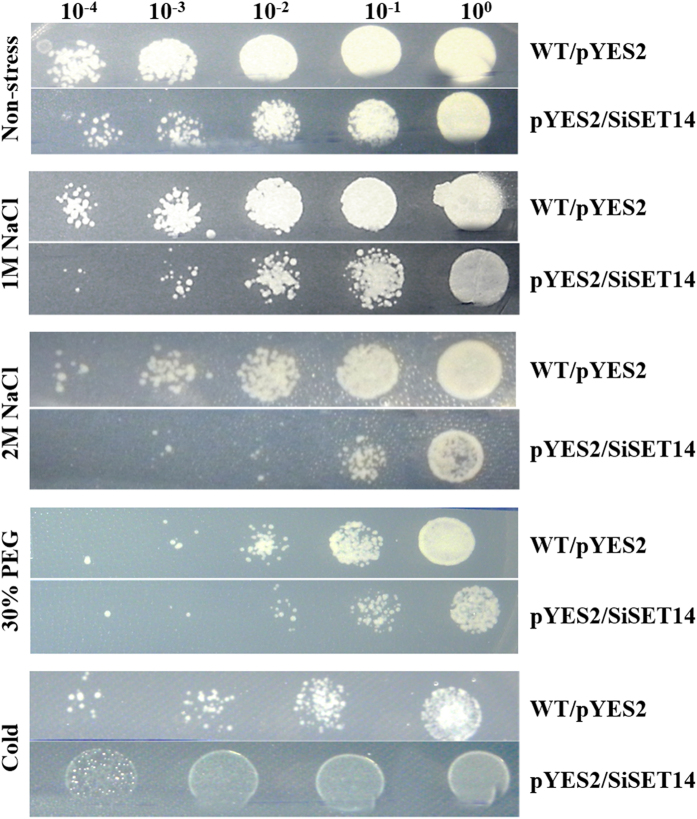
Heterologous expression of *SiSET14* in yeast cells. Yeast cells transformed with *SiSET14* subjected various abiotic stresses (1 M NaCl, 2 M NaCl, 30% PEG and 4 °C cold treatment) and growth was monitored.

qRT-PCR data indicated that *SiSET14* showed several fold upregulated expression during late phase of all the stress conditions in comparison to controlled conditions. Based on this, *SiSET14* was chosen for its over-expression in yeast. The yeast cells expressing *SiSET14* proteins showed distinguishable difference in growth rate in comparison to non-transformed yeast cells (Fig. 6). During non-stressed conditions, growth rate of yeast cells harbouring *SiSET14* was comparatively better than non-transformed cells. However, during stress, the proliferation of *SiSET14* transgenic cells sharply decreased in response to NaCl and PEG in comparison to wild type cells. Further, the *SiSET14* transgenic cells showed comparatively better growth in response to cold stress (Fig. 6).

Arabidopsis homolog of *SiSET14* (ASHH1) has been reported to have H3K36 methyltransferase activity, which is involved in plant reproductive developments^45^. In addition to having lysine-methyltransferase activity, SET domain containing-proteins regulate gene expression by intra- and inter-molecular interactions with other proteins during plant growth and development. *In silico* prediction of protein-protein interactions using STRING showed that *SiSET14* protein interacts with several polycomb group genes and HISTONE DEACETYLASE for regulation of methylation activities (Supplementary Fig. 8). Interactions of MEA, CLF and SWN with SET domain containing genes may be linked with various developmental processes, as mutations in the interacting partners display phenotypes defective of affecting similar developmental pathways^46^,^47^. Further, regulation of histone methylation activities through interactions with MEA, CLF, SWN and HISTONE DEACETYLASE which are involved in various developmental process requires experimental validation.

### Methylation analysis of SiSET genes

**Figure 7:**
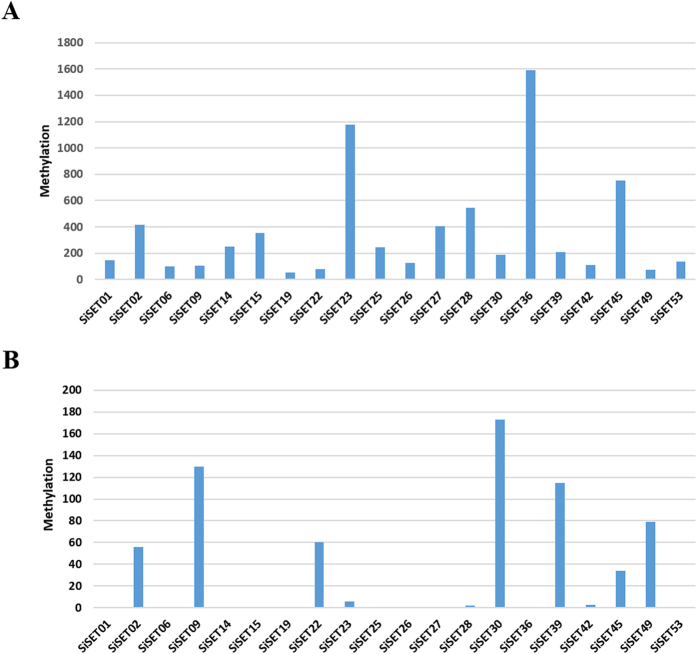
Number of cytosine methylation in 21 SiSET genes which are differentially expressed under abiotic stress in foxtail millet. (**A**) mC in gene body, (**B**) mC in promoter region.

Genome-wide methylation profiling at single-nucleotide resolution was performed which showed that DNA methylation occurs in and around genes as well as intergenic regions of the genome (data not shown). In the present study, methylation level in *SET* domain-containing genes has been investigated and found that most of the genes were hypermethylated. Methylation level in TSS (transcription start site) region of *SiSET* genes was also analysed and found hypermethylated at TSS region of 7 *SiSET* genes (Fig. 7; Supplementary Table 5). Correlating the qRT-PCR expression data with methylation data showed that highly differentially regulated *SiSET* genes possess hypermethylation in gene body whereas methylation events were completely absent in TSS region. For example, *SiSET01* showed a differential expression in response to various abiotic stresses, and methylation analysis revealed the absence of methylation signature in TSS region; however, increased methylation (65%) was observed in gene body. The result suggests demethylation events would have occurred in *SiSET01* when exposed to abiotic stresses which certainly increased the expression of gene. In contrast, down-regulated genes showed hypermethylation signature in TSS region. For instance, reduced differential expression of *SiSET02* was observed during various abiotic stresses, but increased methylation (14%) was present in TSS region as well as gene body. These results suggest that increased methylation might supress the expression of a gene; however, further experiments are required for validation.

Histone modification and DNA methylation processes could also act as epigenetic modifications in plant DNA, and it is well known that DNA and histone lysine methylation systems are highly interrelated and rely on each other for normal chromatin function *in vivo*. Non-methylated DNA in CpG islands acts as a part of genomic signature to recruit H3K4 and H3K27 trimethylation and exclude H3K36 methylation, thus creating chromatin environments unique to gene regulatory elements that are able to modulate transcriptional states. During DNA replication, Dnmt1 (DNA methyltransferase) associates with Uhrf1 which is multidomain protein containing several chromatin binding domains including PHD and SRA domains that bind to H3K9me3/me2/H3K4me0 and hemimethylated DNA, respectively^48^. The observations reported in the present study also corroborated with the above, as *SiSET* gene modulation strictly depends on either methylation in promoter or gene body regions.

## Conclusions

In recent years, several reports have been published stating that SET domain proteins are encoded by a large multigene family in plants and therefore, investigation of their functional roles will delineate the epigenetic control of gene regulation in plants. In view of this, the study has been performed to identify and characterize SET domain-containing proteins in foxtail millet. A total of 53 *SiSET* genes were identified and the corresponding proteins were analysed for their physico-chemical properties. The characteristics including gene/protein length, intron-exon distribution and position, chromosomal localization, domain composition and protein properties were observed to be highly variable among these genes. Phylogenetic tree classified these proteins into five groups (I–V). Comparative mapping of SET genes between the genomes of *S. bicolor*, *Z. mays, O. sativa* and *B. distachyon* revealed decrease in synteny with increase in phylogenetic distance between the Poaceae members, and demonstrated the degree of genetic-relatedness of the corresponding genomes. *In silico* expression profiling of *SiSET* genes showed their differential expression in root, leaf, stem and spica which supported the assertion that these genes have roles in development. Expression profiling of twenty-one candidate *SiSET* genes in response to several abiotic stress (cold, salinity and dehydration) and hormonal treatments (methyl jasmonate, salicylic acid and abscisic acid) was performed. The study showed differential expression of these genes during late phase of stress and hormonal treatments with significant upregulation of *SiSET14* gene during cold stress. Heterologous expression of *SiSET14* in yeast system conferred tolerance to transgenic yeast cells challenged with different stresses. Methylation analysis of *SiSET* genes showed hypermethylation in gene body of highly differentially expressed genes, whereas methylation event was completely absent in their transcription start sites. This suggested the occurrence of demethylation events during various abiotic stresses, which enhance the gene expression. These findings provide novel insights into the putative roles of *SiSET* genes in conferring abiotic stress tolerance in this climate resilient model crop, foxtail millet. At present, over-expression of candidate genes in rice and foxtail millet systems are in progress. This would throw light on understanding the role of *SiSET* genes in conferring stress tolerance, their potential clients and functional interaction in response to specific stress.

## Materials and Methods

### Identification of *SiSET* gene family

Protein sequences of SET domain-containing genes reported in *Zea mays*^29^, *Oryza sativa*^27^, *Arabidopsis thaliana*^23^ and *Brassica rapa*^30^ were retrieved and HMM profiles were prepared for HMM search against *Setaria italica* protein sequence database using HMMER program (v2.3.2)^49^. All the sequence information (gene, transcript, CDS and protein) was collected from GFF file of *Setaria italica* genome available at Phytozome database (http://phytozome.jgi.doe.gov/pz/portal.html). All the putative proteins were searched for the signature SET domain using PROSITE (http://prosite.expasy.org/), SMART (http://smart.embl-heidelberg.de/), PFam (http://pfam.xfam.org/search), NCBI Batch CD search tool (http://www.ncbi.nlm.nih.gov/Structure/bwrpsb/bwrpsb.cgi/) and PIECE (http://wheat.pw.usda.gov/piece/). The physicochemical properties of SET proteins were then examined using ExPASy ProtParam tool (http://web.expasy.org/protparam/). Motif identification was performed using MEME (http://meme-suite.org/) using the following parameters; (i) number of repetitions: any; (ii) maximum number of motifs: 6; and (iii) optimum width of the motif: 50.

### Gene structure prediction, chromosomal localization and phylogenetic analysis

Chromosomal coordinates for individual *SiSET* genes were obtained from Phytozome database, and the genes named as *SiSET01* to *SiSET53* on the basis of their physical location on nine chromosomes ranging from short arm telomere to long arm telomere. The distribution of exons and introns in the genes were graphically represented using Gene Structure Display Server (http://gsds.cbi.pku.edu.cn/) by comparing their full-length coding sequence (CDS) with their corresponding genomic sequence. MapInspect (http://www.plantbreeding.wur.nl/UK/ software_mapinspect.html) was used to physically map the *SiSET* genes onto chromosomes.

Multiple sequence alignment of all putative SiSET proteins was done for conserved regions of PreSET, SET and PostSET domains with BioEdit tool (http://www.mbio.ncsu.edu/bioedit/bioedit.html) with default parameters. Further, the phylogenetic analysis was performed with RAxML v8.0.0 and tree was constructed using maximum likelihood method^50^. The stability of nodes was tested by bootstrap analysis with 1000 replicates. To identify the conversed-ness of SiSET protein, STRING (http://string-db.org/) was used to show the presence or absence of linked proteins across species.

### Comparative mapping, gene duplication and evolutionary relationships

The amino acid sequences of SiSET proteins were BLASTP searched against the proteome of sorghum (Sb; *Sorghum bicolor*), maize (Zm; *Zea mays*) and rice (Os; *Oryza sativa*) and Brachypodium (Bd; *Brachypodium distachyon*). Resultant hits with E-value ≤ 1e-5 and a minimum of 80% identity were considered as significant syntenic proteins and reciprocal BLAST was performed to identify the true orthologs. MCScanX was used to predict the segmental duplications^51^ and tandem duplications were examined manually in the physical map. The synonymous (Ks) and non-synonymous (Ka) substitution rates for the gene-pairs were calculated using PAL2NAL^52^. Ks and Ka value were calculated by following Muthamilarasan *et al.*^43^. The time of duplication and divergence in terms of million years ago (Mya) was estimated using a synonymous mutation rate of λ substitutions per synonymous site per year as T = Ks/2λ (λ = 6.5 × 10^−9^)^53^.

### Functional annotation and promoter analysis

Blast2GO tool (https://www.blast2go.com/) was used for functional annotation of SiSET proteins. The amino acid sequences were loaded in the Blast2GO interface to perform BLASTP against the non-redundant protein database of NCBI, followed by mapping and retrieval of GO terms associated with the BLAST results. Finally, annotation of GO terms associated with each query sequence to known protein function was performed. Co-expression network was constructed using BiNGO plugin of Cytoscape v2.6 following Muthamilarasan *et al.*^42^. The upstream sequences (2 kbp) of *SiSET* genes were investigated for the presence of *cis-*regulatory elements using PLACE database (http://www.dna.affrc.go.jp/PLACE/signalup.html).

### *In silico* expression profiling of *SiSET* genes

Illumina RNA-HiSeq data of 4 tissues namely spica, stem, leaf and root were retrieved from European Nucleotide Archive [SRX128226 (spica); SRX128225 (stem); SRX128224 (leaf); SRX128223 (root)]. The reads were filtered and normalized by RPKM (reads per kilobase per million) as described by Mishra *et al.*^35^ and heatmap was generated using TIGR MultiExperiment Viewer (MeV4.9.0) software^54^.

### Plant materials, treatments, RNA isolation and qRT-PCR

Foxtail millet cultivar “IC403579” (known for drought and salinity tolerance^41^) was grown in greenhouse conditions (16 h dark/8 h light at 25–28 °C). Twenty-one days old seedlings were subjected to cold (4 °C), drought (20% PEG), salinity (250 mM NaCl), abscisic acid (ABA; 100 mM), methyl jasmonate (MeJA; 100 mM) and salicylic acid (SA; 100 mM) following Muthamilarasan *et al.*^41^, and samples were collected at 0, 1 and 24 h post-treatments. The tissues were immediately frozen in liquid Nitrogen and stored at −80 °C. Total RNA was isolated using a TRI Reagent (Sigma) followed by RNase-free DNase I (Qiagen) treatment for removal of DNA contamination. First strand cDNA from total RNA (1 μg) was synthesized with M-MuLV Reverse Transcriptase (Promega), according to the manufacturer’s instructions. Reaction mixture for Real-time quantitative PCR was prepared following Yadav *et al.*^39^ and the reaction was carried out on ABI StepOne^TM^ Real-time PCR instrument (Applied Biosystems) in three technical replicates for each biological triplicate using the primers listed in Supplementary Table 6. The reactions were carried out according to the following temperature profile: 95 °C for 30 seconds, 40 cycles of 95 °C for 5 seconds, and 60 °C for 34 seconds. A constitutive *Act2* gene-based primer was used as endogenous control^55^. The quantity of transcript accumulated for each gene was normalized with the internal control and 2^−ΔΔCT^ was calculated following Kumar *et al.*^55^. Effect of different abiotic stresses on abundance of transcript was statistically examined using ANOVA with GraphPad Quick calculator (http://www.graphpad.com/quickcalcs/ttest1.cfm). The differences in the effects of stress treatments on various parameters in *SiSET* genes under study were considered statistically significant at ^*a*^*P* < 0.05, ^*b*^*P* < 0.01, ^*c*^*P* < 0.001.

### Heterologous expression of *SiSET* in *Saccharomyces cerevisiae* and stress tolerance assay

Full length ORF of *SiSET14* (Si; *Setaria italica*) was cloned into yeast expression vector pYES2 (Invitrogen, USA) at EcoRI/BamHI restriction site. The recombinant plasmids containing *SiSET14* (pYES2-*SiSET14*) and plasmid without insert (pYES2-0) were transformed separately in yeast strain W303 using Yeastmaker Yeast Transformation System (Clontech) and transformants were screened by growth of colonies on SD/-ura medium with 2% (w/v) dextrose at 30 °C for 3 days. Stress tolerance assay was performed by following the methods of Shukla *et al.*^56^. In brief, yeast cells harbouring recombinant plasmid pYES2-*SiSET14* and empty vector were grown in SD/-Ura broth and incubated at 30 °C for 24 h. The OD_600_ was adjusted to 1.0 and 10 ml of induction medium consisting of SD/-Ura broth supplemented with 2% galactose was added. The culture was then incubated for 36 h to promote the expression of *SiSET14* gene. After 36 h, the culture was adjusted to OD_600_ 0.6, and inoculated in 10 ml SD/-Ura, each containing 1 M NaCl, 2 M NaCl and 30% PEG. The plates were incubated at 30 °C for 36 h. For cold stress, the plates were incubated at 4 °C for 36 h. After stress treatments, the cultures were serially diluted (10^0^, 10^−1^, 10^−2^, 10^−3^, 10^−4^) and 7 μl of each diluted cells were spotted on basal SD/-Ura medium (supplemented with 2% w/v dextrose) and incubated at 30 °C for 3 days. For the control, an equivalent number of unstressed cells suspended in SD/-Ura broth were spotted on SD/-Ura plates.

### Methylation analysis of *SiSET* genes

For methylation analysis, total genomic DNA isolated from foxtail millet cultivar “IC403579” using standard protocol was fragmented by sonication (100–300bp) and the fragmented DNA was end-repaired and adapters were ligated by following the Illumina manufacturer’s instructions (Illumina, San Diego, CA). The adapter ligated DNA fragments were then treated with sodium bisulfite using EZ DNA Methylation-GoldTM kit (Zymo Research Corporation, CA, USA) to convert unmethylated cytosines to uracils. After desalting, the bisulfite converted DNA were processed for size selection followed by PCR amplification and then libraries were analysed for quality check. The qualified libraries were sequenced using the Illumina HiSeq 2000 system according to manufacturer’s instructions. Raw sequences were filtered to remove the low quality reads, and methylated nucleotides were identified using *Setaria italica* reference genome by following Garg *et al.*^57^.

## Additional Information

**How to cite this article**: Yadav, C. B. *et al.* Comprehensive analysis of SET domain gene family in foxtail millet identifies the putative role of *SiSET14* in abiotic stress tolerance. *Sci. Rep.* **6**, 32621; doi: 10.1038/srep32621 (2016).

## Supplementary Material

Supplementary Information

## Figures and Tables

**Table 1:** Comparison of gene structure of SET genes in ten plant species.

Plant Species	No. of SET genes	Exon count	Intron count	Max. number of exons per gene	Avg. number of exons per gene	Avg. length (bp)		Total length (bp)		Shortest (bp)			Longest (bp)		
						exon	intron	exon	intron	exon	intron	exon	intron	exon	intron
*S. italica*	53	664	592	26	12.76	260.6	344.38	172423	203529	3	31	296	3831	3281	14268
*O. sativa*	49	448	397	25	9.14	263.27	355.31	117947	141060	19	61	1610	3533	3957	14282
*S. bicolor*	60	667	610	25	11.11	310.42	530.81	209225	323800	27	67	2274	4070	13036	34103
*Z. mays*	94	1029	904	25	10.94	352.67	640.23	362902	578770	2	1	755	16132	16498	44551
*B. distachyon*	80	921	837	25	11.51	249.78	475.74	230053	398196	27	55	1628	3397	4860	16035
*A. thaliana*	64	554	495	26	8.65	230.88	138.46	127909	68538	11	64	938	2834	1528	11181
*G. max*	217	2513	2302	26	11.58	272.48	710.55	684747	1635688	2	31	549	3733	14064	29719
*S. lycopersicon*	53	393	348	24	7.41	262.00	606.53	102969	211075	3	41	167	3168	4123	22198
*P. trichocarpa*	125	1556	1429	24	12.44	242.87	389.10	377908	556034	12	25	1369	3738	5203	18161
*S. moellendorffii*	18	302	273	24	16.77	177.84	72.32	53710	19745	10	23	365	2146	718	5086
