# Supplementary material for: Comprehensive analysis of SET domain gene family in foxtail millet identifies the putative role of SiSET14 in abiotic stress tolerance
Source: Sci Rep. 2016 Sep 2;6:32621. doi: 10.1038/srep32621 (PMC5009302; doi:10.1038/srep32621)
Supplement: Supplementary Information [file srep32621-s1.pdf]

Comprehensive analysis of SET domain gene family in foxtail millet identifies the putative role of *SiSET14* in abiotic stress tolerance

Chandra Bhan Yadav, Mehanathan Muthamilarasan, Anand Dangi, Shweta Shweta, Manoj Prasad*

National Institute of Plant Genome Research, Aruna Asaf Ali Marg, New Delhi – 110067, India

***Corresponding author**
Manoj Prasad
National Institute of Plant Genome Research
Aruna Asaf Ali Marg
New Delhi 110067
India
E-mail: manoj_prasad@nipgr.ac.in

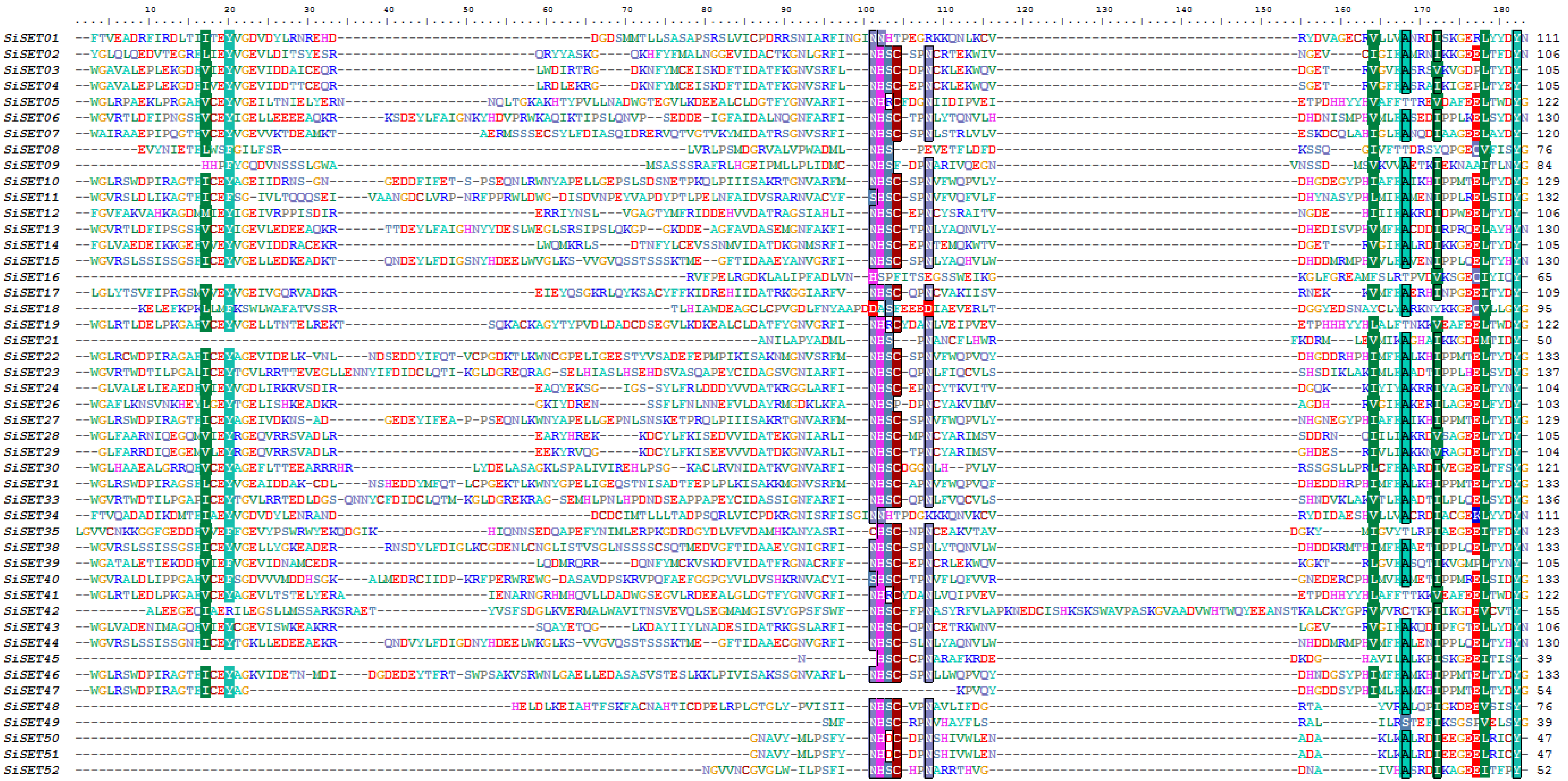

Supplementary Figure 1. Multiple sequence alignment of SET domain of *SiSET* genes.

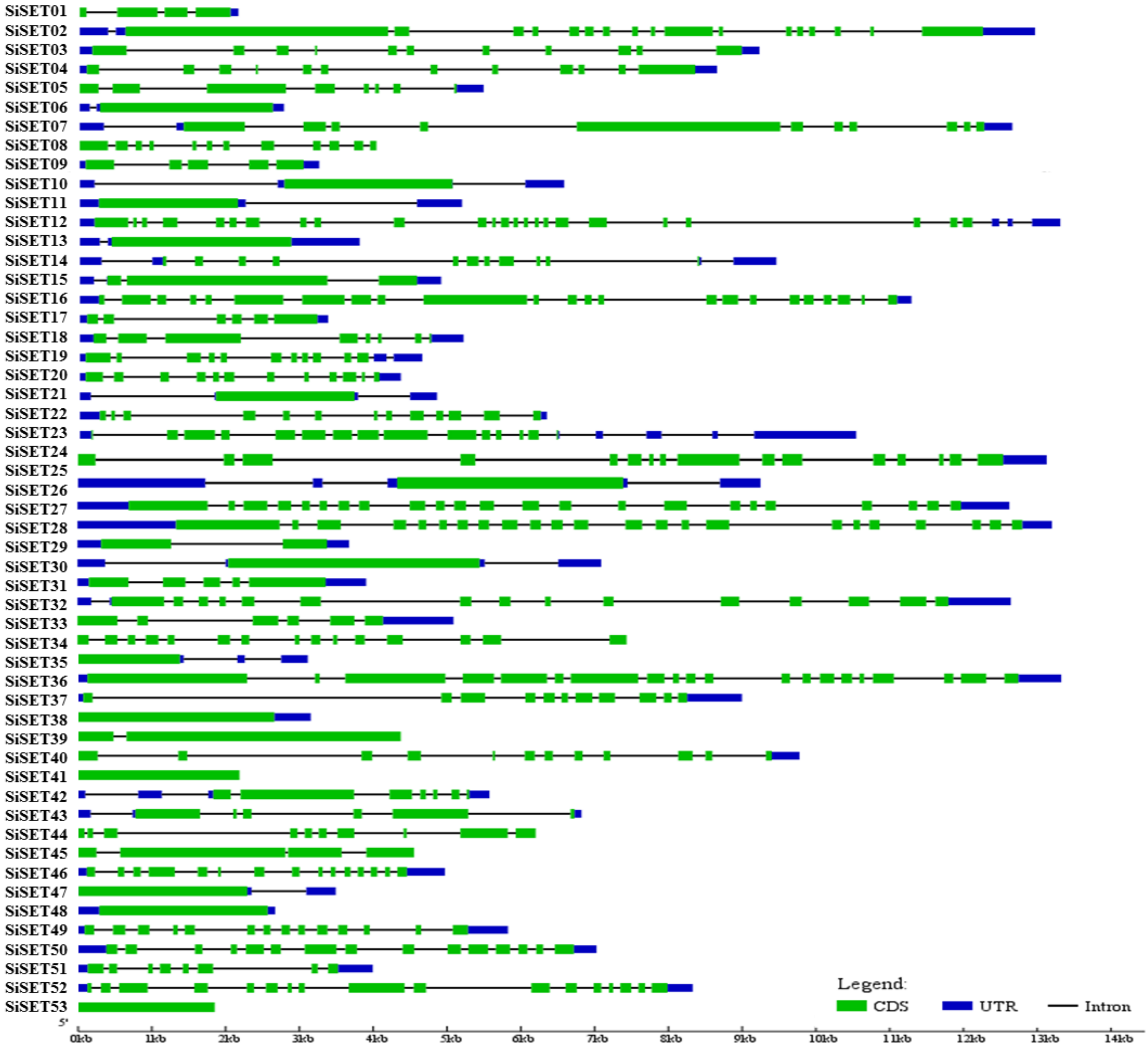

Supplementary Figure 2. Intron-exon positioning of all the 53 SiSET genes.

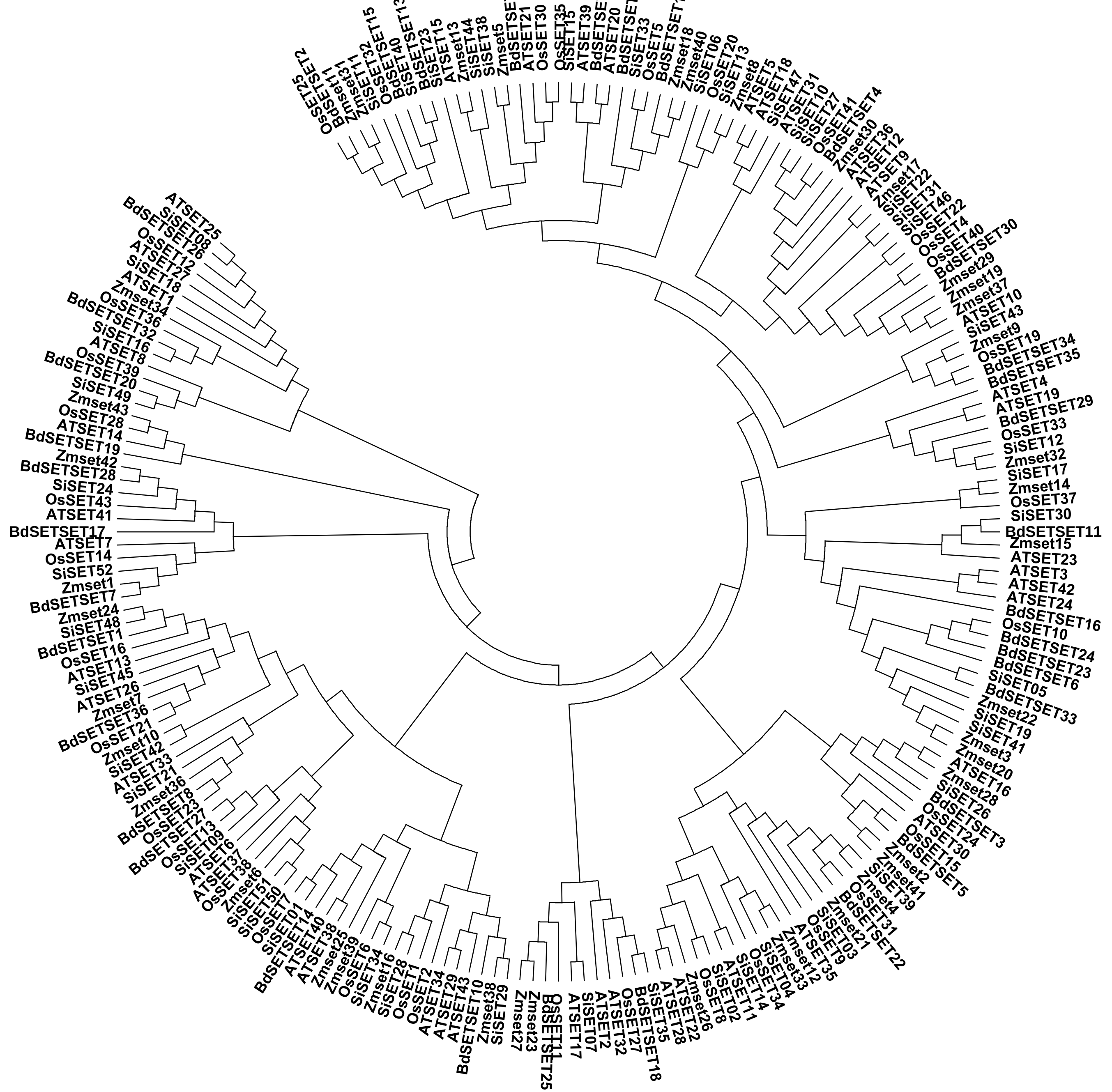

Supplementary Figure 3. Phylogenetic tree showing relationship between SiSET, AtSET, SlSET and OsSET proteins.

A

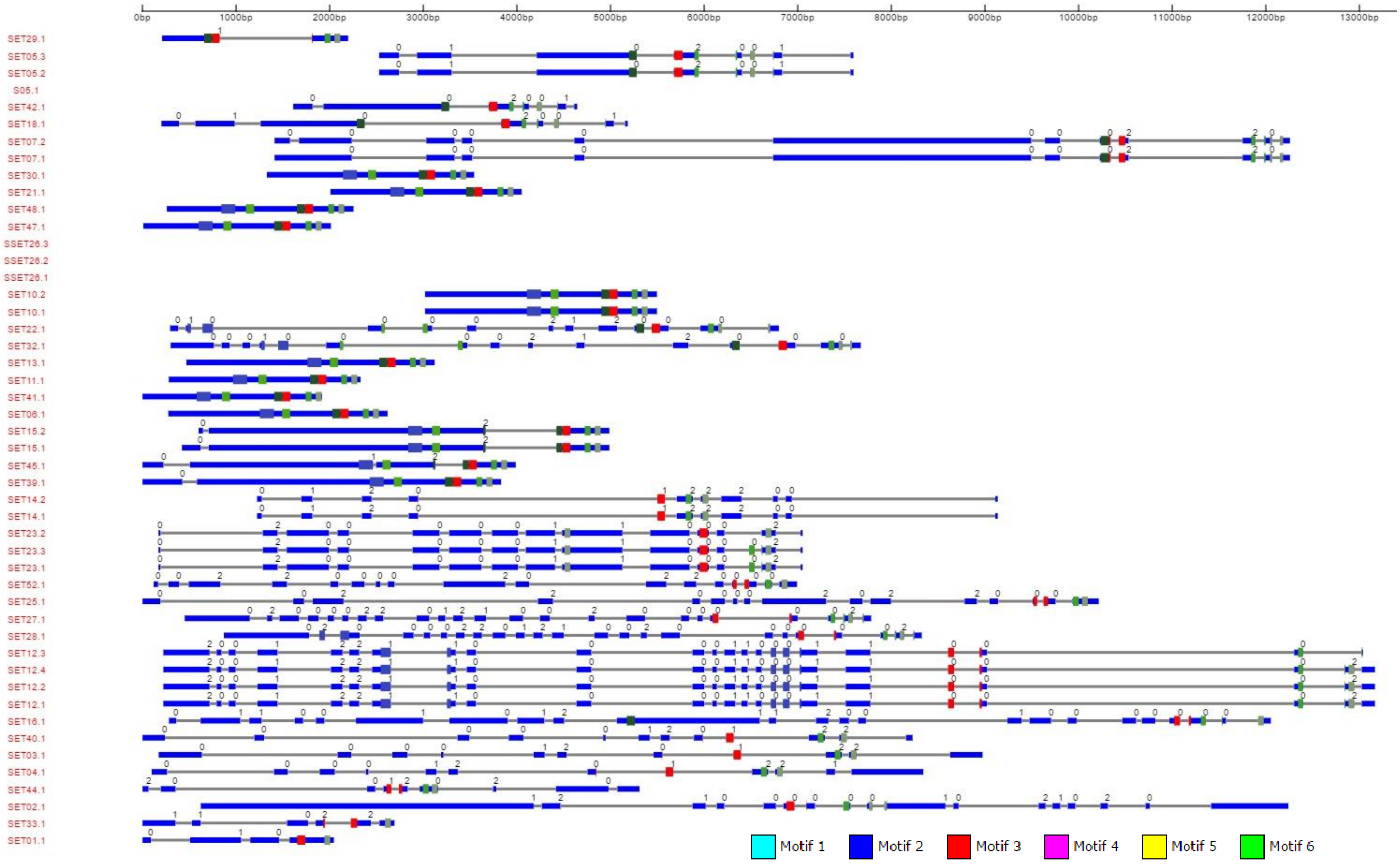

Supplementary Figure 4A. Motif analysis of SiSET genes. Six motifs were identified in all the SiSET genes. Numbers 0, 1, 2 indicate the type of motif corresponding to the colour code given in the figure.

B

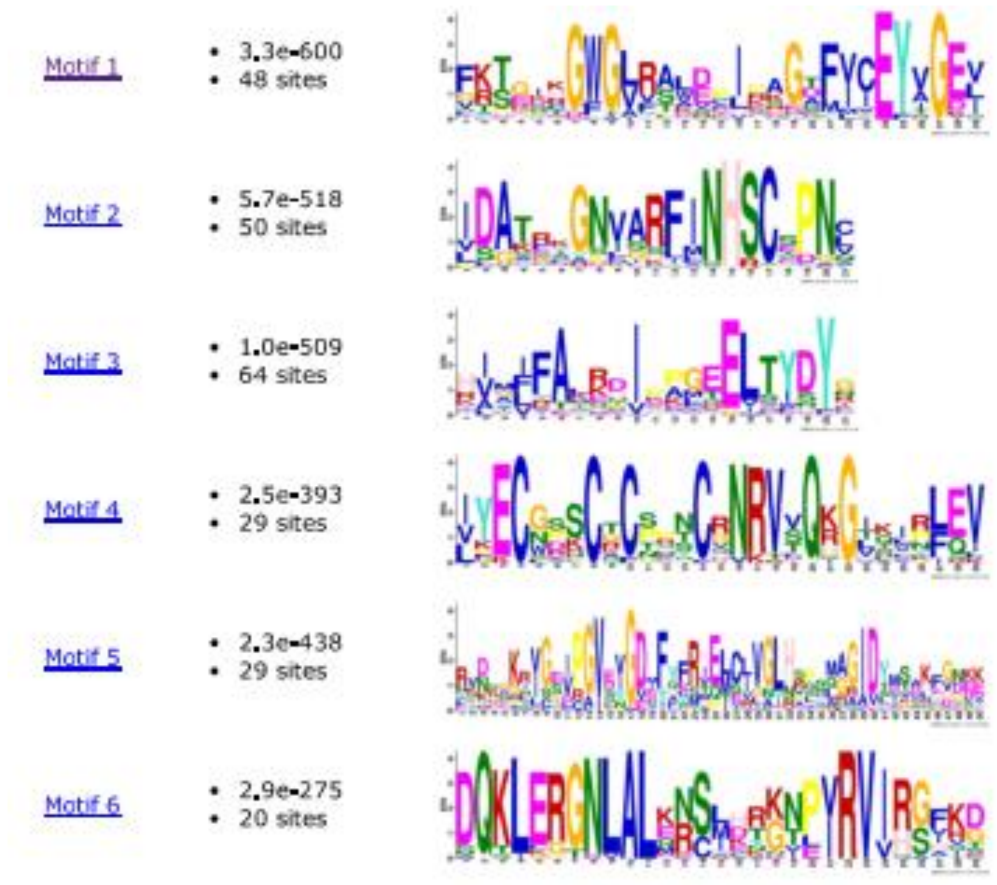

Supplementary Figure 4B. Sequence LOGOs for each motif of SET domains developed using the MEME algorithm.

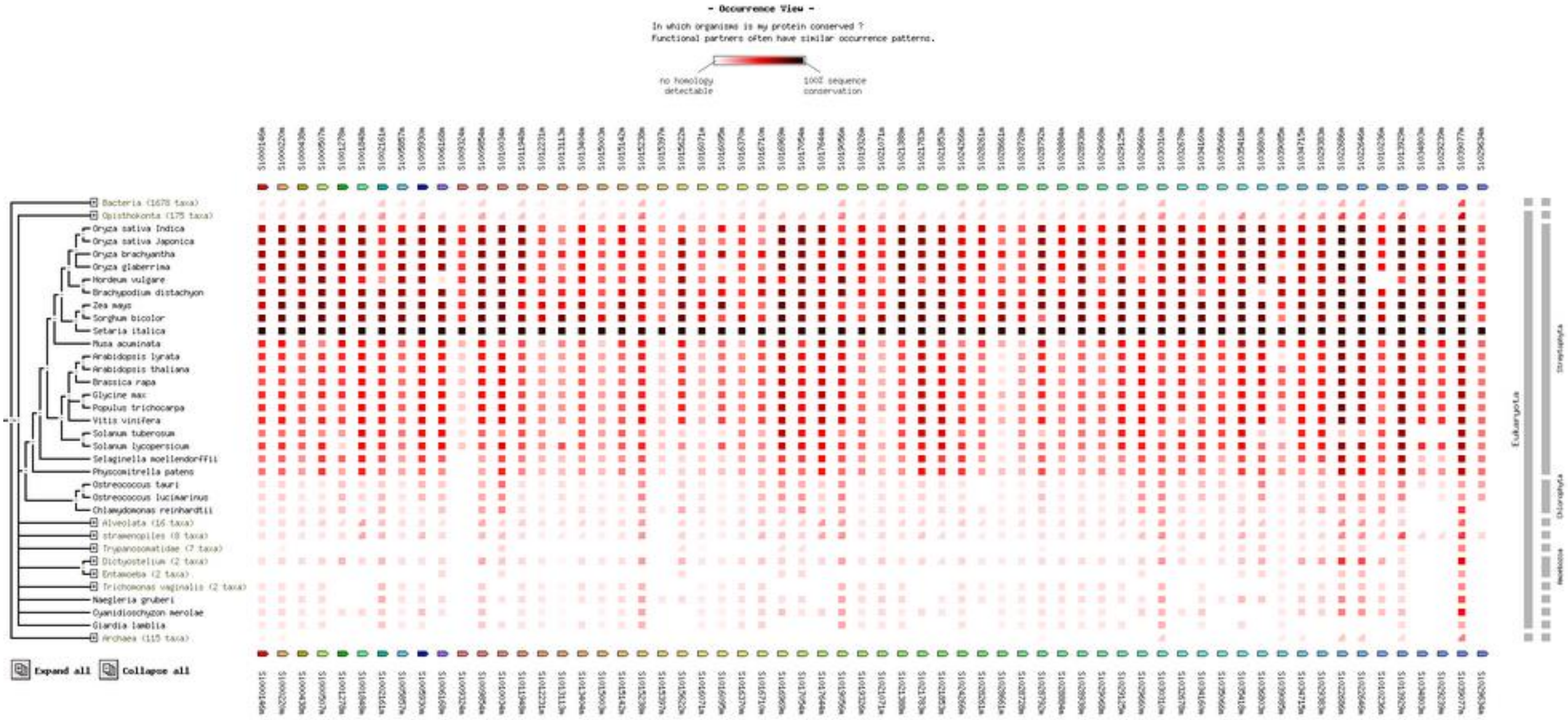

Supplementary Figure 5. Phylogenetic tree and protein converseness of SiSET genes among plant and animal kingdom. The names of species are mentioned at the left hand side. In the subsequent grid, the presence of the protein in a species is marked with a red square and absence with a white space. The intensity of the color of the red square reflects the amount of conservation of the homologous protein in the species.

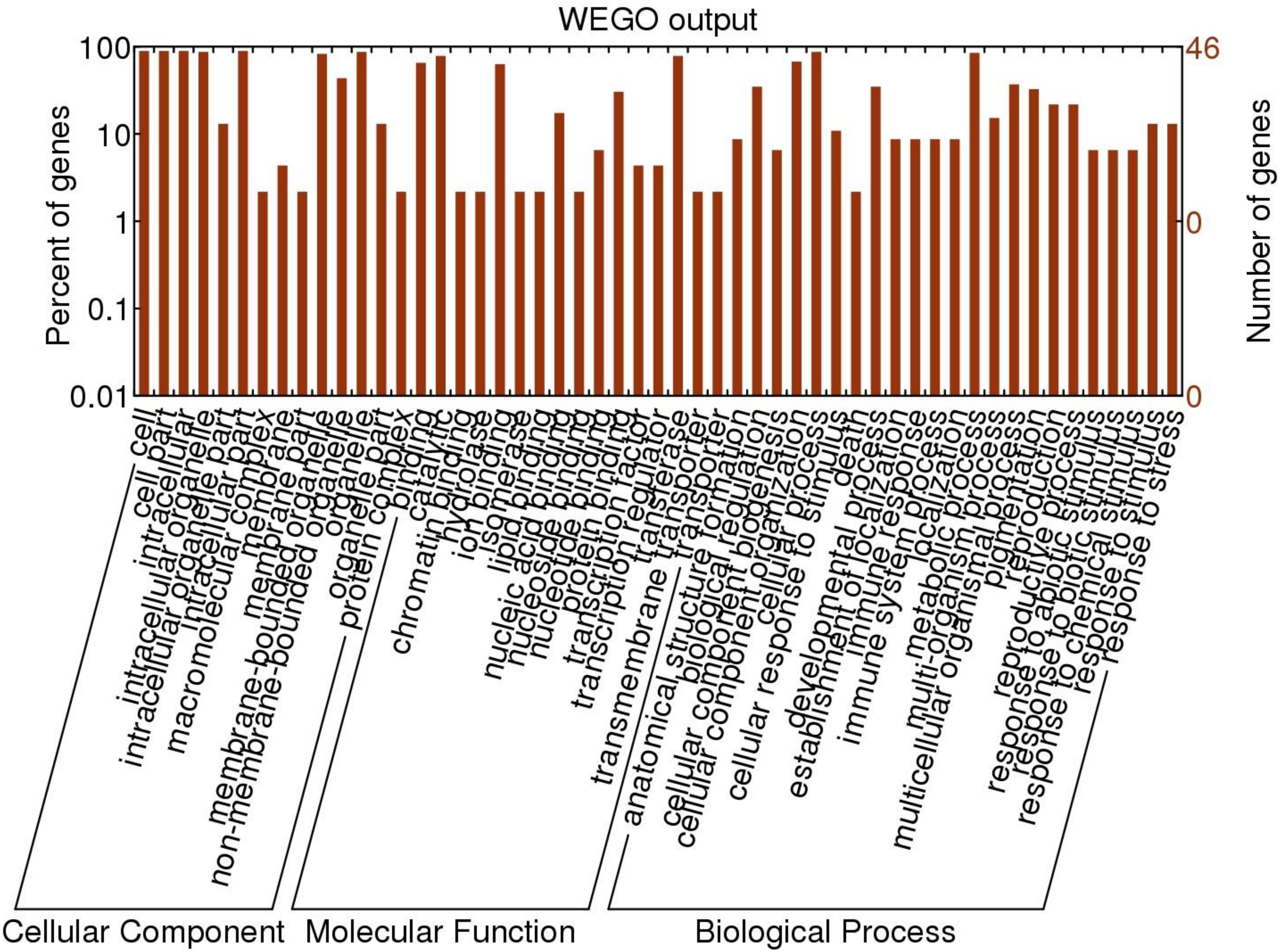

Supplementary Figure 6. Gene Ontology annotation data of biological processes, molecular function and cellular components for SiSET genes.

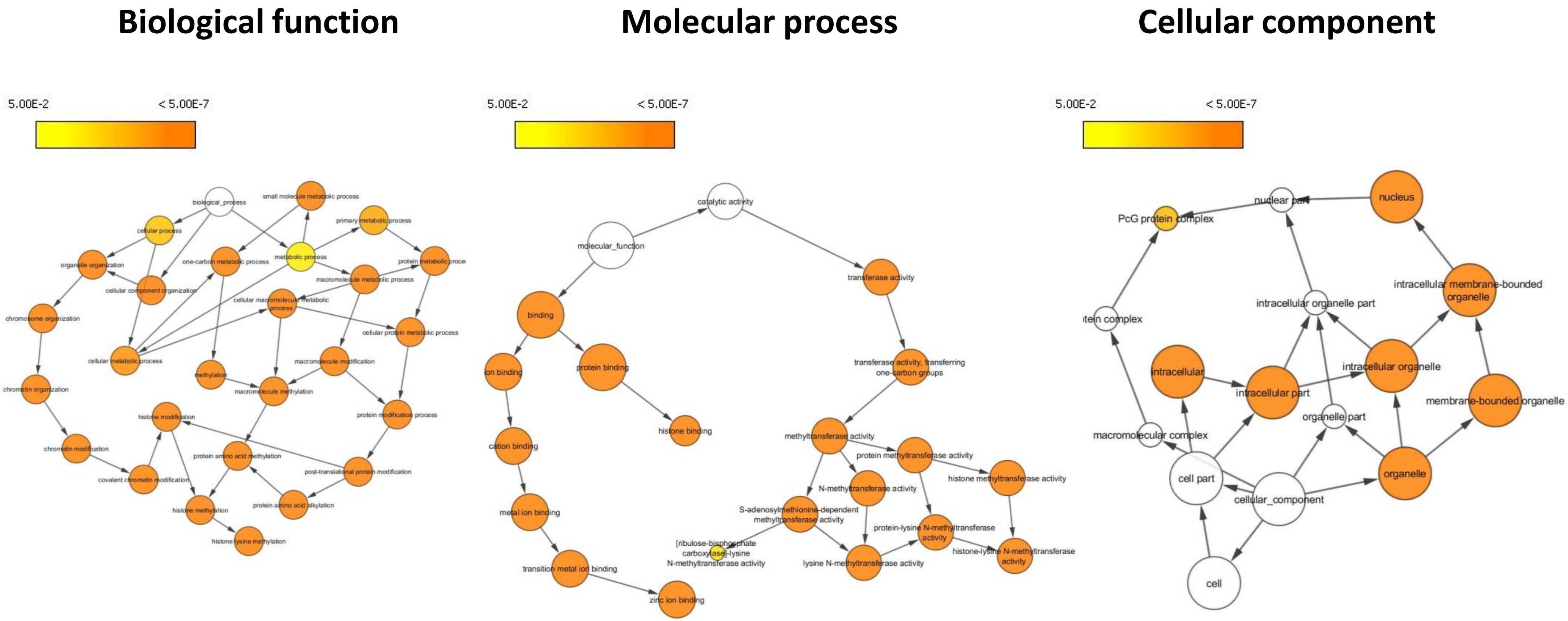

Supplementary Figure 7. Co-expression network of SiSET genes. The number of genes falling in each GO category is directly proportional to the node size. The nodes are colour shaded according to the significance level (corrected P-value).

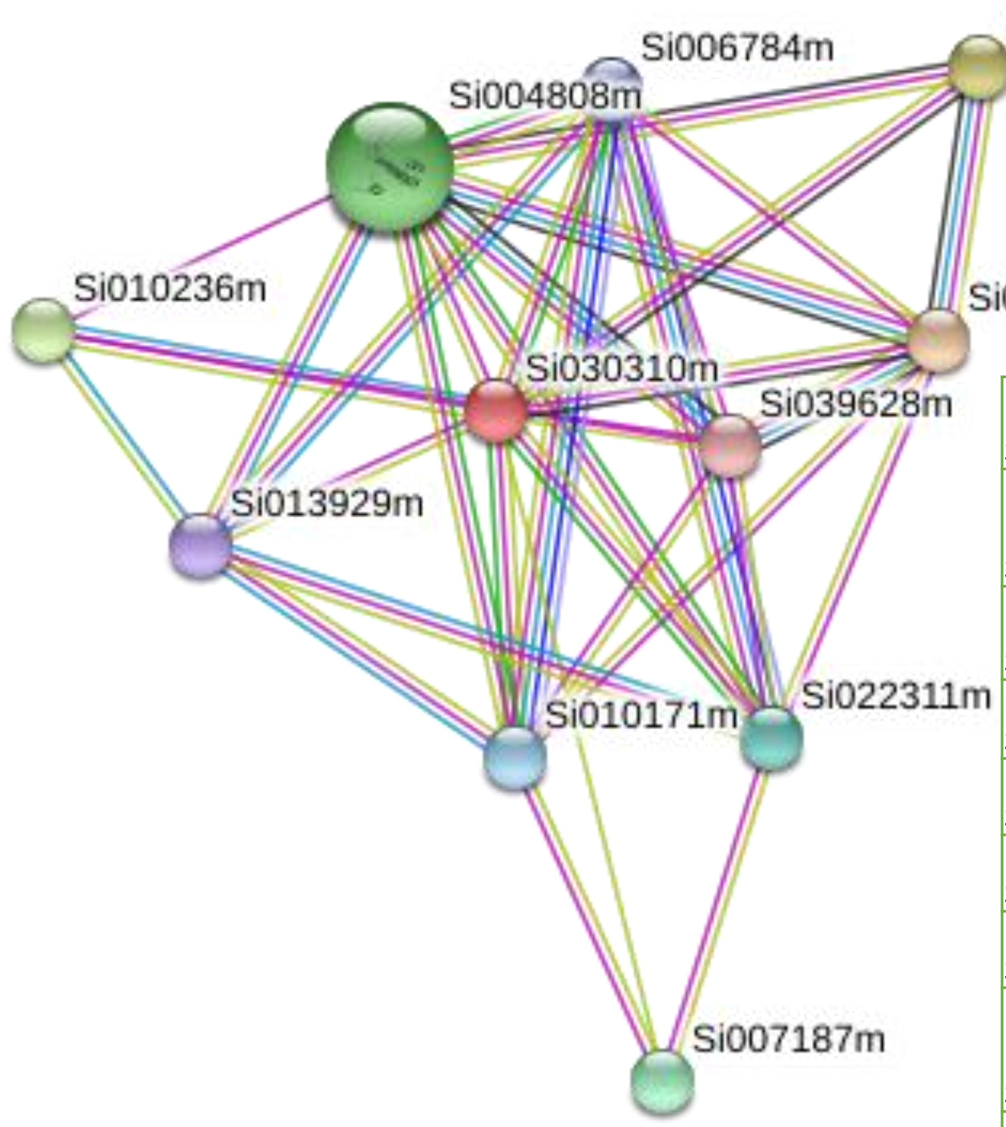

Seita.5G131900.1	Si000646m	SWI/SNF-RELATED CHROMATIN BINDING PROTEIN
Seita.7G319300.1	Si010236m	SET1/ASH2 HISTONE METHYLTRANSFERASE COMPLEX SUBUNIT ASH2
Seita.5G393100.1	Si004808m	SNARE protein YKT6, synaptobrevin/VAMP syperfamily
Seita.4G268200.1	Si007187m	AGAMOUS-LIKE MADS-BOX PROTEIN AGL16-RELATED
Seita.3G226100.1	Si022311m	HISTONE DEACETYLASE 8
Seita.7G281200.1	Si010171m	HISTONE DEACETYLASE 6
Seita.4G235100.1	Si006784m	HISTONE DEACETYLASE 11
Seita.6G072500.1	Si013929m	Transcriptional repressor EED/ESC/FIE, required for transcriptional silencing, WD repeat superfamily
Seita.2G170800.1	Si030310m	SiSET14

Supplementary Figure 8. *In silico* analysis of interacting patterns of SiSET14 proteins involved in regulation of histone methylation activities.

Table S1. Characteristic features of all the identified SET domain containing genes in *Setaria italica*

NIPGR ID	Transcript stable ID	Synonym Transcript stable ID	Subfamily cluster	Gene Name	Chromosome	Gene start (bp)	Gene end (bp)	Gene Length	Strand	Transcript count	Exon	Introns	Length (aa)	Mol. Wt (Da)	pI	negatively charged residues	positively charged residues	Instability index	stable or not	Aliphatic index	GRAVITY
SiSET01	Seita.1G107100.1	Si017644m	IV	ATXR5	1	9262162	9263521	1359	-1	1	4	3	471	51315.5	9.57	48	70	60.11	Unstable	67.03	-0.524
SiSET02	Seita.1G192400.1	Si016071m	II	ASHH2	1	27430644	27443589	12945	1	1	18	17	2115	228421	5.49	291	228	49.55	Unstable	64.63	-0.647
SiSET03	Seita.1G229500.1	Si016710m	II	ASHH3	1	30584818	30594029	9211	-1	1	11	10	595	65300.7	8.65	64	78	50.34	Unstable	68.57	-0.326
SiSET04	Seita.1G229600.1	-	-	-	1	30596645	30605280	8635	-1	1	12	11	667	75465.9	8.52	90	102	43.95	Unstable	68.41	-0.594
SiSET05	Seita.1G237500.1	Si019326m	V	SUVR2	1	31530443	31535915	5472	1	3	8	7	735	82443.8	6.56	98	93	46.97	Unstable	75.39	-0.449
	Seita.1G237500.2	-	-	-	1	31527957	31535915	7958	1	3	9	8	720	80602.6	6.47	97	91	46.75	Unstable	75.6	-0.471
	Seita.1G237500.3	-	-	-	1	31527957	31535915	7958	1	3	9	8	721	80730.7	6.56	97	92	46.7	Unstable	75.49	-0.476
SiSET06	Seita.1G255300.1	Si016370m	V	SUVH5	1	33041112	33043878	2766	-1	1	2	1	780	85095.6	5.81	105	89	42.05	Unstable	70.46	-0.482
SiSET07	Seita.1G291500.1	Si016095m	V	SUVR5	1	36020866	36033503	12637	1	2	12	11	1606	180216	6.07	210	181	49.23	Unstable	73.79	-0.54
	Seita.1G291500.2	-	-	-	1	36020866	36033503	12637	1	2	13	12	1574	176861	6.14	205	179	48.58	Unstable	73.49	-0.541
SiSET08	Seita.1G302100.1	Si016972m	IV	ribulose	1	36742261	36746281	4020	1	1	12	11	503	56580.8	9.05	56	64	50.24	Unstable	90	-0.28
SiSET09	Seita.1G310500.1	Si017054m	IV	SETD3	1	37260782	37264026	3244	1	1	5	4	485	52918	6.28	49	44	49.8	Unstable	98.25	0.019
SiSET10	Seita.2G084500.1	Si028938m	V	SUVH1	2	7396351	7403492	7141	1	2	3	2	825	89918.6	8.73	86	97	56.45	Unstable	67.25	-0.543
	Seita.2G084500.2	Si028939m	V	SUVH1	2	7396351	7403492	7141	1	2	3	2	825	89918.6	8.73	86	97	56.45	Unstable	67.25	-0.543
SiSET11	Seita.2G117200.1	Si029125m	V	SUVH9	2	11909398	11915032	5634	1	1	2	1	683	74638.7	5,88	80	72	53.32	Unstable	81.66	-0.235
SiSET12	Seita.2G148500.1	Si028793m	III	ATX2	2	19909341	19923798	14457	-1	4	27	26	1031	116276	6.89	142	139	49.39	Unstable	73.09	-0.543
	Seita.2G148500.2	-	-	-	2	19909305	19923803	14498	-1	4	25	24	1031	116276	6.89	142	139	49.39	Unstable	73.09	-0.543
	Seita.2G148500.3	-	-	-	2	19909333	19923798	14465	-1	4	24	23	954	107253	7.14	128	127	48.88	Unstable	73.57	-0.52
	Seita.2G148500.4	Si028792m	III	ATX2	2	19910221	19923799	13578	-1	4	25	24	1031	116276	6.89	142	139	49.39	Unstable	73.09	-0.543
SiSET13	Seita.2G158300.1	Si028884m	V	SUVH5	2	22840678	22844802	4124	1	1	2	1	883	95858.1	7.64	111	113	42.12	Unstable	64.9	-0.593
SiSET14	Seita.2G170800.1	Si030310m	II	ASHH3	2	25532347	25542617	10270	-1	2	13	12	354	40961.6	8.31	49	54	52.22	Unstable	63.53	-0.636
	Seita.2G170800.2	-	-	-	2	25532347	25542617	10270	-1	2	13	12	354	40961.6	8.31	49	54	52.22	Unstable	63.53	-0.636
SiSET15	Seita.2G185800.1	Si028728m	V	SUVH5	2	28151191	28156517	5326	1	2	4	3	1240	131156	6.47	151	144	46.28	Unstable	73.52	-0.476
	Seita.2G185800.2	-	-	-	2	28151176	28156517	5341	1	2	4	3	1189	126375	6.4	146	138	42.89	Unstable	74.12	-0.472
SiSET16	Seita.2G304400.1	Si028661m	III	ATX2	2	39720776	39733038	12262	1	2	23	22	1908	209170	9.01	177	241	50.93	Unstable	64.26	-0.572
	Seita.2G304400.2	Si028664m	III	ATX2	2	39720776	39733038	12262	1	2	21	20	1832	200400	8.97	168	226	51.37	Unstable	63.47	-0.575
SiSET17	Seita.2G310400.1	Si032678m	IV	SET40	2	40099131	40102791	3660	1	1	6	5	475	53439.8	5.64	57	47	40.29	Unstable	92.04	-0.154
SiSET18	Seita.2G411700.1	Si029068m	V	SUVR4	2	46973220	46978876	5656	1	1	8	7	742	82640.7	5.98	102	87	49.35	Unstable	64.02	-0.593
SiSET19	Seita.3G089500.1	Si021783m	II	SETD3	3	5778166	5783213	5047	-1	1	13	12	513	56850.1	5.63	69	61	54.62	Unstable	89.03	-0.251
SiSET20	Seita.3G126200.1	Si021853m	IV	ribulose	3	8565382	8570114	4732	-1	2	12	11	496	56703.2	5.11	74	54	58.68	Unstable	87.82	-0.314
	Seita.3G126200.2	-	-	-	3	8565284	8570114	4830	-1	2	11	10	430	49763.5	5.26	63	47	48.25	Unstable	92.6	-0.187
SiSET21	Seita.3G196800.1	Si021388m	V	SUVH1	3	14936981	14942246	5265	1	1	3	2	680	74858.1	7.91	77	80	38.38	stable	66.12	-0.472
SiSET22	Seita.3G259400.1	Si024266m	V	SUVH4	3	22556078	22562963	6885	1	1	13	12	533	59210.2	6.78	62	60	35.41	stable	80.81	-0.406
SiSET23	Seita.3G394700.1	Si021071m	II	-	3	49604211	49615657	11446	1	3	19	18	1158	129262	6.13	147	135	48.95	Unstable	78.39	-0.448
	Seita.3G394700.2	Si021082m	II	-	3	49604211	49612898	8687	1	3	16	15	1140	127306	6.06	145	132	49.09	Unstable	78.52	-0.449
	Seita.3G394700.3	-	-	-	3	49604211	49612898	8687	1	3	17	16	1158	129262	6.13	147	135	48.95	Unstable	78.39	-0.448
SiSET24	Seita.4G016800.1	Si006168m	II	-	4	1081906	1090385	8479	-1	1	16	15	620	69027.4	4.86	89	60	52.83	Unstable	76.92	-0.435
SiSET25	Seita.4G060900.1	Si005930m	I	EZ1	4	4506700	4517396	10696	1	1	16	15	956	10613.3	8.74	125	146	49.28	Unstable	64.92	-0.7
SiSET26	Seita.4G093300.1	Si005858m	V	SUVH1	4	7728963	7736499	7536	-1	3	4	3	831	91016.4	8.95	82	99	55.91	Unstable	69.13	-0.533
	Seita.4G093300.2	Si005857m	V	SUVH1	4	7728879	7735867	6988	-1	3	5	4	831	91016.4	8.95	82	99	55.91	Unstable	69.13	-0.533
	Seita.4G093300.3	-	-	-	4	7728879	7735867	6988	-1	3	5	4	831	91016.4	8.95	82	99	55.91	Unstable	69.13	-0.533
SiSET27	Seita.5G153000.1	Si000220m	III	ATX5	5	13713088	13721297	8209	-1	1	23	22	978	109496	7.72	129	133	55.52	Unstable	67.22	-0.487
SiSET28	Seita.5G248300.1	Si000146m	III	ATX4	5	30968342	30976926	8584	1	1	23	22	1054	120652	7.49	157	159	55.22	Unstable	66.49	-0.662
SiSET29	Seita.5G334500.1	Si002161m	V	SUVR3	5	38137587	38139979	2392	1	1	2	1	334	35173.1	8.51	32	38	57.38	Unstable	76.11	-0.091
SiSET30	Seita.5G355100.1	Si000438m	V	SUVH1	5	39622463	39627076	4613	1	1	3	2	737	80931	6.17	90	82	41.17	Unstable	65.4	-0.513
SiSET31	Seita.5G401600.1	Si001278m	-	SET3	5	42909782	42912324	2542	-1	1	5	4	476	53087.3	4.75	84	49	41.48	Unstable	88.93	-0.369
SiSET32	Seita.5G437600.1	Si000507m	V	SUVH4	5	45431532	45439754	8222	1	1	16	15	700	76973.6	8.53	85	94	51.66	Unstable	74.54	-0.551
SiSET33	Seita.5G464600.1	Si001848m	IV	ATXR5	5	46948560	46951871	3311	1	1	6	5	380	43075.3	8.94	52	63	70.49	Unstable	68.32	-0.624
SiSET34	Seita.5G470200.1	-	-	-	5	47197475	47202312	4837	1	1	15	14	466	52648.1	6.26	61	55	49.5	Unstable	88.13	-0.225
SiSET35	Seita.6G027900.1	-	-	-	6	2044542	2047270	2728	1	2	3	2	404	43040.9	4.44	63	29	52.52	Unstable	72.15	-0.278
	Seita.6G027900.2	-	-	-	6	2044542	2047294	2752	1	2	2	1	404	43040.9	4.44	63	29	52.52	Unstable	72.15	-0.278
SiSET36	Seita.6G042300.1	Si013113m	IV	ATXR3	6	3266110	3277765	11655	-1	1	20	19	2267	255545	6.53	340	322	54.44	Unstable	67.62	-0.802
SiSET37	Seita.6G093300.1	Si015622m	-	-	6	9103642	9111514	7872	-1	2	11	10	543	60368.9	4.93	75	49	36.29	stable	94.84	-0.106
	Seita.6G093300.2	-		-	6	9103642	9107294	3652	-1	2	10	9	509	56897.8	4.83	70	44	36.65	stable	94.26	-0.1
SiSET38	Seita.6G137100.1	Si015397m	V	-	6	24675305	24678068	2763	1	1	1	0	777	82110.2	8.71	91	102	45	Unstable	65.5	-0.526
SiSET39	Seita.6G137800.1	Si015003m	V	SUVH5	6	24819022	24822852	3830	1	1	2	1	1224	130811	8.41	137	147	41.95	Unstable	72.99	-0.394
SiSET40	Seita.6G164800.1	-	-	-	6	28758051	28766605	8554	1	1	12	11	454	50733.1	8.8	52	68	68.5	Unstable	61.87	-0.52
SiSET41	Seita.6G254300.1	Si013404m	V	SUVH9	6	35909936	35911855	1919	-1	1	1	0	639	70421.4	7.79	73	75	49.37	Unstable	77.95	-0.371
SiSET42	Seita.6G255000.1	Si015142m	V	SUVR4	6	35943462	35948342	4880	1	1	9	8	686	75368.7	5.82	89	74	46.47	Unstable	67.97	-0.46

SiSET43	Seita.7G097400.1	-	-	-	7	20005373	20011344	5971	-1	1	7	6	648	72317.8	5.78	76	60	48.49	Unstable	82.39	-0.071
SiSET44	Seita.7G098200.1	Si009854m	II	ASHH1	7	20122709	20128138	5429	1	1	10	9	535	60063.1	4.81	81	53	52.22	Unstable	74.64	-0.468
SiSET45	Seita.7G202500.1	Si012231m	V	SUVH6	7	27469561	27473548	3987	1	1	4	3	1126	121027	8.04	141	146	39.73	stable	75.58	-0.43
SiSET46	Seita.7G247900.1	Si010034m	IV	ATXR2	7	30627720	30632073	4353	-1	1	15	14	469	52035.5	4.73	72	42	59.07	Unstable	78	-0.272
SiSET47	Seita.7G309900.1	Si011948m	V	SUVH1	7	34630777	34633836	3059	-1	1	2	1	667	74503.7	7.72	82	84	53.13	Unstable	70.03	-0.537
SiSET48	Seita.8G004300.1	Si028261m	V	SUVH1	8	274071	276413	2342	1	1	1	0	664	74063	6.66	84	82	52.62	Unstable	69.77	-0.563
SiSET49	Seita.9G124100.1	Si035418m	IV	ASHR1	9	7774846	7779946	5100	1	3	14	13	485	54849	6.68	60	58	48.32	Unstable	88.1	-0.258
	Seita.9G124100.2	-	-	-	9	7769298	7779946	10648	1	3	14	13	386	43800.3	6.04	51	45	48.35	Unstable	91.99	-0.25
	Seita.9G124100.3	-	-	-	9	7774846	7779946	5100	1	3	14	13	386	43800.3	6.04	51	45	48.35	Unstable	91.99	-0.25
SiSET50	Seita.9G198400.1	Si039085m	IV	-	9	14178840	14184989	6149	1	1	15	14	789	87187.9	7.29	88	88	47.49	Unstable	93.14	-0.149
SiSET51	Seita.9G260300.1	Si036803m	IV	ATXR4	9	21436567	21440065	3498	-1	2	8	7	301	33502	5.79	40	32	67.2	Unstable	82.09	-0.206
	Seita.9G260300.2	Si036987m	IV	ATXR4	9	21436567	21440065	3498	-1	2	8	7	275	30801.9	5.82	38	31	66.25	Unstable	77.42	-0.262
SiSET52	Seita.9G429000.1	Si034160m	IV	ATXR4	9	48379130	48386420	7290	1	1	17	16	897	100203	8.4	121	131	51.33	Unstable	61.71	-0.745
SiSET53	Seita.9G525900.1	Si035066m	IV	-	9	55283355	55284980	1625	1	1	1	0	541	58725.9	6.03	81	75	39.27	stable	91.13	-0.189

- Not applicable

Table S2. Summary of domains present in SiSET proteins in *Setaria italica*.

Gene ID	PHD				SET				AWS				zf-CW				WIYLD				Pre-SET			
	From	To	E-Value	Bitscore	From	To	E-Value	Bitscore	From	To	E-Value	Bitscore	From	To	E-Value	Bitscore	From	To	E-Value	Bitscore	From	To	E-Value	Bitscore
SiSET01	146	191	5.30E-19	80.8179	348	459	3.21E-11	60.2047	-	-	-	-	-	-	-	-	-	-	-	-	-	-	-	-
SiSET02	-	-	-	-	1376	1496	1.57E-41	151.333	1325	1375	2.02E-15	73.5878	1226	1270	5.63E-12	63.6107	-	-	-	-	-	-	-	-
SiSET03	156	203	4.15E-06	44.7255	364	487	1.84E-44	154.415	322	362	2.61E-05	42.3866	-	-	-	-	-	-	-	-	-	-	-	-
SiSET04	57	104	0.00068	38.1771	264	385	2.87E-44	154.415	232	262	0.000748	38.1494	-	-	-	-	-	-	-	-	-	-	-	-
SiSET05	-	-	-	-	563	701	4.84E-26	104.339	-	-	-	-	-	-	-	-	23	75	1.62E-13	66.7191	402	554	8.24E-13	65.516
SiSET06	-	-	-	-	609	752	1.21E-32	123.213	-	-	-	-	-	-	-	-	-	-	-	-	504	601	1.08E-28	111.355
SiSET07	-	-	-	-	1451	1582	6.99E-35	131.688	-	-	-	-	-	-	-	-	-	-	-	-	1311	1443	1.47E-19	86.702
SiSET08	-	-	-	-	228	304	1.45E-10	58.6639	-	-	-	-	-	-	-	-	-	-	-	-	-	-	-	-
SiSET09	-	-	-	-	209	293	5.07E-06	44.7967	-	-	-	-	-	-	-	-	-	-	-	-	-	-	-	-
SiSET10	-	-	-	-	653	793	4.82E-33	124.754	-	-	-	-	-	-	-	-	-	-	-	-	545	645	1.14E-33	125.607
SiSET11	-	-	-	-	529	675	5.85E-24	98.1755	-	-	-	-	-	-	-	-	-	-	-	-	420	520	2.61E-29	112.51
SiSET12	573,629	618,749	48E-22.8.(	5865.206.	867	990	8.75E-35	130.532	-	-	-	-	-	-	-	-	-	-	-	-	-	-	-	-
SiSET13	-	-	-	-	712	854	1.19E-30	118.206	-	-	-	-	-	-	-	-	-	-	-	-	607	704	3.67E-29	113.281
SiSET14	-	-	-	-	125	241	3.37E-45	152.874	69	115	2.53E-10	55.4834	-	-	-	-	-	-	-	-	-	-	-	-
SiSET15	-	-	-	-	1069	1211	1.39E-28	113.198	-	-	-	-	-	-	-	-	-	-	-	-	964	1061	7.33E-35	130.23
SiSET16	14,431,585	14,871,677	000005868	1859.75.6	1763	1890	1.18E-37	140.162	-	-	-	-	-	-	-	-	-	-	-	-	-	-	-	-
SiSET17	-	-	-	-	174	269	0.005308	35.5519	-	-	-	-	-	-	-	-	-	-	-	-	-	-	-	-
SiSET18	-	-	-	-	572	711	5.33E-29	112.813	-	-	-	-	-	-	-	-	5	60	3.72E-12	62.8672	416	563	2.47E-18	81.6944
SiSET19	-	-	-	-	-	-	-	-	-	-	-	-	-	-	-	-	-	-	-	-	-	-	-	-
SiSET20	-	-	-	-	287	337	8.98E-11	59.0491	-	-	-	-	-	-	-	-	-	-	-	-	-	-	-	-
SiSET21	-	-	-	-	509	652	1.49E-31	119.747	-	-	-	-	-	-	-	-	-	-	-	-	400	500	5.58E-33	122.911
SiSET22	-	-	-	-	356	508	3.38E-29	111.657	-	-	-	-	-	-	-	-	-	-	-	-	232	347	1.65E-22	92.48
SiSET23	-	-	-	-	1019	1142	1.18E-42	153.259	-	-	-	-	-	-	-	-	-	-	-	-	-	-	-	-
SiSET24	-	-	-	-	167	250	4.5e-06 1	127.132	-	-	-	-	-	-	-	-	-	-	-	-	-	-	-	-
SiSET25	-	-	-	-	804	920	1.93E-36	134.769	-	-	-	-	-	-	-	-	-	-	-	-	-	-	-	-
SiSET26	-	-	-	-	661	799	1.06E-32	123.599	-	-	-	-	-	-	-	-	-	-	-	-	550	651	1.04E-29	114.436
SiSET27	366,605,550	415,716,596	7.08588E-5	.189.264.8	836	955	3.87E-36	133.999	-	-	-	-	-	-	-	-	-	-	-	-	-	-	-	-
SiSET28	625,680,436	671,791,485	19.6.43E-5	.7336.192	913.42.920	1031	3.75E-36	134.384	-	-	-	-	-	-	-	-	-	-	-	-	-	-	-	-
SiSET29	-	-	-	-	175	308	5.39E-29	108.961	-	-	-	-	-	-	-	-	-	-	-	-	50	167	4.56E-05	40.8633
SiSET30	-	-	-	-	566	711	3.51E-30	116.28	-	-	-	-	-	-	-	-	-	-	-	-	458	558	2.49E-32	121.37
SiSET31	-	-	-	-	174	200	3.87E-36	134.384	-	-	-	-	-	-	-	-	-	-	-	-	-	-	-	-
SiSET32	-	-	-	-	523	675	7.43E-29	112.043	-	-	-	-	-	-	-	-	-	-	-	-	412	515	1.75E-31	119.059
SiSET33	64	109	1.96E-16	72.7287	257	368	1.12E-10	57.8935	-	-	-	-	-	-	-	-	-	-	-	-	-	-	-	-
SiSET34	-	-	-	-	221	266	0.000894	37.8631	-	-	-	-	-	-	-	-	-	-	-	-	-	-	-	-
SiSET35	-	-	-	-	219	269	6.19E-08	50.4107	-	-	-	-	-	-	-	-	-	-	-	-	-	-	-	-
SiSET36	-	-	-	-	1716	1844	4.68E-24	100.872	-	-	-	-	-	-	-	-	-	-	-	-	-	-	-	-
SiSET37	-	-	-	-	167	220	2.00E-08	134.6	-	-	-	-	-	-	-	-	-	-	-	-	-	-	-	-
SiSET38	-	-	-	-	-	-	-	-	-	-	-	-	-	-	-	-	-	-	-	-	-	-	-	-
SiSET39	-	-	-	-	1050	1196	1.64E-29	115.509	-	-	-	-	-	-	-	-	-	-	-	-	945	1042	3.55E-31	119.829
SiSET40	-	-	-	-	315	433	6.64E-36	129.377	269	315	0.001914	36.2234	-	-	-	-	-	-	-	-	-	-	-	-
SiSET41	-	-	-	-	495	638	5.92E-25	100.872	-	-	-	-	-	-	-	-	-	-	-	-	387	486	1.01E-19	85.1612
SiSET42	-	-	-	-	515	653	2.75E-28	110.502	-	-	-	-	-	-	-	-	6	62	1.52E-14	69.4155	357	506	6.54E-18	80.1536
SiSET43	-	-	-	-	140	295	2.46E-06	46.3375	-	-	-	-	-	-	-	-	-	-	-	-	-	-	-	-
SiSET44	-	-	-	-	110	232	7.70E-42	146.711	58	109	7.74E-14	66.6542	-	-	-	-	-	-	-	-	-	-	-	-
SiSET45	-	-	-	-	955	1097	1.46E-30	118.591	-	-	-	-	-	-	-	-	-	-	-	-	850	947	6.34E-31	118.674
SiSET46	-	-	-	-	398	437	1.41E-11	61.3603	-	-	-	-	-	-	-	-	-	-	-	-	-	-	-	-
SiSET47	-	-	-	-	492	635	8.02E-31	117.435	-	-	-	-	-	-	-	-	-	-	-	-	383	483	1.55E-30	115.977
SiSET48	-	-	-	-	489	632	1.62E-29	113.969	-	-	-	-	-	-	-	-	-	-	-	-	380	480	3.47E-28	109.429
SiSET49	-	-	-	-	175	251	7.91E-12	62.1307	-	-	-	-	-	-	-	-	-	-	-	-	-	-	-	-
SiSET50	-	-	-	-	478	517	0.000211	40.5595	-	-	-	-	-	-	-	-	-	-	-	-	-	-	-	-
SiSET51	-	-	-	-	190	276	1.44E-09	54.2627	-	-	-	-	-	-	-	-	-	-	-	-	-	-	-	-
SiSET52	-	-	-	-	750	865	1.86E-36	134.769	-	-	-	-	-	-	-	-	-	-	-	-	-	-	-	-
SiSET53	-	-	-	-	311	363	2.32E-11	60.9751	-	-	-	-	-	-	-	-	-	-	-	-	-	-	-	-

Gene ID	SAD_SRA				Rubis-subs-bind				zf-HC5HC2H_2				PWWP				FYRC				FYRN			
	From	To	E-Value	Bitscore	From	To	E-Value	Bitscore	From	To	E-Value	Bitscore	From	To	E-Value	Bitscore	From	To	E-Value	Bitscore	From	To	E-Value	Bitscore
SiSET01	-	-	-	-	-	-	-	-	-	-	-	-	-	-	-	-	-	-	-	-	-	-	-	-
SiSET02	-	-	-	-	-	-	-	-	-	-	-	-	-	-	-	-	-	-	-	-	-	-	-	-
SiSET03	-	-	-	-	-	-	-	-	-	-	-	-	-	-	-	-	-	-	-	-	-	-	-	-
SiSET04	-	-	-	-	-	-	-	-	-	-	-	-	-	-	-	-	-	-	-	-	-	-	-	-
SiSET05	-	-	-	-	-	-	-	-	-	-	-	-	-	-	-	-	-	-	-	-	-	-	-	-
SiSET06	335	481	9.29E-27	107.724	-	-	-	-	-	-	-	-	-	-	-	-	-	-	-	-	-	-	-	-
SiSET07	-	-	-	-	-	-	-	-	-	-	-	-	-	-	-	-	-	-	-	-	-	-	-	-
SiSET08	-	-	-	-	335	470	1.16E-14	70.8357	-	-	-	-	-	-	-	-	-	-	-	-	-	-	-	-
SiSET09	-	-	-	-	343	461	1.79E-13	66.9837	-	-	-	-	-	-	-	-	-	-	-	-	-	-	-	-
SiSET10	369	518	9.13E-74	239.077	-	-	-	-	-	-	-	-	-	-	-	-	-	-	-	-	-	-	-	-
SiSET11	235	390	4.28E-82	258.722	-	-	-	-	-	-	-	-	-	-	-	-	-	-	-	-	-	-	-	-
SiSET12	-	-	-	-	-	-	-	-	-	-	-	-	270	355	3.16E-18	81.5517	473	549	1.13E-16	76.9655	413	466	1.72E-15	72.625
SiSET13	437	583	2.33E-60	202.098	-	-	-	-	-	-	-	-	-	-	-	-	-	-	-	-	-	-	-	-
SiSET14	-	-	-	-	-	-	-	-	-	-	-	-	-	-	-	-	-	-	-	-	-	-	-	-
SiSET15	784	940	4.36E-66	220.972	-	-	-	-	-	-	-	-	-	-	-	-	-	-	-	-	-	-	-	-
SiSET16	-	-	-	-	-	-	-	-	-	-	-	-	-	-	-	-	-	-	-	-	-	-	-	-
SiSET17	-	-	-	-	287	384	0.000343	39.2493	-	-	-	-	-	-	-	-	-	-	-	-	-	-	-	-
SiSET18	-	-	-	-	-	-	-	-	-	-	-	-	-	-	-	-	-	-	-	-	-	-	-	-
SiSET19	-	-	-	-	359	483	1.16E-31	118.215	-	-	-	-	-	-	-	-	-	-	-	-	-	-	-	-
SiSET20	-	-	-	-	414	480	1.55E-07	49.6497	-	-	-	-	-	-	-	-	-	-	-	-	-	-	-	-
SiSET21	219	373	2.44E-78	248.707	-	-	-	-	-	-	-	-	-	-	-	-	-	-	-	-	-	-	-	-
SiSET22	41	203	2.57E-66	213.654	-	-	-	-	-	-	-	-	-	-	-	-	-	-	-	-	-	-	-	-
SiSET23	-	-	-	-	-	-	-	-	-	-	-	-	-	-	-	-	-	-	-	-	-	-	-	-
SiSET24	-	-	-	-	-	-	-	-	-	-	-	-	-	-	-	-	-	-	-	-	-	-	-	-
SiSET25	-	-	-	-	-	-	-	-	-	-	-	-	-	-	-	-	-	-	-	-	-	-	-	-
SiSET26	374	523	8.46E-72	233.684	-	-	-	-	-	-	-	-	-	-	-	-	-	-	-	-	-	-	-	-
SiSET27	-	-	-	-	-	-	-	-	-	-	-	-	177	279	5.02E-34	127.048	-	-	-	-	-	-	-	-
SiSET28	-	-	-	-	-	-	-	-	-	-	-	-	246	352	7.65E-26	103.936	-	-	-	-	-	-	-	-
SiSET29	-	-	-	-	-	-	-	-	-	-	-	-	-	-	-	-	-	-	-	-	-	-	-	-
SiSET30	277	431	2.52E-75	241.773	-	-	-	-	-	-	-	-	-	-	-	-	-	-	-	-	-	-	-	-
SiSET31	-	-	-	-	-	-	-	-	-	-	-	-	-	-	-	-	-	-	-	-	-	-	-	-
SiSET32	221	383	1.43E-66	217.891	-	-	-	-	-	-	-	-	-	-	-	-	-	-	-	-	-	-	-	-
SiSET33	-	-	-	-	-	-	-	-	-	-	-	-	-	-	-	-	-	-	-	-	-	-	-	-
SiSET34	-	-	-	-	-	-	-	-	-	-	-	-	-	-	-	-	-	-	-	-	-	-	-	-
SiSET35	-	-	-	-	-	-	-	-	-	-	-	-	-	-	-	-	-	-	-	-	-	-	-	-
SiSET36	-	-	-	-	-	-	-	-	-	-	-	-	-	-	-	-	-	-	-	-	-	-	-	-
SiSET37	-	-	-	-	27	133	0.000294	39.6345	-	-	-	-	-	-	-	-	-	-	-	-	-	-	-	-
SiSET38	610	761	3.66E-63	209.416	-	-	-	-	-	-	-	-	-	-	-	-	-	-	-	-	-	-	-	-
SiSET39	764	921	3.97E-60	204.024	-	-	-	-	-	-	-	-	-	-	-	-	-	-	-	-	-	-	-	-
SiSET40	-	-	-	-	-	-	-	-	-	-	-	-	-	-	-	-	-	-	-	-	-	-	-	-
SiSET41	199	356	1.61E-68	221.743	-	-	-	-	-	-	-	-	-	-	-	-	-	-	-	-	-	-	-	-
SiSET42	-	-	-	-	-	-	-	-	-	-	-	-	-	-	-	-	-	-	-	-	-	-	-	-
SiSET43	-	-	-	-	-	-	-	-	-	-	-	-	-	-	-	-	-	-	-	-	-	-	-	-
SiSET44	-	-	-	-	-	-	-	-	-	-	-	-	-	-	-	-	-	-	-	-	-	-	-	-
SiSET45	-	-	-	-	-	-	-	-	-	-	-	-	-	-	-	-	-	-	-	-	-	-	-	-
SiSET46	-	-	-	-	-	-	-	-	-	-	-	-	-	-	-	-	-	-	-	-	-	-	-	-
SiSET47	203	356	1.37E-81	257.181	-	-	-	-	-	-	-	-	-	-	-	-	-	-	-	-	-	-	-	-
SiSET48	200	353	1.58E-82	259.492	-	-	-	-	-	-	-	-	-	-	-	-	-	-	-	-	-	-	-	-
SiSET49	-	-	-	-	-	-	-	-	-	-	-	-	-	-	-	-	-	-	-	-	-	-	-	-
SiSET50	-	-	-	-	-	-	-	-	-	-	-	-	-	-	-	-	-	-	-	-	-	-	-	-
SiSET51	-	-	-	-	-	-	-	-	-	-	-	-	-	-	-	-	-	-	-	-	-	-	-	-
SiSET52	-	-	-	-	-	-	-	-	-	-	-	-	-	-	-	-	-	-	-	-	-	-	-	-
SiSET53	-	-	-	-	-	-	-	-	-	-	-	-	-	-	-	-	-	-	-	-	-	-	-	-

Gene ID	PostSET				SAND				GYF				DUF1777				TPR				SANT			
	From	To	E-Value	Bitscore	From	To	E-Value	Bitscore	From	To	E-Value	Bitscore	From	To	E-Value	Bitscore	From	To	E-Value	Bitscore	From	To	E-Value	Bitscore
SiSET01	-	-	-	-	-	-	-	-	-	-	-	-	-	-	-	-	-	-	-	-	-	-	-	-
SiSET02	-	-	-	-	-	-	-	-	-	-	-	-	-	-	-	-	-	-	-	-	-	-	-	-
SiSET03	-	-	-	-	-	-	-	-	-	-	-	-	-	-	-	-	-	-	-	-	-	-	-	-
SiSET04	-	-	-	-	-	-	-	-	-	-	-	-	-	-	-	-	-	-	-	-	-	-	-	-
SiSET05	-	-	-	-	-	-	-	-	-	-	-	-	-	-	-	-	-	-	-	-	-	-	-	-
SiSET06	-	-	-	-	-	-	-	-	-	-	-	-	-	-	-	-	-	-	-	-	-	-	-	-
SiSET07	-	-	-	-	-	-	-	-	-	-	-	-	-	-	-	-	-	-	-	-	-	-	-	-
SiSET08	-	-	-	-	-	-	-	-	-	-	-	-	-	-	-	-	-	-	-	-	-	-	-	-
SiSET09	-	-	-	-	-	-	-	-	-	-	-	-	-	-	-	-	-	-	-	-	-	-	-	-
SiSET10	-	-	-	-	-	-	-	-	-	-	-	-	-	-	-	-	-	-	-	-	-	-	-	-
SiSET11	-	-	-	-	-	-	-	-	-	-	-	-	-	-	-	-	-	-	-	-	-	-	-	-
SiSET12	-	-	-	-	-	-	-	-	-	-	-	-	-	-	-	-	-	-	-	-	-	-	-	-
SiSET13	-	-	-	-	-	-	-	-	-	-	-	-	-	-	-	-	-	-	-	-	-	-	-	-
SiSET14	-	-	-	-	-	-	-	-	-	-	-	-	-	-	-	-	-	-	-	-	-	-	-	-
SiSET15	-	-	-	-	-	-	-	-	-	-	-	-	-	-	-	-	-	-	-	-	-	-	-	-
SiSET16	-	-	-	-	-	-	-	-	-	-	-	-	-	-	-	-	-	-	-	-	-	-	-	-
SiSET17	-	-	-	-	-	-	-	-	-	-	-	-	-	-	-	-	-	-	-	-	-	-	-	-
SiSET18	-	-	-	-	-	-	-	-	-	-	-	-	-	-	-	-	-	-	-	-	-	-	-	-
SiSET19	-	-	-	-	-	-	-	-	-	-	-	-	-	-	-	-	-	-	-	-	-	-	-	-
SiSET20	-	-	-	-	-	-	-	-	-	-	-	-	-	-	-	-	-	-	-	-	-	-	-	-
SiSET21	-	-	-	-	-	-	-	-	-	-	-	-	-	-	-	-	-	-	-	-	-	-	-	-
SiSET22	-	-	-	-	-	-	-	-	-	-	-	-	-	-	-	-	-	-	-	-	-	-	-	-
SiSET23	1142	1158	0.00227	37.3848	-	-	-	-	-	-	-	-	-	-	-	-	-	-	-	-	-	-	-	-
SiSET24	-	-	-	-	-	-	-	-	-	-	-	-	-	-	-	-	-	-	-	-	-	-	-	-
SiSET25	-	-	-	-	-	-	-	-	-	-	-	-	-	-	-	-	-	-	-	-	-	-	-	-
SiSET26	-	-	-	-	-	-	-	-	-	-	-	-	-	-	-	-	-	-	-	-	-	-	-	-
SiSET27	-	-	-	-	-	-	-	-	-	-	-	-	-	-	-	-	-	-	-	-	-	-	-	-
SiSET28	-	-	-	-	514	585	0.00519	36.5292	-	-	-	-	-	-	-	-	-	-	-	-	-	-	-	-
SiSET29	-	-	-	-	-	-	-	-	-	-	-	-	-	-	-	-	-	-	-	-	-	-	-	-
SiSET30	-	-	-	-	-	-	-	-	-	-	-	-	-	-	-	-	-	-	-	-	-	-	-	-
SiSET31	-	-	-	-	-	-	-	-	-	-	-	-	-	-	-	-	-	-	-	-	-	-	-	-
SiSET32	-	-	-	-	-	-	-	-	-	-	-	-	-	-	-	-	-	-	-	-	-	-	-	-
SiSET33	-	-	-	-	-	-	-	-	-	-	-	-	-	-	-	-	-	-	-	-	-	-	-	-
SiSET34	-	-	-	-	-	-	-	-	-	-	-	-	-	-	-	-	-	-	-	-	-	-	-	-
SiSET35	-	-	-	-	-	-	-	-	-	-	-	-	-	-	-	-	-	-	-	-	-	-	-	-
SiSET36	-	-	-	-	-	-	-	-	896	949	0.00064	40.0364	-	-	-	-	-	-	-	-	-	-	-	-
SiSET37	-	-	-	-	-	-	-	-	-	-	-	-	-	-	-	-	-	-	-	-	-	-	-	-
SiSET38	-	-	-	-	-	-	-	-	-	-	-	-	-	-	-	-	-	-	-	-	-	-	-	-
SiSET39	-	-	-	-	-	-	-	-	-	-	-	-	-	-	-	-	-	-	-	-	-	-	-	-
SiSET40	-	-	-	-	-	-	-	-	-	-	-	-	-	-	-	-	-	-	-	-	-	-	-	-
SiSET41	-	-	-	-	-	-	-	-	-	-	-	-	-	-	-	-	-	-	-	-	-	-	-	-
SiSET42	-	-	-	-	-	-	-	-	-	-	-	-	-	-	-	-	-	-	-	-	-	-	-	-
SiSET43	-	-	-	-	-	-	-	-	-	-	-	-	-	-	-	-	-	-	-	-	-	-	-	-
SiSET44	-	-	-	-	-	-	-	-	-	-	-	-	-	-	-	-	-	-	-	-	-	-	-	-
SiSET45	-	-	-	-	-	-	-	-	-	-	-	-	-	-	-	-	-	-	-	-	-	-	-	-
SiSET46	-	-	-	-	-	-	-	-	-	-	-	-	-	-	-	-	-	-	-	-	-	-	-	-
SiSET47	-	-	-	-	-	-	-	-	-	-	-	-	-	-	-	-	-	-	-	-	-	-	-	-
SiSET48	-	-	-	-	-	-	-	-	-	-	-	-	-	-	-	-	-	-	-	-	-	-	-	-
SiSET49	-	-	-	-	-	-	-	-	-	-	-	-	-	-	-	-	-	-	-	-	-	-	-	-
SiSET50	-	-	-	-	-	-	-	-	-	-	-	-	-	-	-	-	-	-	-	-	-	-	-	-
SiSET51	-	-	-	-	-	-	-	-	-	-	-	-	-	-	-	-	-	-	-	-	-	-	-	-
SiSET52	-	-	-	-	-	-	-	-	-	-	-	-	-	-	-	-	-	-	-	-	-	-	-	-
SiSET53	-	-	-	-	-	-	-	-	-	-	-	-	-	-	-	-	-	-	-	-	-	-	-	-

- Not present

Table S3. The Ka/Ks ratios and estimated divergence time for orthologous *SiSET* genes between foxtail millet, sorghum, maize, rice and Brachypodium

NIPGR ID	Phytozome ID	Location on foxtail millet genome			Location on sorghum genome				Ka	Ks	Ka/Ks	Mya
		Chromosome	Start	End	Gene ID	Chromosome	Start	End				
SiSET01	Seita.1G107100	1	9262162	9263521	Sb04g001900	4	1628407	1630029	0.05	0.35	0.13	26.7
SiSET02	Seita.1G192400	1	27431267	27443199								
SiSET03	Seita.1G229500	1	30584825	30593856								
SiSET04	Seita.1G229600	1	30597788	30603387								
SiSET05	Seita.1G237500	1	31530485	31535057								
SiSET06	Seita.1G255300	1	33041260	33043602	Sb04g033260	4	63161476	63163839	0.09	0.25	0.38	19
SiSET07	Seita.1G291500	1	36022715	36033504								
SiSET08	Seita.1G302100	1	36742251	36746753	Sb04g029430	4	59511683	59517146	0.05	0.29	0.18	22
SiSET09	Seita.1G310500	1	37260864	37263996	Sb04g028760	4	58880576	58883937	0.07	0.61	0.12	47.2
SiSET10	Seita.2G084500	2	7396424	7403493	Sb02g006620	2	8258007	8260499	0.04	0.27	0.16	21.1
SiSET11	Seita.2G117200	2	11909548	11915017	Sb02g010210	2	15131527	15133659	0.08	0.35	0.24	26.8
SiSET12	Seita.2G148500	2	19909313	19923580								
SiSET13	Seita.2G158300	2	22841147	22843798	Sb06g024160	6	53283608	53286522	0.08	0.48	0.18	36.6
SiSET14	Seita.2G170800	2	25532388	25541393	Sb02g020844	2	51264959	51275647	0.08	0.28	0.28	21.3
SiSET15	Seita.2G185800	2	28151597	28156452								
SiSET16	Seita.2G304400	2	30278262	30280450	Sb02g024510	2	58875740	58877915	0.09	0.19	0.44	15
SiSET17	Seita.2G310400	2	39722445	39733003	Sb02g032470	2	67238207	67249886	0.06	0.53	0.12	40.9
SiSET18	Seita.2G411700	2	40099240	40102634	Sb02g032970	2	67554650	67557393	0.09	0.25	0.38	19
SiSET19	Seita.3G089500	2	46973422	46978473								
SiSET20	Seita.3G126200	3	5778304	5783126	Sb03g029140	3	57269351	57274240	0.07	0.28	0.24	21.7
SiSET22	Seita.3G259400	3	14937095	14942247	Sb09g024010	9	53598830	53603859	0.0514	0.2368	4.607	26.7
SiSET23	Seita.3G394700	3	22555774	22562876	Sb09g019060	9	47612684	47628090	0.1946	0.463	2.3792	15
SiSET24	Seita.4G016800	3	49605515	49612901	Sb08g002530	8	2603260	2608547	0.1193	0.2566	2.1509	40.9
SiSET25	Seita.4G060900	4	1082524	1090272	Sb10g001630	10	1307612	1314321	0.063	0.2515	3.9921	33.2
SiSET26	Seita.4G093300	4	4508310	4517397	Sb10g004560	10	4043340	4052683	0.0441	0.298	0.2	54.6
SiSET27	Seita.5G153000	4	7728850	7732975	Sb06g001340	6	1901343	1906385	0.0898	0.243	2.706	19
SiSET28	Seita.5G248300	5	13713202	13720765	Sb03g001640	3	1471409	1479596	0.0638	0.3124	4.8966	21.7
SiSET29	Seita.5G334500	5	30969211	30976908	Sb03g029850	3	58089330	58098201	0.09	0.32	0.27	24.5
SiSET30	Seita.5G355100	5	38137796	38139908	Sb03g035910	3	63927803	63930072	0.03	0.19	0.15	14.3
SiSET31	Seita.5G401600	5	39622569	39627037	Sb03g037660	3	65611172	65614571	0.03	0.41	0.07	31.8
SiSET32	Seita.5G437600	5	42910037	42912248	Sb03g041640	3	69124118	69126453	0.07	0.3	0.24	23.2
SiSET34	Seita.5G470200	5	46948448	46951251	Sb03g046950	3	73968225	73973385	0.05	0.17	0.2	13.4
SiSET35	Seita.6G027900	6	3266616	3277553	Sb07g005020	7	6711948	6722970	0.05	0.27	0.16	21.1
SiSET36	Seita.6G042300	6	9104290	9107203	Sb07g007800	7	12611024	12615538	0.04	0.19	0.2	14.6
SiSET37	Seita.6G093300	6	24675305	24677638								
SiSET38	Seita.6G137100	6	24819022	24822852	Sb07g019860	7	51110076	51113858	0.0638	0.3124	0.2	17
SiSET40	Seita.6G164800	6	35909936	35911855	Sb07g023560	7	58442307	58444606	0.104	0.4472	4.3	8
SiSET41	Seita.6G254300	6	35945072	35948105	Sb07g023480	7	58390246	58394033	0.04	0.51	0.07	39.1
SiSET42	Seita.6G255000	7	20002888	20010656								
SiSET43	Seita.7G097400	7	20122709	20128474	Sb06g016720	6	45788727	45801754	0.06	0.32	0.18	24.5
SiSET44	Seita.7G098200	7	27469561	27473548	Sb02g022936	2	56218440	56222818	0.09	0.23	0.2	18
SiSET45	Seita.7G202500	7	30627990	30631961	Sb06g029440	6	58057750	58061990	0.09	0.19	0.2	15
SiSET46	Seita.7G247900	7	34631824	34633773	Sb08g001570	8	1525584	1527770	0.05	0.21	0.22	16
SiSET48	Seita.8G004300	9	7774932	7779473	Sb01g010740	1	9651114	9656511	0.07	0.71	0.1	54.6
SiSET49	Seita.9G124100	9	14179183	14184721								
SiSET50	Seita.9G198400	9	21436977	21439968	Sb01g022220	1	27302727	27309159	0.06	0.44	0.14	33.8
SiSET51	Seita.9G260300	9	48379173	48386403	Sb01g037450	1	61009255	61017926	0.05	0.29	0.18	22
SiSET52	Seita.9G429000	9	55283355	55285198	Sb01g045950	1	69023068	69024699	0.11	0.46	0.23	35.7
					**Mean**				**0.1**	**0.3**	**0.8**	**25.8**

NIPGR ID	Location on maize genome				Ka	Ks	Ka/Ks	Mya
	Gene ID	Chromosome	Start	End				
SiSET01	GRMZM2G305124	5	71873816	71875082	0.05	0.36	0.14	27.4
SiSET02	GRMZM2G352431	5	172608483	172622165	0.04	0.19	0.19	14.7
SiSET03								
SiSET04								
SiSET05								
SiSET06								
SiSET07	GRMZM2G172427	4	160952067	160967258	0.07	0.28	0.26	21.2
SiSET08	GRMZM2G077258	4	163422412	163428013	0.04	0.34	0.12	26.2
SiSET09	GRMZM2G063316	4	167002541	167008338	0.03	0.22	0.15	17.1
SiSET10	AC233961.1_FG001	7	20255014	20257584	0.08	0.32	0.25	25
SiSET11	GRMZM2G025924	2	170175284	170177778	0.03	0.35	0.07	26.7
SiSET12	GRMZM2G013794	7	72261177	72305728	0.03	0.34	0.08	26.1
SiSET13	GRMZM2G021044	2	21907392	21910615	0.11	0.48	0.22	36.6
SiSET14	GRMZM2G033694	2	174419091	174436402	0.08	0.42	0.2	32.5
SiSET15								
SiSET16	GRMZM2G418752	7	110942240	110947021	0.05	0.51	0.1	39.3
SiSET17	GRMZM2G409224	2	198751071	198769406	0.03	0.35	0.09	27.1
SiSET18	GRMZM2G092131	7	144638342	144640953	0.05	0.28	0.18	21.4
SiSET19								
SiSET20	GRMZM2G047695	3	215608644	215618038	0.06	0.26	0.22	19.9
SiSET22	GRMZM2G140577	8	119356309	119359897	0.06	0.37	0.16	28.4
SiSET23								
SiSET24								
SiSET25	GRMZM2G078458	6	62715442	62725933	0.08	0.32	0.25	25
SiSET26	GRMZM2G157820	6	79622840	79633054	0.08	0.7	0.12	54.2
SiSET27								
SiSET28	GRMZM2G085266	3	3200792	3209093	0.03	0.34	0.08	26.1
SiSET29								
SiSET30	GRMZM2G105869	2	200042816	200045662	0.07	0.51	0.15	38.9
SiSET31	GRMZM2G139710	3	187087134	187091766	0.05	0.33	0.17	25.6
SiSET32	GRMZM2G092759	8	164816458	164819112	0.06	0.29	0.2	22.2
SiSET34	GRMZM2G149587	8	156446973	156452761	0.06	0.32	0.18	24.3
SiSET35	GRMZM2G149153	4	29377433	29389813	0.07	0.51	0.15	38.9
SiSET36	GRMZM2G045507	10	71171840	71175862	0.08	0.7	0.12	54.2
SiSET37								
SiSET38								
SiSET40	GRMZM2G165011	1	201882383	201884831	0.08	0.32	0.25	25
SiSET41	GRMZM2G042442	4	48369758	48374526	0.03	0.34	0.08	26.1
SiSET42								
SiSET43	GRMZM2G147619	2	52449235	52456233	0.11	0.48	0.22	36.6
SiSET44	GRMZM2G300955	2	177370536	177375918	0.06	0.32	0.18	24.3
SiSET45	GRMZM2G164277	2	7527529	7531928	0.07	0.28	0.26	21.2
SiSET46	GRMZM2G416913	3	103829944	103832043	0.04	0.34	0.12	26.2
SiSET48	GRMZM2G457881	5	11957842	11966150	0.07	0.37	0.18	28.4
SiSET49								
SiSET50	GRMZM2G080462	1	242199385	242208360	0.03	0.22	0.15	17.1
SiSET51	GRMZM5G875502	9	135509551	135524285	0.06	0.59	0.1	28
SiSET52	GRMZM2G091916	1	14508652	14510556	0.07	0.51	0.15	20
	**Mean**				**0.1**	**0.4**	**0.2**	**28.0**

NIPGR ID	Location on rice genome				Ka	Ks	Ka/Ks	Mya
	Gene ID	Chromosome	Start	End				
SiSET01	BGIOSGA007416	2	1286895	1288511	0.24	0.43	0.56	33.1
SiSET02	BGIOSGA008417	2	22529712	22543057	0.27	0.46	0.59	35.4
SiSET03	BGIOSGA006064	2	25747074	25750016	0.24	0.44	0.55	33.8
SiSET04	BGIOSGA006064	2	25747074	25750016	0.29	0.45	0.64	34.6
SiSET05								
SiSET06								
SiSET07								
SiSET08	BGIOSGA008960	2	32061481	32065411	0.26	0.44	0.59	33.8
SiSET09	BGIOSGA008995	2	32587087	32589551	0.26	0.45	0.58	34.6
SiSET10	BGIOSGA033726	11	19010451	19012960	0.26	0.46	0.57	35.4
SiSET11	BGIOSGA025596	7	13136942	13138996	0.3	0.44	0.68	33.8
SiSET12	BGIOSGA030183	9	2360602	2373289	0.26	0.45	0.58	34.6
SiSET13								
SiSET14	BGIOSGA030025	9	7235438	7243022	0.28	0.43	0.65	33.1
SiSET15	BGIOSGA030599	9	10287688	10291220	0.25	0.44	0.57	33.8
SiSET16	BGIOSGA029775	9	13143768	13149315	0.29	0.5	0.58	38.5
SiSET17								38.5
SiSET18								38.5
SiSET19								37.7
SiSET20	BGIOSGA037202	12	7058654	7063034	0.26	0.44	0.59	33.8
SiSET22	BGIOSGA017817	5	25578582	25581593	0.26	0.5	0.52	38.5
SiSET23								
SiSET24								
SiSET25								
SiSET26								
SiSET27								
SiSET28	BGIOSGA002119	1	6978520	6985370	0.31	0.43	0.72	33.1
SiSET29	BGIOSGA004136	1	29778919	29786122	0.25	0.57	0.44	43.8
SiSET30	BGIOSGA000735	1	35889673	35891546	0.27	0.46	0.59	35.4
SiSET31	BGIOSGA004668	1	37901959	37904379	0.24	0.57	0.42	43.8
SiSET32	BGIOSGA000391	1	41777904	41780121	0.28	0.57	0.49	43.8
SiSET34	BGIOSGA005241	1	46620372	46624865	0.29	0.45	0.64	34.6
SiSET35	BGIOSGA028096	8	5051653	5062046	0.24	0.57	0.42	43.8
SiSET36								
SiSET37								
SiSET38								
SiSET40	BGIOSGA026463	8	30282425	30284212	0.26	0.51	0.51	39.2
SiSET41								
SiSET42	BGIOSGA015103	4	18884918	18888248	0.25	0.48	0.52	36.9
SiSET43	BGIOSGA016424	4	19164169	19168200	0.29	0.47	0.62	36.2
SiSET44	BGIOSGA030599	9	10287688	10291220	0.25	0.47	0.53	36.2
SiSET45	BGIOSGA014297	4	31019317	31023486	0.27	0.46	0.59	35.4
SiSET46	BGIOSGA036697	12	1692467	1694867	0.27	0.44	0.61	33.8
SiSET48	BGIOSGA009959	3	32453480	32457017	0.26	0.44	0.59	33.8
SiSET49								
SiSET50								
SiSET51	BGIOSGA010882	3	12085476	12091234	0.3	0.5	0.6	38.5
SiSET52	BGIOSGA011375	3	4157638	4159248	0.26	0.44	0.59	33.8
	**Mean**				**0.3**	**0.5**	**0.6**	**36.5**

NIPGR ID	Location on Brachypodium genome				Ka	Ks	Ka/Ks	Mya
	Gene ID	Chromosome	Start	End				
SiSET01	BRADI3G02220	3	1315188	1316926	0.63	0.26	0.4	45
SiSET02								
SiSET03								
SiSET04	BRADI3G48497	3	49909565	49920047	0.87	0.31	0.4	62.1
SiSET05								
SiSET06								
SiSET07								
SiSET08	BRADI3G56450	3	56324346	56329668	0.72	0.34	0.5	51.4
SiSET09	BRADI3G56840	3	56589292	56592387	0.83	0.26	0.3	59.3
SiSET10	BRADI1G53840	1	52249194	52252832	0.87	0.31	0.4	62.1
SiSET11	BRADI1G29010	1	24486501	24492916	0.71	0.28	0.4	50.7
SiSET12	BRADI4G08510	4	7575866	7591276	0.83	0.26	0.3	59.3
SiSET13								
SiSET14								
SiSET15								
SiSET16	BRADI4G30040	4	35755180	35757472	0.29	0.5	0.58	38.5
SiSET17								
SiSET18	BRADI1G28267	1	23475647	23482592	0.72	0.34	0.5	51.4
SiSET19								
SiSET20	BRADI4G39580	4	44225050	44231664	0.87	0.31	0.4	62.1
SiSET22	BRADI4G25940	4	31262136	31264998	0.83	0.26	0.3	59.3
SiSET23								
SiSET24								
SiSET25	BRADI1G49580	1	48265788	48273118	0.83	0.26	0.3	59.3
SiSET26	BRADI1G48340	1	46969765	46980112	0.83	0.26	0.3	59.3
SiSET27	BRADI1G53840	1	52249194	52252832	0.63	0.26	0.4	45
SiSET28	BRADI2G07082	2	5416613	5424796	0.83	0.26	0.3	59.3
SiSET29	BRADI2G45430	2	45798809	45807250	0.87	0.31	0.4	62.1
SiSET30	BRADI2G51320	2	50939680	50942008	0.71	0.28	0.4	50.7
SiSET31								
SiSET32	BRADI2G56792	2	55044577	55047013	0.28	0.57	0.49	43.8
SiSET34	BRADI2G61717	2	58693840	58699114	0.29	0.45	0.64	34.6
SiSET35	BRADI3G15410	3	13731787	13742725	0.24	0.57	0.42	43.8
SiSET36	BRADI3G18940	3	17507100	17513675	0.3	0.55	0.55	42.3
SiSET37								
SiSET38								
SiSET40								
SiSET41								
SiSET42								
SiSET43	BRADI5G10110	5	13367495	13371977	0.29	0.47	0.62	36.2
SiSET44								
SiSET45	BRADI5G22730	5	24977637	24982157	0.27	0.46	0.59	35.4
SiSET46	BRADI4G43717	4	47330463	47332493	0.27	0.44	0.61	33.8
SiSET48	BRADI1G11010	1	8002529	8007898	0.26	0.44	0.59	62.1
SiSET49	BRADI3G30360	3	32451734	32456945	0.26	0.48	0.54	36.9
SiSET50								
SiSET51								
SiSET52	BRADI1G73790	1	71189050	71190929	0.26	0.44	0.59	62.1
	**Mean**				**0.6**	**0.4**	**0.5**	**50.7**

Table S4. Characteristics of the promoter region of *SiSET* genes in *Setaria italica*

Cis-acting Element	SiSET01	SiSET02	SiSET03	SiSET05	SiSET06	SiSET07	SiSET09	SiSET10	SiSET11	SiSET12	SiSET13	SiSET14	SiSET15	SiSET16	SiSET17	SiSET18	SiSET19	SiSET20	Si002161m	Si006168m	Si012231m	Si010034m	Si005930m	Si015003m	Si000146m	Si011948m	Si000438m	Si013113m	Si009854m	Si015622m	Si000220m	Si009324m	Si005858m	Si013404m	Si000507m	Si015238m	Si001278m	Si015142m	Si001848	Si019056m	Si021082m	Si016971m	Si021388m	Si036987m	Si035418m	Si024266m	Si028261m	Si034160m	Si035066m	Si029660m	Si039085m
-10PEHVPSBD	414 (-),	-	1106 (-), 806 (-),	1032 (-),	736 (+),	-	1118 (+), 1249 (+), 258 (-), 431 (-)	743 (+),	-	229 (+),	-	-	-	-	-	-	896 (-),	1317 (+), 655 (+),	549 (+), 757 (-),	-	211 (+), 30 (+),	1048 (+),	811 (+),	231 (+),	-	-	1226 (-),	-	587 (+),	-	265 (+),	-	112 (+),	-	396 (-), 402 (-),	496 (-),	341 (+),	1152 (+), 375 (-), 905 (+)	-	-	-	-	-	1440 (-),	-	224 (+),	49 (-),	77 (-),	107 (+), 299 (+),	-	-
2SSEEDPROTBANAPA	-	-	-	-	-	-	104 (-), 669 (+)	952 (-),	-	1109 (+),	-	-	-	1175 (-), 1447 (-)	842 (+),	395 (-),	-	-	-	-	-	-	-	-	-	-	-	-	253 (+), 534 (-), 910	1423 (-), 285 (-)	552 (-),	1203 (-),	-	1101 (+), 113 (-), 162	-	-	-	-	-	-	22 (+), 40 (+)	46 (+), 674 (-)	958 (-),	-	-	-	-	-	-	-	-
-300CORE	-	-	-	-	-	1371 (+),	697 (-),	-	-	-	-	-	-	-	-	-	-	-	-	-	-	-	-	260 (-),	-	-	-	-	-	-	-	-	-	-	-	1113 (+),	-	-	-	273 (-),	-	-	-	876 (+),	119 (-), 669 (+)	-	-	-	-	-	-
-300ELEMENT	-	903 (-),	1195 (+),	-	1057 (+), 713 (+),	1103 (+), 1415 (+),	395 (-), 696 (-), 924 (+),	1139 (+), 5 (+), 620 (+)	744 (-),	1293 (+),	436 (-), 487 (-),	-	1483 (-),	-	71 (-), 798 (-),	1013 (+),	511 (-), 984 (+),	27 (-),	289 (-), 390 (+),	-	1010 (-),	-	-	259 (-),	-	-	-	-	1442 (-),	305 (+),	-	-	-	-	-	1113 (+), 1277 (+), 270 (+)	1225 (+),	172 (-),	1348 (+), 4 (+),	1006 (+), 272 (-), 319 (+)	83 (+),	-	1135 (+), 1298 (+), 450 (+)	1019 (-), 876 (+),	-	-	-	-	-	-	136 (+), 99 (+),
-300MOTIFZMZEIN	-	-	-	-	-	-	-	-	-	-	-	-	-	-	-	997 (-)	-	-	-	-	-	-	-	-	-	-	-	-	-	-	-	-	-	-	-	-	-	-	-	-	-	-	-	-	-	-	-	-	-	-	-
5659BOXLELAT5659	-	-	-	-	-	-	-	-	-	-	-	-	-	-	-	-	-	-	-	-	-	-	-	-	-	-	-	-	-	-	-	-	-	-	-	711 (-)	-	-	-	-	-	-	-	-	-	-	-	-	-	-	-
AACACOREOSGLUB1	-	402 (-),	-	638 (-),	889 (-), 893 (-), 903 (-), 907 (-), 917 (-), 989 (+)	-	615 (-),	954 (-),	-	-	-	-	-	-	-	-	-	-	-	-	-	-	-	-	252 (+),	1014 (-),	-	930 (+),	-	1475 (-),	-	-	-	572 (+),	-	-	-	-	1037 (-),	-	-	-	-	1045 (-), 583 (+),	-	-	-	-	-	-	-
ABREATCONSENSUS	-	-	-	-	-	-	-	82 (-)	-	-	-	-	-	-	916 (-)	-	1125 (+)	-	-	-	1116 (+)	-	-	1075 (-)	-	-	-	-	-	-	-	-	-	-	-	-	-	-	-	-	-	-	-	-	-	-	-	-	-	-	-
ABREATRD22	-	-	-	-	-	-	-	-	-	-	-	-	-	-	-	-	-	-	-	-	-	-	-	1074 (-)	-	-	-	-	-	-	-	-	-	-	-	-	-	-	-	-	-	-	-	-	-	-	-	-	-	-	-
ABREAZMRAB28	-	-	-	-	-	-	-	82 (+)	-	-	-	-	-	-	-	-	-	-	-	-	-	-	-	-	-	-	-	-	-	-	-	-	-	-	-	-	-	-	-	-	-	-	-	-	-	-	-	-	-	-	-
ABREBZMRAB28	-	-	1429 (+)	-	-	-	-	-	-	-	-	-	-	-	-	1040 (-)	-	-	-	-	-	-	-	-	-	-	-	-	-	-	-	-	-	-	-	-	-	-	-	-	-	-	-	-	-	-	-	-	-	-	-
ABRECE1HVA22	-	-	-	-	-	-	-	-	-	-	-	-	-	-	-	-	-	-	-	332 (-)	-	-	-	-	-	-	-	-	-	-	-	-	-	-	-	-	-	-	-	-	-	-	-	-	-	-	-	-	-	-	-
ABRELATERD1	749 (-),	130 (-), 131 (+), 222 (+), 279 (+), 423 (-), 708 (-),	1168 (+), 1412 (-), 1431 (-), 88 (+),	-	940 (-),	114 (-), 16 (-), 506 (+), 725 (-),	-	1246 (+), 1346 (-), 1347 (+), 430 (-), 66 (-), 84 (-), 85 (+),	-	787 (-),	-	-	1071 (+), 227 (+), 337 (+), 768 (+),	374 (-), 375 (+),	409 (-), 410 (+), 918 (-), 919 (+),	1043 (+), 507 (+),	1126 (+), 701 (-),	486 (-), 613 (-),	1373 (-), 1374 (+),	141 (+), 502 (-), 576 (+), 668 [illegible]	1096 (-), 1116 (-), 1117 (+), [illegible]	1329 (+), 35 (-), 36 (+), 992 [illegible]	936 (-),	1077 (-), 1078 (+), 757 (+), [illegible]	-	607 (+),	1082 (-), 157 (-), 158 (+), 179 [illegible]	-	476 (-), 477 (+),	-	213 (-), 214 (+), 235 (-), 236 (+), 28 [illegible]	38 (-),	700 (-),	1068 (+), 793 (+), 852 (-), [illegible]	117 (-), 118 (+), 528 (-),	864 (-), 865 (+),	1170 (-), 1171 (+),	-	128 (-),	211 (-), 892 (+),	512 (-),	610 (-), 744 (-),	1026 (-), 12 (+),	68 (+), 966 (-),	-	1018 (-), 1019 (+), 1048 (+), [illegible]	935 (+),	1051 (-), 167 (-), 168 (+),	1161 (+), 282 (-),	1104 (+),	1428 (-), 517 (+),
ABREMOTIFAOSOSEM	-	-	-	-	-	-	-	-	-	-	-	-	-	-	-	[illegible]	[illegible]	[illegible]	[illegible]	[illegible]	[illegible]	[illegible]	[illegible]	[illegible]	[illegible]	[illegible]	[illegible]	[illegible]	[illegible]	[illegible]	[illegible]	[illegible]	[illegible]	[illegible]	[illegible]	[illegible]	[illegible]	[illegible]	[illegible]	[illegible]	[illegible]	[illegible]	[illegible]	[illegible]	[illegible]	[illegible]	[illegible]	[illegible]	[illegible]	[illegible]	[illegible]
ABREMOTIFIIIOSRAB16B	-	-	-	-	15 (-)	-	-	-	-	-	-	-	-	-	-	[illegible]	[illegible]	[illegible]	[illegible]	[illegible]	[illegible]	[illegible]	[illegible]	[illegible]	[illegible]	[illegible]	[illegible]	[illegible]	[illegible]	[illegible]	[illegible]	[illegible]	[illegible]	[illegible]	[illegible]	[illegible]	[illegible]	[illegible]	[illegible]	[illegible]	[illegible]	[illegible]	[illegible]	[illegible]	[illegible]	[illegible]	[illegible]	[illegible]	[illegible]	[illegible]	[illegible]
ABREOSRAB21	-	-	-	-	-	1348 (-),	-	1246 (+), 1343 (-), 179 (-), 81 (-), 85 (+)	-	784 (-),	-	-	768 (+),	-	410 (+), 915 (-),	[illegible]	[illegible]	[illegible]	[illegible]	[illegible]	[illegible]	[illegible]	[illegible]	[illegible]	[illegible]	[illegible]	[illegible]	[illegible]	[illegible]	[illegible]	[illegible]	[illegible]	[illegible]	[illegible]	[illegible]	[illegible]	[illegible]	[illegible]	[illegible]	[illegible]	[illegible]	[illegible]	[illegible]	[illegible]	[illegible]	[illegible]	[illegible]	[illegible]	[illegible]	[illegible]	[illegible]
ABRERATCAL	-	1117 (+), 1246 (+), 129 (-), 422 (-), 565 (-),	1167 (+),	-	17 (+),	724 (-),	-	1328 (+), 1345 (-), 1346 (+), 65 (-), 83 (-), 84 (+),	-	-	-	103 (-),	1362 (-), 1449 (-), 336 (+), 767 (+),	373 (-), 374 (+),	0	[illegible]	[illegible]	[illegible]	[illegible]	[illegible]	[illegible]	[illegible]	[illegible]	[illegible]	[illegible]	[illegible]	[illegible]	[illegible]	[illegible]	[illegible]	[illegible]	[illegible]	[illegible]	[illegible]	[illegible]	[illegible]	[illegible]	[illegible]	[illegible]	[illegible]	[illegible]	[illegible]	[illegible]	[illegible]	[illegible]	[illegible]	[illegible]	[illegible]	[illegible]	[illegible]	[illegible]
ABREZMRAB28	-	-	-	-	-	-	-	83 (+), 83 (-)	-	-	-	-	-	-	-	[illegible]	[illegible]	[illegible]	[illegible]	[illegible]	[illegible]	[illegible]	[illegible]	[illegible]	[illegible]	[illegible]	[illegible]	[illegible]	[illegible]	[illegible]	[illegible]	[illegible]	[illegible]	[illegible]	[illegible]	[illegible]	[illegible]	[illegible]	[illegible]	[illegible]	[illegible]	[illegible]	[illegible]	[illegible]	[illegible]	[illegible]	[illegible]	[illegible]	[illegible]	[illegible]	[illegible]
ACGTABOX	-	-	[illegible]	[illegible]	[illegible]	[illegible]	[illegible]	[illegible]	[illegible]	[illegible]	[illegible]	[illegible]	[illegible]	[illegible]	[illegible]	[illegible]	[illegible]	[illegible]	[illegible]	[illegible]	[illegible]	[illegible]	[illegible]	[illegible]	[illegible]	[illegible]	[illegible]	[illegible]	[illegible]	[illegible]	[illegible]	[illegible]	[illegible]	[illegible]	[illegible]	[illegible]	[illegible]	[illegible]	[illegible]	[illegible]	[illegible]	[illegible]	[illegible]	[illegible]	[illegible]	[illegible]	[illegible]	[illegible]	[illegible]	[illegible]	[illegible]
ACGTABREMOTIFA2OSEM	-	279 (+),	[illegible]	[illegible]	[illegible]	[illegible]	[illegible]	[illegible]	[illegible]	[illegible]	[illegible]	[illegible]	[illegible]	[illegible]	[illegible]	[illegible]	[illegible]	[illegible]	[illegible]	[illegible]	[illegible]	[illegible]	[illegible]	[illegible]	[illegible]	[illegible]	[illegible]	[illegible]	[illegible]	[illegible]	[illegible]	[illegible]	[illegible]	[illegible]	[illegible]	[illegible]	[illegible]	[illegible]	[illegible]	[illegible]	[illegible]	[illegible]	[illegible]	[illegible]	[illegible]	[illegible]	[illegible]	[illegible]	[illegible]	[illegible]	[illegible]
ACGTABREMOTIFAOSOSEM	[illegible]	[illegible]	[illegible]	[illegible]	[illegible]	[illegible]	[illegible]	[illegible]	[illegible]	[illegible]	[illegible]	[illegible]	[illegible]	[illegible]	[illegible]	[illegible]	[illegible]	[illegible]	[illegible]	[illegible]	[illegible]	[illegible]	[illegible]	[illegible]	[illegible]	[illegible]	[illegible]	[illegible]	[illegible]	[illegible]	[illegible]	[illegible]	[illegible]	[illegible]	[illegible]	[illegible]	[illegible]	[illegible]	[illegible]	[illegible]	[illegible]	[illegible]	[illegible]	[illegible]	[illegible]	[illegible]	[illegible]	[illegible]	[illegible]	[illegible]	[illegible]
ACGTATERD1	1370 (+), 1370 (-), 247 (+), 247 (-), 544 (+), 544 (-), 750 (+), 750 (-),	131 (+), 131 (-), 222 (+), 222 (-), 227 (+), 227 (-), 279 (+), 279 (-), 424 (+), 424 (-), 709 (+), 709 (-),	[illegible]	[illegible]	[illegible]	[illegible]	[illegible]	[illegible]	[illegible]	[illegible]	[illegible]	[illegible]	[illegible]	[illegible]	[illegible]	[illegible]	[illegible]	[illegible]	[illegible]	[illegible]	[illegible]	[illegible]	[illegible]	[illegible]	[illegible]	[illegible]	[illegible]	[illegible]	[illegible]	[illegible]	[illegible]	[illegible]	[illegible]	[illegible]	[illegible]	[illegible]	[illegible]	[illegible]	[illegible]	[illegible]	[illegible]	[illegible]	[illegible]	[illegible]	[illegible]	[illegible]	[illegible]	[illegible]	[illegible]	[illegible]	[illegible]
ACGTCBOX	1369 (+), 1369 (-), 246 (+), 246 (-)	226 (+), 226 (-),	[illegible]	[illegible]	[illegible]	[illegible]	[illegible]	[illegible]	[illegible]	[illegible]	[illegible]	[illegible]	[illegible]	[illegible]	[illegible]	[illegible]	[illegible]	[illegible]	[illegible]	[illegible]	[illegible]	[illegible]	[illegible]	[illegible]	[illegible]	[illegible]	[illegible]	[illegible]	[illegible]	[illegible]	[illegible]	[illegible]	[illegible]	[illegible]	[illegible]	[illegible]	[illegible]	[illegible]	[illegible]	[illegible]	[illegible]	[illegible]	[illegible]	[illegible]	[illegible]	[illegible]	[illegible]	[illegible]	[illegible]	[illegible]	[illegible]
ACGTOSGLUB1	-	-	[illegible]	[illegible]	[illegible]	[illegible]	[illegible]	[illegible]	[illegible]	[illegible]	[illegible]	[illegible]	[illegible]	[illegible]	[illegible]	[illegible]	[illegible]	[illegible]	[illegible]	[illegible]	[illegible]	[illegible]	[illegible]	[illegible]	[illegible]	[illegible]	[illegible]	[illegible]	[illegible]	[illegible]	[illegible]	[illegible]	[illegible]	[illegible]	[illegible]	[illegible]	[illegible]	[illegible]	[illegible]	[illegible]	[illegible]	[illegible]	[illegible]	[illegible]	[illegible]	[illegible]	[illegible]	[illegible]	[illegible]	[illegible]	[illegible]
ACGTTBOX	-	-	[illegible]	[illegible]	[illegible]	[illegible]	[illegible]	[illegible]	[illegible]	[illegible]	[illegible]	[illegible]	[illegible]	[illegible]	[illegible]	[illegible]	[illegible]	[illegible]	[illegible]	[illegible]	[illegible]	[illegible]	[illegible]	[illegible]	[illegible]	[illegible]	[illegible]	[illegible]	[illegible]	[illegible]	[illegible]	[illegible]	[illegible]	[illegible]	[illegible]	[illegible]	[illegible]	[illegible]	[illegible]	[illegible]	[illegible]	[illegible]	[illegible]	[illegible]	[illegible]	[illegible]	[illegible]	[illegible]	[illegible]	[illegible]	[illegible]
ACIPVPAL2	-	-	[illegible]	[illegible]	[illegible]	[illegible]	[illegible]	[illegible]	[illegible]	[illegible]	[illegible]	[illegible]	[illegible]	[illegible]	[illegible]	[illegible]	[illegible]	[illegible]	[illegible]	[illegible]	[illegible]	[illegible]	[illegible]	[illegible]	[illegible]	[illegible]	[illegible]	[illegible]	[illegible]	[illegible]	[illegible]	[illegible]	[illegible]	[illegible]	[illegible]	[illegible]	[illegible]	[illegible]	[illegible]	[illegible]	[illegible]	[illegible]	[illegible]	[illegible]	[illegible]	[illegible]	[illegible]	[illegible]	[illegible]	[illegible]	[illegible]
AGCBOXNPGLB	-	1158 (+),	[illegible]	[illegible]	[illegible]	[illegible]	[illegible]	[illegible]	[illegible]	[illegible]	[illegible]	[illegible]	[illegible]	[illegible]	[illegible]	[illegible]	[illegible]	[illegible]	[illegible]	[illegible]	[illegible]	[illegible]	[illegible]	[illegible]	[illegible]	[illegible]	[illegible]	[illegible]	[illegible]	[illegible]	[illegible]	[illegible]	[illegible]	[illegible]	[illegible]	[illegible]	[illegible]	[illegible]	[illegible]	[illegible]	[illegible]	[illegible]	[illegible]	[illegible]	[illegible]	[illegible]	[illegible]	[illegible]	[illegible]	[illegible]	[illegible]
AGMOTIFNTMYB2	-	-	[illegible]	[illegible]	[illegible]	[illegible]	[illegible]	[illegible]	[illegible]	[illegible]	[illegible]	[illegible]	[illegible]	[illegible]	[illegible]	[illegible]	[illegible]	[illegible]	[illegible]	[illegible]	[illegible]	[illegible]	[illegible]	[illegible]	[illegible]	[illegible]	[illegible]	[illegible]	[illegible]	[illegible]	[illegible]	[illegible]	[illegible]	[illegible]	[illegible]	[illegible]	[illegible]	[illegible]	[illegible]	[illegible]	[illegible]	[illegible]	[illegible]	[illegible]	[illegible]	[illegible]	[illegible]	[illegible]	[illegible]	[illegible]	[illegible]
AMMORESIIUDCRNIA1	-	-	[illegible]	[illegible]	[illegible]	[illegible]	[illegible]	[illegible]	[illegible]	[illegible]	[illegible]	[illegible]	[illegible]	[illegible]	[illegible]	[illegible]	[illegible]	[illegible]	[illegible]	[illegible]	[illegible]	[illegible]	[illegible]	[illegible]	[illegible]	[illegible]	[illegible]	[illegible]	[illegible]	[illegible]	[illegible]	[illegible]	[illegible]	[illegible]	[illegible]	[illegible]	[illegible]	[illegible]	[illegible]	[illegible]	[illegible]	[illegible]	[illegible]	[illegible]	[illegible]	[illegible]	[illegible]	[illegible]	[illegible]	[illegible]	[illegible]
AMMORESIVDCRNIA1	-	-	[illegible]	[illegible]	[illegible]	[illegible]	[illegible]	[illegible]	[illegible]	[illegible]	[illegible]	[illegible]	[illegible]	[illegible]	[illegible]	[illegible]	[illegible]	[illegible]	[illegible]	[illegible]	[illegible]	[illegible]	[illegible]	[illegible]	[illegible]	[illegible]	[illegible]	[illegible]	[illegible]	[illegible]	[illegible]	[illegible]	[illegible]	[illegible]	[illegible]	[illegible]	[illegible]	[illegible]	[illegible]	[illegible]	[illegible]	[illegible]	[illegible]	[illegible]	[illegible]	[illegible]	[illegible]	[illegible]	[illegible]	[illegible]	[illegible]
AMMORESVDCRNIA1	-	-	[illegible]	[illegible]	[illegible]	[illegible]	[illegible]	[illegible]	[illegible]	[illegible]	[illegible]	[illegible]	[illegible]	[illegible]	[illegible]	[illegible]	[illegible]	[illegible]	[illegible]	[illegible]	[illegible]	[illegible]	[illegible]	[illegible]	[illegible]	[illegible]	[illegible]	[illegible]	[illegible]	[illegible]	[illegible]	[illegible]	[illegible]	[illegible]	[illegible]	[illegible]	[illegible]	[illegible]	[illegible]	[illegible]	[illegible]	[illegible]	[illegible]	[illegible]	[illegible]	[illegible]	[illegible]	[illegible]	[illegible]	[illegible]	[illegible]
AMYBOX1	561 (+),	-	[illegible]	[illegible]	[illegible]	[illegible]	[illegible]	[illegible]	[illegible]	[illegible]	[illegible]	[illegible]	[illegible]	[illegible]	[illegible]	[illegible]	[illegible]	[illegible]	[illegible]	[illegible]	[illegible]	[illegible]	[illegible]	[illegible]	[illegible]	[illegible]	[illegible]	[illegible]	[illegible]	[illegible]	[illegible]	[illegible]	[illegible]	[illegible]	[illegible]	[illegible]	[illegible]	[illegible]	[illegible]	[illegible]	[illegible]	[illegible]	[illegible]	[illegible]	[illegible]	[illegible]	[illegible]	[illegible]	[illegible]	[illegible]	[illegible]
AMYBOX2	925 (-),	-	[illegible]	[illegible]	[illegible]	[illegible]	[illegible]	[illegible]	[illegible]	[illegible]	[illegible]	[illegible]	[illegible]	[illegible]	[illegible]	[illegible]	[illegible]	[illegible]	[illegible]	[illegible]	[illegible]	[illegible]	[illegible]	[illegible]	[illegible]	[illegible]	[illegible]	[illegible]	[illegible]	[illegible]	[illegible]	[illegible]	[illegible]	[illegible]	[illegible]	[illegible]	[illegible]	[illegible]	[illegible]	[illegible]	[illegible]	[illegible]	[illegible]	[illegible]	[illegible]	[illegible]	[illegible]	[illegible]	[illegible]	[illegible]	[illegible]
ANAERO1CONSENSUS	-	403 (-), 805 (-), 825 (+), 938 (+),	[illegible]	[illegible]	[illegible]	[illegible]	[illegible]	[illegible]	[illegible]	[illegible]	[illegible]	[illegible]	[illegible]	[illegible]	[illegible]	[illegible]	[illegible]	[illegible]	[illegible]	[illegible]	[illegible]	[illegible]	[illegible]	[illegible]	[illegible]	[illegible]	[illegible]	[illegible]	[illegible]	[illegible]	[illegible]	[illegible]	[illegible]	[illegible]	[illegible]	[illegible]	[illegible]	[illegible]	[illegible]	[illegible]	[illegible]	[illegible]	[illegible]	[illegible]	[illegible]	[illegible]	[illegible]	[illegible]	[illegible]	[illegible]	[illegible]
ANAERO2CONSENSUS	197 (-), 28 (+), 291 (+), 612 (+), 965 [illegible]	-	[illegible]	[illegible]	[illegible]	[illegible]	[illegible]	[illegible]	[illegible]	[illegible]	[illegible]	[illegible]	[illegible]	[illegible]	[illegible]	[illegible]	[illegible]	[illegible]	[illegible]	[illegible]	[illegible]	[illegible]	[illegible]	[illegible]	[illegible]	[illegible]	[illegible]	[illegible]	[illegible]	[illegible]	[illegible]	[illegible]	[illegible]	[illegible]	[illegible]	[illegible]	[illegible]	[illegible]	[illegible]	[illegible]	[illegible]	[illegible]	[illegible]	[illegible]	[illegible]	[illegible]	[illegible]	[illegible]	[illegible]	[illegible]	[illegible]
ANAERO3CONSENSUS	813 (-)	133 (-)	[illegible]	[illegible]	[illegible]	[illegible]	[illegible]	[illegible]	[illegible]	[illegible]	[illegible]	[illegible]	[illegible]	[illegible]	[illegible]	[illegible]	[illegible]	[illegible]	[illegible]	[illegible]	[illegible]	[illegible]	[illegible]	[illegible]	[illegible]	[illegible]	[illegible]	[illegible]	[illegible]	[illegible]	[illegible]	[illegible]	[illegible]	[illegible]	[illegible]	[illegible]	[illegible]	[illegible]	[illegible]	[illegible]	[illegible]	[illegible]	[illegible]	[illegible]	[illegible]	[illegible]	[illegible]	[illegible]	[illegible]	[illegible]	[illegible]
ANAERO4CONSENSUS	-	-	[illegible]	[illegible]	[illegible]	[illegible]	[illegible]	[illegible]	[illegible]	[illegible]	[illegible]	[illegible]	[illegible]	[illegible]	[illegible]	[illegible]	[illegible]	[illegible]	[illegible]	[illegible]	[illegible]	[illegible]	[illegible]	[illegible]	[illegible]	[illegible]	[illegible]	[illegible]	[illegible]	[illegible]	[illegible]	[illegible]	[illegible]	[illegible]	[illegible]	[illegible]	[illegible]	[illegible]	[illegible]	[illegible]	[illegible]	[illegible]	[illegible]	[illegible]	[illegible]	[illegible]	[illegible]	[illegible]	[illegible]	[illegible]	[illegible]
ARE1	-	-	[illegible]	[illegible]	[illegible]	[illegible]	[illegible]	[illegible]	[illegible]	[illegible]	[illegible]	[illegible]	[illegible]	[illegible]	[illegible]	[illegible]	[illegible]	[illegible]	[illegible]	[illegible]	[illegible]	[illegible]	[illegible]	[illegible]	[illegible]	[illegible]	[illegible]	[illegible]	[illegible]	[illegible]	[illegible]	[illegible]	[illegible]	[illegible]	[illegible]	[illegible]	[illegible]	[illegible]	[illegible]	[illegible]	[illegible]	[illegible]	[illegible]	[illegible]	[illegible]	[illegible]	[illegible]	[illegible]	[illegible]	[illegible]	[illegible]
ARECOREZMGAPC4	-	-	[illegible]	[illegible]	[illegible]	[illegible]	[illegible]	[illegible]	[illegible]	[illegible]	[illegible]	[illegible]	[illegible]	[illegible]	[illegible]	[illegible]	[illegible]	[illegible]	[illegible]	[illegible]	[illegible]	[illegible]	[illegible]	[illegible]	[illegible]	[illegible]	[illegible]	[illegible]	[illegible]	[illegible]	[illegible]	[illegible]	[illegible]	[illegible]	[illegible]	[illegible]	[illegible]	[illegible]	[illegible]	[illegible]	[illegible]	[illegible]	[illegible]	[illegible]	[illegible]	[illegible]	[illegible]	[illegible]	[illegible]	[illegible]	[illegible]
ARFAT	-	1267 (+),	[illegible]	[illegible]	[illegible]	[illegible]	[illegible]	[illegible]	[illegible]	[illegible]	[illegible]	[illegible]	[illegible]	[illegible]	[illegible]	[illegible]	[illegible]	[illegible]	[illegible]	[illegible]	[illegible]	[illegible]	[illegible]	[illegible]	[illegible]	[illegible]	[illegible]	[illegible]	[illegible]	[illegible]	[illegible]	[illegible]	[illegible]	[illegible]	[illegible]	[illegible]	[illegible]	[illegible]	[illegible]	[illegible]	[illegible]	[illegible]	[illegible]	[illegible]	[illegible]	[illegible]	[illegible]	[illegible]	[illegible]	[illegible]	[illegible]
ARR1AT	1088 (-), 1196 (+), 1305 (-), 1395 (-), 170 (+), 327 (+), 336 (+), 557 (+), 569 (+), 699 (+), 990 (+),	1005 (+), 1333 (+), 47 (+), 633 (-), 784 (-), 801 (+), 844 (+),	[illegible]	[illegible]	[illegible]	[illegible]	[illegible]	[illegible]	[illegible]	[illegible]	[illegible]	[illegible]	[illegible]	[illegible]	[illegible]	[illegible]	[illegible]	[illegible]	[illegible]	[illegible]	[illegible]	[illegible]	[illegible]	[illegible]	[illegible]	[illegible]	[illegible]	[illegible]	[illegible]	[illegible]	[illegible]	[illegible]	[illegible]	[illegible]	[illegible]	[illegible]	[illegible]	[illegible]	[illegible]	[illegible]	[illegible]	[illegible]	[illegible]	[illegible]	[illegible]	[illegible]	[illegible]	[illegible]	[illegible]	[illegible]	[illegible]
ASF1MOTIFCAMV	1368 (+), 324 (+), 751 (-), 817 (+),	145 (+), 217 (+), 225 (+),	[illegible]	[illegible]	[illegible]	[illegible]	[illegible]	[illegible]	[illegible]	[illegible]	[illegible]	[illegible]	[illegible]	[illegible]	[illegible]	[illegible]	[illegible]	[illegible]	[illegible]	[illegible]	[illegible]	[illegible]	[illegible]	[illegible]	[illegible]	[illegible]	[illegible]	[illegible]	[illegible]	[illegible]	[illegible]	[illegible]	[illegible]	[illegible]	[illegible]	[illegible]	[illegible]	[illegible]	[illegible]	[illegible]	[illegible]	[illegible]	[illegible]	[illegible]	[illegible]	[illegible]	[illegible]	[illegible]	[illegible]	[illegible]	[illegible]
ATHB6COREAT	-	-	[illegible]	[illegible]	[illegible]	[illegible]	[illegible]	[illegible]	[illegible]	[illegible]	[illegible]	[illegible]	[illegible]	[illegible]	[illegible]	[illegible]	[illegible]	[illegible]	[illegible]	[illegible]	[illegible]	[illegible]	[illegible]	[illegible]	[illegible]	[illegible]	[illegible]	[illegible]	[illegible]	[illegible]	[illegible]	[illegible]	[illegible]	[illegible]	[illegible]	[illegible]	[illegible]	[illegible]	[illegible]	[illegible]	[illegible]	[illegible]	[illegible]	[illegible]	[illegible]	[illegible]	[illegible]	[illegible]	[illegible]	[illegible]	[illegible]
AUXRETGA1GMGH3	-	-	[illegible]	[illegible]	[illegible]	[illegible]	[illegible]	[illegible]	[illegible]	[illegible]	[illegible]	[illegible]	[illegible]	[illegible]	[illegible]	[illegible]	[illegible]	[illegible]	[illegible]	[illegible]	[illegible]	[illegible]	[illegible]	[illegible]	[illegible]	[illegible]	[illegible]	[illegible]	[illegible]	[illegible]	[illegible]	[illegible]	[illegible]	[illegible]	[illegible]	[illegible]	[illegible]	[illegible]	[illegible]	[illegible]	[illegible]	[illegible]	[illegible]	[illegible]	[illegible]	[illegible]	[illegible]	[illegible]	[illegible]	[illegible]	[illegible]
BIHD1OS	1036 (+), 1284 (-), 331 (-), 445 (+),	-	[illegible]	[illegible]	[illegible]	[illegible]	[illegible]	[illegible]	[illegible]	[illegible]	[illegible]	[illegible]	[illegible]	[illegible]	[illegible]	[illegible]	[illegible]	[illegible]	[illegible]	[illegible]	[illegible]	[illegible]	[illegible]	[illegible]	[illegible]	[illegible]	[illegible]	[illegible]	[illegible]	[illegible]	[illegible]	[illegible]	[illegible]	[illegible]	[illegible]	[illegible]	[illegible]	[illegible]	[illegible]	[illegible]	[illegible]	[illegible]	[illegible]	[illegible]	[illegible]	[illegible]	[illegible]	[illegible]	[illegible]	[illegible]	[illegible]
BOXCPSAS1	-	-	[illegible]	[illegible]	[illegible]	[illegible]	[illegible]	[illegible]	[illegible]	[illegible]	[illegible]	[illegible]	[illegible]	[illegible]	[illegible]	[illegible]	[illegible]	[illegible]	[illegible]	[illegible]	[illegible]	[illegible]	[illegible]	[illegible]	[illegible]	[illegible]	[illegible]	[illegible]	[illegible]	[illegible]	[illegible]	[illegible]	[illegible]	[illegible]	[illegible]	[illegible]	[illegible]	[illegible]	[illegible]	[illegible]	[illegible]	[illegible]	[illegible]	[illegible]	[illegible]	[illegible]	[illegible]	[illegible]	[illegible]	[illegible]	[illegible]
BOXIINTPATPB	1456 (-),	975 (-),	[illegible]	[illegible]	[illegible]	[illegible]	[illegible]	[illegible]	[illegible]	[illegible]	[illegible]	[illegible]	[illegible]	[illegible]	[illegible]	[illegible]	[illegible]	[illegible]	[illegible]	[illegible]	[illegible]	[illegible]	[illegible]	[illegible]	[illegible]	[illegible]	[illegible]	[illegible]	[illegible]	[illegible]	[illegible]	[illegible]	[illegible]	[illegible]	[illegible]	[illegible]	[illegible]	[illegible]	[illegible]	[illegible]	[illegible]	[illegible]	[illegible]	[illegible]	[illegible]	[illegible]	[illegible]	[illegible]	[illegible]	[illegible]	[illegible]
BOXIIPCCHS	-	[illegible]	[illegible]	[illegible]	[illegible]	[illegible]	[illegible]	[illegible]	[illegible]	[illegible]	[illegible]	[illegible]	[illegible]	[illegible]	[illegible]	[illegible]	[illegible]	[illegible]	[illegible]	[illegible]	[illegible]	[illegible]	[illegible]	[illegible]	[illegible]	[illegible]	[illegible]	[illegible]	[illegible]	[illegible]	[illegible]	[illegible]	[illegible]	[illegible]	[illegible]	[illegible]	[illegible]	[illegible]	[illegible]	[illegible]	[illegible]	[illegible]	[illegible]	[illegible]	[illegible]	[illegible]	[illegible]	[illegible]	[illegible]	[illegible]	[illegible]
BOXLCOREDCPAL	-	1457 (-), 327 (+),	[illegible]	[illegible]	[illegible]	[illegible]	[illegible]	[illegible]	[illegible]	[illegible]	[illegible]	[illegible]	[illegible]	[illegible]	[illegible]	[illegible]	[illegible]	[illegible]	[illegible]	[illegible]	[illegible]	[illegible]	[illegible]	[illegible]	[illegible]	[illegible]	[illegible]	[illegible]	[illegible]	[illegible]	[illegible]	[illegible]	[illegible]	[illegible]	[illegible]	[illegible]	[illegible]	[illegible]	[illegible]	[illegible]	[illegible]	[illegible]	[illegible]	[illegible]	[illegible]	[illegible]	[illegible]	[illegible]	[illegible]	[illegible]	[illegible]
BP5OSWX	-	-	[illegible]	[illegible]	[illegible]	[illegible]	[illegible]	[illegible]	[illegible]	[illegible]	[illegible]	[illegible]	[illegible]	[illegible]	[illegible]	[illegible]	[illegible]	[illegible]	[illegible]	[illegible]	[illegible]	[illegible]	[illegible]	[illegible]	[illegible]	[illegible]	[illegible]	[illegible]	[illegible]	[illegible]	[illegible]	[illegible]	[illegible]	[illegible]	[illegible]	[illegible]	[illegible]	[illegible]	[illegible]	[illegible]	[illegible]	[illegible]	[illegible]	[illegible]	[illegible]	[illegible]	[illegible]	[illegible]	[illegible]	[illegible]	[illegible]
BS1EGCCR	1426 (-),	1439 (+),	[illegible]	[illegible]	[illegible]	[illegible]	[illegible]	[illegible]	[illegible]	[illegible]	[illegible]	[illegible]	[illegible]	[illegible]	[illegible]	[illegible]	[illegible]	[illegible]	[illegible]	[illegible]	[illegible]	[illegible]	[illegible]	[illegible]	[illegible]	[illegible]	[illegible]	[illegible]	[illegible]	[illegible]	[illegible]	[illegible]	[illegible]	[illegible]	[illegible]	[illegible]	[illegible]	[illegible]	[illegible]	[illegible]	[illegible]	[illegible]	[illegible]	[illegible]	[illegible]	[illegible]	[illegible]	[illegible]	[illegible]	[illegible]	[illegible]
CAATBOX1	1057 (-), 179 (-), 329 (-), 474 (+), 510 (-), 981 (-),	1335 (-), 1454 (-), 321 (-), 632 (+), 72 (+),	[illegible]	[illegible]	[illegible]	[illegible]	[illegible]	[illegible]	[illegible]	[illegible]	[illegible]	[illegible]	[illegible]	[illegible]	[illegible]	[illegible]	[illegible]	[illegible]	[illegible]	[illegible]	[illegible]	[illegible]	[illegible]	[illegible]	[illegible]	[illegible]	[illegible]	[illegible]	[illegible]	[illegible]	[illegible]	[illegible]	[illegible]	[illegible]	[illegible]	[illegible]	[illegible]	[illegible]	[illegible]	[illegible]	[illegible]	[illegible]	[illegible]	[illegible]	[illegible]	[illegible]	[illegible]	[illegible]	[illegible]	[illegible]	[illegible]
CACGCAATGMGH3	-	-	[illegible]	[illegible]	[illegible]	[illegible]	[illegible]	[illegible]	[illegible]	[illegible]	[illegible]	[illegible]	[illegible]	[illegible]	[illegible]	[illegible]	[illegible]	[illegible]	[illegible]	[illegible]	[illegible]	[illegible]	[illegible]	[illegible]	[illegible]	[illegible]	[illegible]	[illegible]	[illegible]	[illegible]	[illegible]	[illegible]	[illegible]	[illegible]	[illegible]	[illegible]	[illegible]	[illegible]	[illegible]	[illegible]	[illegible]	[illegible]	[illegible]	[illegible]	[illegible]	[illegible]	[illegible]	[illegible]	[illegible]	[illegible]	[illegible]
CACGTGMOTIF	-	130 (+), 130 (-),	[illegible]	[illegible]	[illegible]	[illegible]	[illegible]	[illegible]	[illegible]	[illegible]	[illegible]	[illegible]	[illegible]	[illegible]	[illegible]	[illegible]	[illegible]	[illegible]	[illegible]	[illegible]	[illegible]	[illegible]	[illegible]	[illegible]	[illegible]	[illegible]	[illegible]	[illegible]	[illegible]	[illegible]	[illegible]	[illegible]	[illegible]	[illegible]	[illegible]	[illegible]	[illegible]	[illegible]	[illegible]	[illegible]	[illegible]	[illegible]	[illegible]	[illegible]	[illegible]	[illegible]	[illegible]	[illegible]	[illegible]	[illegible]	[illegible]
CACTFTPPCA1	1073 (+), 1079 (-), 1111 (-), 1253 (+), 1260 (+), 1422 (+), 418 (+), 527 (-), 529 (+), 534 (+), 541 (-), 628 (-),	105 (-), 344 (+), 365 (-), 459 (+), 693 (-), 732 (-),	[illegible]	[illegible]	[illegible]	[illegible]	[illegible]	[illegible]	[illegible]	[illegible]	[illegible]	[illegible]	[illegible]	[illegible]	[illegible]	[illegible]	[illegible]	[illegible]	[illegible]	[illegible]	[illegible]	[illegible]	[illegible]	[illegible]	[illegible]	[illegible]	[illegible]	[illegible]	[illegible]	[illegible]	[illegible]	[illegible]	[illegible]	[illegible]	[illegible]	[illegible]	[illegible]	[illegible]	[illegible]	[illegible]	[illegible]	[illegible]	[illegible]	[illegible]	[illegible]	[illegible]	[illegible]	[illegible]	[illegible]	[illegible]	[illegible]
CANBNNAPA	-	-	[illegible]	[illegible]	[illegible]	[illegible]	[illegible]	[illegible]	[illegible]	[illegible]	[illegible]	[illegible]	[illegible]	[illegible]	[illegible]	[illegible]	[illegible]	[illegible]	[illegible]	[illegible]	[illegible]	[illegible]	[illegible]	[illegible]	[illegible]	[illegible]	[illegible]	[illegible]	[illegible]	[illegible]	[illegible]	[illegible]	[illegible]	[illegible]	[illegible]	[illegible]	[illegible]	[illegible]	[illegible]	[illegible]	[illegible]	[illegible]	[illegible]	[illegible]	[illegible]	[illegible]	[illegible]	[illegible]	[illegible]	[illegible]	[illegible]
CAREOSREP1	-	138 (-),	[illegible]	[illegible]	[illegible]	[illegible]	[illegible]	[illegible]	[illegible]	[illegible]	[illegible]	[illegible]	[illegible]	[illegible]	[illegible]	[illegible]	[illegible]	[illegible]	[illegible]	[illegible]	[illegible]	[illegible]	[illegible]	[illegible]	[illegible]	[illegible]	[illegible]	[illegible]	[illegible]	[illegible]	[illegible]	[illegible]	[illegible]	[illegible]	[illegible]	[illegible]	[illegible]	[illegible]	[illegible]	[illegible]	[illegible]	[illegible]	[illegible]	[illegible]	[illegible]	[illegible]	[illegible]	[illegible]	[illegible]	[illegible]	[illegible]
CARGCW8GAT	-	-	[illegible]	[illegible]	[illegible]	[illegible]	[illegible]	[illegible]	[illegible]	[illegible]	[illegible]	[illegible]	[illegible]	[illegible]	[illegible]	[illegible]	[illegible]	[illegible]	[illegible]	[illegible]	[illegible]	[illegible]	[illegible]	[illegible]	[illegible]	[illegible]	[illegible]	[illegible]	[illegible]	[illegible]	[illegible]	[illegible]	[illegible]	[illegible]	[illegible]	[illegible]	[illegible]	[illegible]	[illegible]	[illegible]	[illegible]	[illegible]	[illegible]	[illegible]	[illegible]	[illegible]	[illegible]	[illegible]	[illegible]	[illegible]	[illegible]
CARGNCAT	-	-	[illegible]	[illegible]	[illegible]	[illegible]	[illegible]	[illegible]	[illegible]	[illegible]	[illegible]	[illegible]	[illegible]	[illegible]	[illegible]	[illegible]	[illegible]	[illegible]	[illegible]	[illegible]	[illegible]	[illegible]	[illegible]	[illegible]	[illegible]	[illegible]	[illegible]	[illegible]	[illegible]	[illegible]	[illegible]	[illegible]	[illegible]	[illegible]	[illegible]	[illegible]	[illegible]	[illegible]	[illegible]	[illegible]	[illegible]	[illegible]	[illegible]	[illegible]	[illegible]	[illegible]	[illegible]	[illegible]	[illegible]	[illegible]	[illegible]
CATATGGMSAUR	1045 (+), 1045 (-), 617 (+), 617 (-)	-	[illegible]	[illegible]	[illegible]	[illegible]	[illegible]	[illegible]	[illegible]	[illegible]	[illegible]	[illegible]	[illegible]	[illegible]	[illegible]	[illegible]	[illegible]	[illegible]	[illegible]	[illegible]	[illegible]	[illegible]	[illegible]	[illegible]	[illegible]	[illegible]	[illegible]	[illegible]	[illegible]	[illegible]	[illegible]	[illegible]	[illegible]	[illegible]	[illegible]	[illegible]	[illegible]	[illegible]	[illegible]	[illegible]	[illegible]	[illegible]	[illegible]	[illegible]	[illegible]	[illegible]	[illegible]	[illegible]	[illegible]	[illegible]	[illegible]
CBFHV	1040 (+), 249 (+), 249 (-), 830 (-),	1132 (+), 1162 (+),	[illegible]	[illegible]	[illegible]	[illegible]	[illegible]	[illegible]	[illegible]	[illegible]	[illegible]	[illegible]	[illegible]	[illegible]	[illegible]	[illegible]	[illegible]	[illegible]	[illegible]	[illegible]	[illegible]	[illegible]	[illegible]	[illegible]	[illegible]	[illegible]	[illegible]	[illegible]	[illegible]	[illegible]	[illegible]	[illegible]	[illegible]	[illegible]	[illegible]	[illegible]	[illegible]	[illegible]	[illegible]	[illegible]	[illegible]	[illegible]	[illegible]	[illegible]	[illegible]	[illegible]	[illegible]	[illegible]	[illegible]	[illegible]	[illegible]
CCA1ATLHCB1	[illegible]	-	[illegible]	[illegible]	[illegible]	[illegible]	[illegible]	[illegible]	[illegible]	[illegible]	[illegible]	[illegible]	[illegible]	[illegible]	[illegible]	[illegible]	[illegible]	[illegible]	[illegible]	[illegible]	[illegible]	[illegible]	[illegible]	[illegible]	[illegible]	[illegible]	[illegible]	[illegible]	[illegible]	[illegible]	[illegible]	[illegible]	[illegible]	[illegible]	[illegible]	[illegible]	[illegible]	[illegible]	[illegible]	[illegible]	[illegible]	[illegible]	[illegible]	[illegible]	[illegible]	[illegible]	[illegible]	[illegible]	[illegible]	[illegible]	[illegible]
CCAATBOX1	179 (-), 510 (-),	1335 (-), 1454 (-), 631 (+), 71 (+),	[illegible]	[illegible]	[illegible]	[illegible]	[illegible]	[illegible]	[illegible]	[illegible]	[illegible]	[illegible]	[illegible]	[illegible]	[illegible]	[illegible]	[illegible]	[illegible]	[illegible]	[illegible]	[illegible]	[illegible]	[illegible]	[illegible]	[illegible]	[illegible]	[illegible]	[illegible]	[illegible]	[illegible]	[illegible]	[illegible]	[illegible]	[illegible]	[illegible]	[illegible]	[illegible]	[illegible]	[illegible]	[illegible]	[illegible]	[illegible]	[illegible]	[illegible]	[illegible]	[illegible]	[illegible]	[illegible]	[illegible]	[illegible]	[illegible]
CDA1ATCAB2	-	-	[illegible]	[illegible]	[illegible]	[illegible]	[illegible]	[illegible]	[illegible]	[illegible]	[illegible]	[illegible]	[illegible]	[illegible]	[illegible]	[illegible]	[illegible]	[illegible]	[illegible]	[illegible]	[illegible]	[illegible]	[illegible]	[illegible]	[illegible]	[illegible]	[illegible]	[illegible]	[illegible]	[illegible]	[illegible]	[illegible]	[illegible]	[illegible]	[illegible]	[illegible]	[illegible]	[illegible]	[illegible]	[illegible]	[illegible]	[illegible]	[illegible]	[illegible]	[illegible]	[illegible]	[illegible]	[illegible]	[illegible]	[illegible]	[illegible]
CELLCYCLESC	-	-	[illegible]	[illegible]	[illegible]	[illegible]	[illegible]	[illegible]	[illegible]	[illegible]	[illegible]	[illegible]	[illegible]	[illegible]	[illegible]	[illegible]	[illegible]	[illegible]	[illegible]	[illegible]	[illegible]	[illegible]	[illegible]	[illegible]	[illegible]	[illegible]	[illegible]	[illegible]	[illegible]	[illegible]	[illegible]	[illegible]	[illegible]	[illegible]	[illegible]	[illegible]	[illegible]	[illegible]	[illegible]	[illegible]	[illegible]	[illegible]	[illegible]	[illegible]	[illegible]	[illegible]	[illegible]	[illegible]	[illegible]	[illegible]	[illegible]
CEREGLUBOX1PSLEGA	-	-	[illegible]	[illegible]	[illegible]	[illegible]	[illegible]	[illegible]	[illegible]	[illegible]	[illegible]	[illegible]	[illegible]	[illegible]	[illegible]	[illegible]	[illegible]	[illegible]	[illegible]	[illegible]	[illegible]	[illegible]	[illegible]	[illegible]	[illegible]	[illegible]	[illegible]	[illegible]	[illegible]	[illegible]	[illegible]	[illegible]	[illegible]	[illegible]	[illegible]	[illegible]	[illegible]	[illegible]	[illegible]	[illegible]	[illegible]	[illegible]	[illegible]	[illegible]	[illegible]	[illegible]	[illegible]	[illegible]	[illegible]	[illegible]	[illegible]
CEREGLUBOX2PSLEGA	-	-	[illegible]	[illegible]	[illegible]	[illegible]	[illegible]	[illegible]	[illegible]	[illegible]	[illegible]	[illegible]	[illegible]	[illegible]	[illegible]	[illegible]	[illegible]	[illegible]	[illegible]	[illegible]	[illegible]	[illegible]	[illegible]	[illegible]	[illegible]	[illegible]	[illegible]	[illegible]	[illegible]	[illegible]	[illegible]	[illegible]	[illegible]	[illegible]	[illegible]	[illegible]	[illegible]	[illegible]	[illegible]	[illegible]	[illegible]	[illegible]	[illegible]	[illegible]	[illegible]	[illegible]	[illegible]	[illegible]	[illegible]	[illegible]	[illegible]
CEREGLUBOX3PSLEGA	-	-	[illegible]	[illegible]	[illegible]	[illegible]	[illegible]	[illegible]	[illegible]	[illegible]	[illegible]	[illegible]	[illegible]	[illegible]	[illegible]	[illegible]	[illegible]	[illegible]	[illegible]	[illegible]	[illegible]	[illegible]	[illegible]	[illegible]	[illegible]	[illegible]	[illegible]	[illegible]	[illegible]	[illegible]	[illegible]	[illegible]	[illegible]	[illegible]	[illegible]	[illegible]	[illegible]	[illegible]	[illegible]	[illegible]	[illegible]	[illegible]	[illegible]	[illegible]	[illegible]	[illegible]	[illegible]	[illegible]	[illegible]	[illegible]	[illegible]
CGACGOSAMY3	1329 (+), 1375 (-), 236 (+), 239 (+), 242 (+), 245 (+), 248 (-), 251 (+), 820 (+), 841 (-),	1124 (+), 220 (+), 277 (+),	[illegible]	[illegible]	[illegible]	[illegible]	[illegible]	[illegible]	[illegible]	[illegible]	[illegible]	[illegible]	[illegible]	[illegible]	[illegible]	[illegible]	[illegible]	[illegible]	[illegible]	[illegible]	[illegible]	[illegible]	[illegible]	[illegible]	[illegible]	[illegible]	[illegible]	[illegible]	[illegible]	[illegible]	[illegible]	[illegible]	[illegible]	[illegible]	[illegible]	[illegible]	[illegible]	[illegible]	[illegible]	[illegible]	[illegible]	[illegible]	[illegible]	[illegible]	[illegible]	[illegible]	[illegible]	[illegible]	[illegible]	[illegible]	[illegible]
CGCGBOXAT	12 (+), 12 (-), 136 (+), 136 (-), 1360 (+), 1360 (-), 1362 (+), 1362 (-), 1381 (+), 1381 (-), 1410 (+), 1410 (-), 1441 (+), 1441 (-), 189 (+), 189 (-), 637 (+), 637 (-), 68 (+), 68 (-), 836 (+), 836 (-), 838 (+), 838 (-),	1118 (+), 1118 (-), 1137 (+), 1137 (-), 1174 (+), 1174 (-), 1247 (+), 1247 (-), 1414 (+), 1414 (-), 1446 (+), 1446 (-), 565 (+), 565 (-), 775 (+), 775 (-),	[illegible]	[illegible]	[illegible]	[illegible]	[illegible]	[illegible]	[illegible]	[illegible]	[illegible]	[illegible]	[illegible]	[illegible]	[illegible]	[illegible]	[illegible]	[illegible]	[illegible]	[illegible]	[illegible]	[illegible]	[illegible]	[illegible]	[illegible]	[illegible]	[illegible]	[illegible]	[illegible]	[illegible]	[illegible]	[illegible]	[illegible]	[illegible]	[illegible]	[illegible]	[illegible]	[illegible]	[illegible]	[illegible]	[illegible]	[illegible]	[illegible]	[illegible]	[illegible]	[illegible]	[illegible]	[illegible]	[illegible]	[illegible]	[illegible]
CIACADIANLELHC	1028 (-),	-	[illegible]	[illegible]	[illegible]	[illegible]	[illegible]	[illegible]	[illegible]	[illegible]	[illegible]	[illegible]	[illegible]	[illegible]	[illegible]	[illegible]	[illegible]	[illegible]	[illegible]	[illegible]	[illegible]	[illegible]	[illegible]	[illegible]	[illegible]	[illegible]	[illegible]	[illegible]	[illegible]	[illegible]	[illegible]	[illegible]	[illegible]	[illegible]	[illegible]	[illegible]	[illegible]	[illegible]	[illegible]	[illegible]	[illegible]	[illegible]	[illegible]	[illegible]	[illegible]	[illegible]	[illegible]	[illegible]	[illegible]	[illegible]	[illegible]
CMSRE1IBSPOA	364 (+),	-	[illegible]	[illegible]	[illegible]	[illegible]	[illegible]	[illegible]	[illegible]	[illegible]	[illegible]	[illegible]	[illegible]	[illegible]	[illegible]	[illegible]	[illegible]	[illegible]	[illegible]	[illegible]	[illegible]	[illegible]	[illegible]	[illegible]	[illegible]	[illegible]	[illegible]	[illegible]	[illegible]	[illegible]	[illegible]	[illegible]	[illegible]	[illegible]	[illegible]	[illegible]	[illegible]	[illegible]	[illegible]	[illegible]	[illegible]	[illegible]	[illegible]	[illegible]	[illegible]	[illegible]	[illegible]	[illegible]	[illegible]	[illegible]	[illegible]
CPBCSPOR	536 (-),	-	[illegible]	[illegible]	[illegible]	[illegible]	[illegible]	[illegible]	[illegible]	[illegible]	[illegible]	[illegible]	[illegible]	[illegible]	[illegible]	[illegible]	[illegible]	[illegible]	[illegible]	[illegible]	[illegible]	[illegible]	[illegible]	[illegible]	[illegible]	[illegible]	[illegible]	[illegible]	[illegible]	[illegible]	[illegible]	[illegible]	[illegible]	[illegible]	[illegible]	[illegible]	[illegible]	[illegible]	[illegible]	[illegible]	[illegible]	[illegible]	[illegible]	[illegible]	[illegible]	[illegible]	[illegible]	[illegible]	[illegible]	[illegible]	[illegible]
CPRFPCCHS	-	-	[illegible]	[illegible]	[illegible]	[illegible]	[illegible]	[illegible]	[illegible]	[illegible]	[illegible]	[illegible]	[illegible]	[illegible]	[illegible]	[illegible]	[illegible]	[illegible]	[illegible]	[illegible]	[illegible]	[illegible]	[illegible]	[illegible]	[illegible]	[illegible]	[illegible]	[illegible]	[illegible]	[illegible]	[illegible]	[illegible]	[illegible]	[illegible]	[illegible]	[illegible]	[illegible]	[illegible]	[illegible]	[illegible]	[illegible]	[illegible]	[illegible]	[illegible]	[illegible]	[illegible]	[illegible]	[illegible]	[illegible]	[illegible]	[illegible]
CRTDREHVCBF2	249 (+), [illegible]	-	[illegible]	[illegible]	[illegible]	[illegible]	[illegible]	[illegible]	[illegible]	[illegible]	[illegible]	[illegible]	[illegible]	[illegible]	[illegible]	[illegible]	[illegible]	[illegible]	[illegible]	[illegible]	[illegible]	[illegible]	[illegible]	[illegible]	[illegible]	[illegible]	[illegible]	[illegible]	[illegible]	[illegible]	[illegible]	[illegible]	[illegible]	[illegible]	[illegible]	[illegible]	[illegible]	[illegible]	[illegible]	[illegible]	[illegible]	[illegible]	[illegible]	[illegible]	[illegible]	[illegible]	[illegible]	[illegible]	[illegible]	[illegible]	[illegible]
CTRMCAMV35S	-	-	[illegible]	[illegible]	[illegible]	[illegible]	[illegible]	[illegible]	[illegible]	[illegible]	[illegible]	[illegible]	[illegible]	[illegible]	[illegible]	[illegible]	[illegible]	[illegible]	[illegible]	[illegible]	[illegible]	[illegible]	[illegible]	[illegible]	[illegible]	[illegible]	[illegible]	[illegible]	[illegible]	[illegible]	[illegible]	[illegible]	[illegible]	[illegible]	[illegible]	[illegible]	[illegible]	[illegible]	[illegible]	[illegible]	[illegible]	[illegible]	[illegible]	[illegible]	[illegible]	[illegible]	[illegible]	[illegible]	[illegible]	[illegible]	[illegible]
CURECORECR	108 (+), 108 (-), 1490 (+), 1490 (-), 528 (+), 528 (-), 542 (+), 542 (-), 745 (+), 745 (-),	1472 (+), 1472 (-),	[illegible]	[illegible]	[illegible]	[illegible]	[illegible]	[illegible]	[illegible]	[illegible]	[illegible]	[illegible]	[illegible]	[illegible]	[illegible]	[illegible]	[illegible]	[illegible]	[illegible]	[illegible]	[illegible]	[illegible]	[illegible]	[illegible]	[illegible]	[illegible]	[illegible]	[illegible]	[illegible]	[illegible]	[illegible]	[illegible]	[illegible]	[illegible]	[illegible]	[illegible]	[illegible]	[illegible]	[illegible]	[illegible]	[illegible]	[illegible]	[illegible]	[illegible]	[illegible]	[illegible]	[illegible]	[illegible]	[illegible]	[illegible]	[illegible]
DOFCOREZM	1033 (-), 1142 (-), 1147 (-), 1214 (+), 1269 (+), 285 (+), 296 (-), 420 (-), 489 (-), 493 (-), 552 (+), 737 (-), 958 (+), 963 (+),	1026 (-), 1034 (-), 169 (-), 210 (+), 417 (-), 470 (+), 480 (+), 513 (+), 517 (+), 604 (-), 691 (+), 904 (-), 972 (-),	[illegible]	[illegible]	[illegible]	[illegible]	[illegible]	[illegible]	[illegible]	[illegible]	[illegible]	[illegible]	[illegible]	[illegible]	[illegible]	[illegible]	[illegible]	[illegible]	[illegible]	[illegible]	[illegible]	[illegible]	[illegible]	[illegible]	[illegible]	[illegible]	[illegible]	[illegible]	[illegible]	[illegible]	[illegible]	[illegible]	[illegible]	[illegible]	[illegible]	[illegible]	[illegible]	[illegible]	[illegible]	[illegible]	[illegible]	[illegible]	[illegible]	[illegible]	[illegible]	[illegible]	[illegible]	[illegible]	[illegible]
DPBFCOREDCDC3	1020 (+), 1274 (-), 201 (-), 949 (-),	11 (-), 181 (-), 463 (+),	[illegible]	[illegible]	[illegible]	[illegible]	[illegible]	[illegible]	[illegible]	[illegible]	[illegible]	[illegible]	[illegible]	[illegible]	[illegible]	[illegible]	[illegible]	[illegible]	[illegible]	[illegible]	[illegible]	[illegible]	[illegible]	[illegible]	[illegible]	[illegible]	[illegible]	[illegible]	[illegible]	[illegible]	[illegible]	[illegible]	[illegible]	[illegible]	[illegible]	[illegible]	[illegible]	[illegible]	[illegible]	[illegible]	[illegible]	[illegible]	[illegible]	[illegible]	[illegible]	[illegible]	[illegible]	[illegible]	[illegible]	[illegible]	[illegible]
DRE1COREZMRAB17	[illegible]	-	[illegible]	[illegible]	[illegible]	[illegible]	[illegible]	[illegible]	[illegible]	[illegible]	[illegible]	[illegible]	[illegible]	[illegible]	[illegible]	[illegible]	[illegible]	[illegible]	[illegible]	[illegible]	[illegible]	[illegible]	[illegible]	[illegible]	[illegible]	[illegible]	[illegible]	[illegible]	[illegible]	[illegible]	[illegible]	[illegible]	[illegible]	[illegible]	[illegible]	[illegible]	[illegible]	[illegible]	[illegible]	[illegible]	[illegible]	[illegible]	[illegible]	[illegible]	[illegible]	[illegible]	[illegible]	[illegible]	[illegible]	[illegible]	[illegible]
DRE2COREZMRAB17	1040 (+),	-	[illegible]	[illegible]	[illegible]	[illegible]	[illegible]	[illegible]	[illegible]	[illegible]	[illegible]	[illegible]	[illegible]	[illegible]	[illegible]	[illegible]	[illegible]	[illegible]	[illegible]	[illegible]	[illegible]	[illegible]	[illegible]	[illegible]	[illegible]	[illegible]	[illegible]	[illegible]	[illegible]	[illegible]	[illegible]	[illegible]	[illegible]	[illegible]	[illegible]	[illegible]	[illegible]	[illegible]	[illegible]	[illegible]	[illegible]	[illegible]	[illegible]	[illegible]	[illegible]	[illegible]	[illegible]	[illegible]	[illegible]	[illegible]	[illegible]
DRECRTCOREAT	1040 (+),	1132 (+), 1162 (+),	[illegible]	[illegible]	[illegible]	[illegible]	[illegible]	[illegible]	[illegible]	[illegible]	[illegible]	[illegible]	[illegible]	[illegible]	[illegible]	[illegible]	[illegible]	[illegible]	[illegible]	[illegible]	[illegible]	[illegible]	[illegible]	[illegible]	[illegible]	[illegible]	[illegible]	[illegible]	[illegible]	[illegible]	[illegible]	[illegible]	[illegible]	[illegible]	[illegible]	[illegible]	[illegible]	[illegible]	[illegible]	[illegible]	[illegible]	[illegible]	[illegible]	[illegible]	[illegible]	[illegible]	[illegible]	[illegible]	[illegible]	[illegible]	[illegible]
E2F1OSPCNA	-	-	[illegible]	[illegible]	[illegible]	[illegible]	[illegible]	[illegible]	[illegible]	[illegible]	[illegible]	[illegible]	[illegible]	[illegible]	[illegible]	[illegible]	[illegible]	[illegible]	[illegible]	[illegible]	[illegible]	[illegible]	[illegible]	[illegible]	[illegible]	[illegible]	[illegible]	[illegible]	[illegible]	[illegible]	[illegible]	[illegible]	[illegible]	[illegible]	[illegible]	[illegible]	[illegible]	[illegible]	[illegible]	[illegible]	[illegible]	[illegible]	[illegible]	[illegible]	[illegible]	[illegible]	[illegible]	[illegible]	[illegible]	[illegible]	[illegible]
E2FANTRNR	-	-	[illegible]	[illegible]	[illegible]	[illegible]	[illegible]	[illegible]	[illegible]	[illegible]	[illegible]	[illegible]	[illegible]	[illegible]	[illegible]	[illegible]	[illegible]	[illegible]	[illegible]	[illegible]	[illegible]	[illegible]	[illegible]	[illegible]	[illegible]	[illegible]	[illegible]	[illegible]	[illegible]	[illegible]	[illegible]	[illegible]	[illegible]	[illegible]	[illegible]	[illegible]	[illegible]	[illegible]	[illegible]	[illegible]	[illegible]	[illegible]	[illegible]	[illegible]	[illegible]	[illegible]	[illegible]	[illegible]	[illegible]	[illegible]	[illegible]
E2FAT	1431 (+)	-	[illegible]	[illegible]	[illegible]	[illegible]	[illegible]	[illegible]	[illegible]	[illegible]	[illegible]	[illegible]	[illegible]	[illegible]	[illegible]	[illegible]	[illegible]	[illegible]	[illegible]	[illegible]	[illegible]	[illegible]	[illegible]	[illegible]	[illegible]	[illegible]	[illegible]	[illegible]	[illegible]	[illegible]	[illegible]	[illegible]	[illegible]	[illegible]	[illegible]	[illegible]	[illegible]	[illegible]	[illegible]	[illegible]	[illegible]	[illegible]	[illegible]	[illegible]	[illegible]	[illegible]	[illegible]	[illegible]	[illegible]	[illegible]	[illegible]
E2FBNTRNR	-	-	[illegible]	[illegible]	[illegible]	[illegible]	[illegible]	[illegible]	[illegible]	[illegible]	[illegible]	[illegible]	[illegible]	[illegible]	[illegible]	[illegible]	[illegible]	[illegible]	[illegible]	[illegible]	[illegible]	[illegible]	[illegible]	[illegible]	[illegible]	[illegible]	[illegible]	[illegible]	[illegible]	[illegible]	[illegible]	[illegible]	[illegible]	[illegible]	[illegible]	[illegible]	[illegible]	[illegible]	[illegible]	[illegible]	[illegible]	[illegible]	[illegible]	[illegible]	[illegible]	[illegible]	[illegible]	[illegible]	[illegible]	[illegible]	[illegible]
E2FCONSENSUS	1390 (-), 1412 (-), 423 (+)	1237 (+), 492 (+),	[illegible]	[illegible]	[illegible]	[illegible]	[illegible]	[illegible]	[illegible]	[illegible]	[illegible]	[illegible]	[illegible]	[illegible]	[illegible]	[illegible]	[illegible]	[illegible]	[illegible]	[illegible]	[illegible]	[illegible]	[illegible]	[illegible]	[illegible]	[illegible]	[illegible]	[illegible]	[illegible]	[illegible]	[illegible]	[illegible]	[illegible]	[illegible]	[illegible]	[illegible]	[illegible]	[illegible]	[illegible]	[illegible]	[illegible]	[illegible]	[illegible]	[illegible]	[illegible]	[illegible]	[illegible]	[illegible]	[illegible]	[illegible]	[illegible]

EBOXBNNAPA	1045 (+), 1045 (-), 1102 (+), 1102 (-), 1208 (+), 1208 (-), 1470 (+), 1470 (-), 438 (+), 438 (-), 617 (+), 617 (-), 946 (+), 946 (-),	130 (+), 130 (-), 41 (+), 41 (-), 464 (+), 464 (-), 549 (+), 549 (-),	[illegible]
EECCRCAH1	991 (+),	1326 (-), 1373 (-), 369 (+), 516 (-), 87 [illegible]	[illegible]
ELRECOREPCRP1	1126 (+),	1196 (-),	[illegible]
EMBP1TAEM	-	-	[illegible]
EMHVCHORD	-	-	[illegible]
ERELEE4	-	-	[illegible]
EVENINGAT	-	-	[illegible]
GADOWNAT	-	-	[illegible]
GAGA8HVBKN3	-	-	[illegible]
GAGAGMGSA1	-	-	[illegible]
GARE1OSREP1	561 (+)	-	[illegible]
GARE2OSREP1	543 (-)	-	[illegible]
GAREAT	-	-	[illegible]
GATABOX	466 (+), 928 (+),	1088 (-), 206 (+), 248 (-), 317 (+), 455 (+), 752 (+), 792 (+),	[illegible]
GBOX10NT	-	-	[illegible]
GBOXLERBCS	-	-	[illegible]
GCBP2ZMGAPC4	-	-	[illegible]
GCCCORE	129 (+), 1437 (+), 1445 (+), 154 (+),	1159 (+), 1187 (+), 1220 (+), 1241 (+), 1418 (-), 1421 (-), 1424 (-),	[illegible]
GCN4OSGLUB1	-	-	[illegible]
GLMHVCHORD	-	-	[illegible]
GRAZMRAB28	-	-	[illegible]
GT1CONSENSUS	1082 (+), 1136 (-), 1137 (-), 1151 (-), 1152 (-), 1350 (+), 1461 (-),	1020 (-), 1021 (-), 1028 (-), 1029 (-), 206 (+), 317 (+), 433 (-), 582 (+), 65 (-), 66 (-), 673 (+), 735 (+), 767 (-), 813 (-), 814 (-),	[illegible]
GT1CORE	-	532 (+),	[illegible]
GT1GMSCAM4	1136 (-), 1151 (-), 1350 (+),	1020 (-), 1028 (-), 65 (-), 673 (+),	[illegible]
GT1MOTIFPSRBCS	-	731 (-)	[illegible]
GTGANTG10	1 (+), 1038 (-), 411 (+), 758 (-), 813 (+), 82 (-), 89 (-), 943 (-),	133 (+), 1406 (+), 224 (+), 420 (-), 528 (-), 607 (-), 635 (-), 707 (-),	[illegible]
HBOXCONSENSUSPVCHS	-	-	[illegible]
HDZIP2ATATHB2	-	-	[illegible]
HEXAMERATH4	1329 (-), 1374 (+), 251 (-), 820 [illegible]	-	[illegible]
HEXAT	748 (-)	-	[illegible]
HEXMOTIFTAH3H4	1368 (-), 750 (+),	225 (-),	[illegible]
IBOX	-	-	[illegible]
IBOXCORE	-	206 (+), 317 (+),	[illegible]
IBOXCORENT	-	-	[illegible]
INRNTPSADB	1216 (-), 765 (+),	-	[illegible]
INTRONLOWER	227 (+), 503 (+), 786 [illegible]	35 (+), 695 (+),	[illegible]
INTRONUPPER	-	-	[illegible]
IRO2OS	-	-	[illegible]
L1BOXATPDF1	-	-	[illegible]
LEAFYATAG	-	-	[illegible]
LECPLEACS2	-	-	[illegible]
LREBOXIIPCCHS1	-	-	[illegible]
LRENPCABE	-	279 (+)	[illegible]
LTRE1HVBLT49	-	153 (-), 388 (+),	[illegible]
LTREATLTI78	1040 (+)	-	[illegible]
LTRECOREATCOR15	1041 (+), 1308 (+), 53 (+),	1133 (+), 1163 (+), 276 (+),	[illegible]
MARABOX1	-	1008 (-), 1012 (-)	[illegible]
MARARS	-	-	[illegible]
MARTBOX	-	170 (+), 674 (-),	[illegible]
MNF1ZMPPC1	-	-	[illegible]
MSACRCYM	-	-	[illegible]
MYB1AT	1418 (+),	628 (+),	[illegible]
MYB1LEPR	-	-	[illegible]
MYB26PS	-	-	[illegible]
MYB2AT	-	450 (+),	[illegible]
MYB2CONSENSUSAT	1102 (+),	1 (+), 1199 (+), 1215 (+), 41 (-), 450 (+), 543 (-), 549 (-), 578 (+), 621 (-), 8 (+),	[illegible]
MYBATRD22	-	-	[illegible]
MYBCORE	1102 (-), 217 (+), 561 (-), 608 (-),	1 (-), 1199 (-), 1215 (-), 156 (+), 196 (+), 329 (-), 41 (+), 450 (-), 531 (+), 543 (+), 549 (+), 578 (-), 621 (+), 648 (+), 8 (-), [illegible]	[illegible]
MYBCOREATCYCB1	-	1200 (+), 1216 (+), 1314 (-), 2 (+), 543 (-), 579 (+), 621 (-), 713 (-), 9 (+),	[illegible]
MYBGAHV	-	-	[illegible]
MYBPLANT	-	326 (+),	[illegible]
MYBPZM	166 (-), 905 (-),	1293 (-), 157 (-), 328 (+), 850 (+),	[illegible]
MYBST1	465 (+), 927 (+),	1088 (-), 248 (-), 316 (+),	[illegible]
MYCATERD1	-	464 (-),	[illegible]
MYCATRD22	-	464 (+),	[illegible]
MYCCONSENSUSAT	1045 (+), 1045 (-), 1102 (+), 1102 (-), 1208 (+), 1208 (-), 1470 (+), 1470 (-), 438 (+), 438 (-), 617 (+), 617 (-), 946 (+), 946 (-),	130 (+), 130 (-), 41 (+), 41 (-), 464 (+), 464 (-), 549 (+), 549 (-),	[illegible]
NAPINMOTIFBN	-	462 (+),	[illegible]
NODCON1GM	-	-	[illegible]
NODCON2GM	-	-	[illegible]
NONAMERATH4	-	-	[illegible]
NONAMERMOTIFTAH3H4	-	-	[illegible]
NRRBNEXTA	-	-	[illegible]
NTBBF1ARROLB	-	-	[illegible]
OCTAMERMOTIFTAH3H4	-	-	[illegible]
OCTAMOTIF2	-	-	[illegible]
OSE1ROOTNODULE	-	-	[illegible]
OSE2ROOTNODULE	-	-	[illegible]
P1BS	-	-	[illegible]
PALBOXAPC	365 (-),	-	[illegible]
PALBOXLPC	-	-	[illegible]
PALINDROMICCBOXGM	-	-	[illegible]
PIATGAPB	-	-	[illegible]
POLASIG1	297 (-),	1008 (-), 1012 (-), 1016 (-), 665 (-), 810 [illegible]	[illegible]
POLASIG2	-	507 (+),	[illegible]
POLASIG3	176 (-),	1048 (+), 73 (+), 740 (+), 743 (+), 746 [illegible]	[illegible]
POLLEN1LELAT52	1162 (+), 1349 (+), 1455 (-), 490 (-), 778 (-), 956 (+),	1035 (-), 414 (-), 515 (+), 769 (-), 974 (-),	[illegible]
PREATPRODH	-	136 (-),	[illegible]
PRECONSCRHSP70A	1026 (+), 1357 (-), 53 (+),	1133 (+), 1390 (+), 201 (+), 634 (-),	[illegible]
PROLAMINBOXOSGLUB1	-	904 (-),	[illegible]
PROXBBNNAPA	-	-	[illegible]
PYRIMIDINEBOXHVEPB1	1150 (+),	1027 (+), 64 (+)	[illegible]

PYRIMIDINEBOXOSRAMY	1146 (+),	1025 (+), 168 (+), 209 (-), 971 (+)	951 (+), 994 (+),	745 (-),	1059 (-), 350 (-),	891 (+),	321 (-), 639 (-),	492 (+), 648 (+),	1389 (-),	207 (+),	435 (+), 995 (+),	1046 (+),	435 (+),	-	-	-	332 (-),	-	289 (+), 455 (+), 69 (+), 714 (+),	-	1277 (-), 129 (+), 533 (-),	127 (+),	1335 (+),	-	206 (+), 275 (-),
QARBNEXTA	-	422 (-),	-	-	-	-	-	-	-	-	-	-	-	-	-	-	-	-	-	-	-	1328 (+), 991 (+)	-	-	-
QELEMENTZMZM13	-	1486 (-),	-	-	302 (-),	647 (-),	-	-	-	247 (-),	480 (-),	-	181 (-), 749 (+)	-	812 (+),	-	-	-	66 (-),	-	-	-	-	-	-
RAV1AAT	205 (-), 218 (-), 321 (-),	185 (-), 197 (-), 572 (+),	632 (+), 730 (+), 733 (+),	1019 (+), 334 (-), 540 (-), 621 (-), 641 (-), 689 (-), 712 (+), 765 (-),	238 (+), 28 (+), 335 (-), 988 (+),	1193 (+), 281 (-), 383 (-), 925 (-),	488 (+), 5 (+), 618 (-),	1186 (+), 1224 (+), 265 (-), 341 (-), 352 (-), 359 (-), 540 (-),	380 (-), 387 (+), 673 (+),	244 (-), 349 (-),	1053 (+), 198 (+), 265 (+), 349 (+),	1074 (+), 1152 (-), 1361 (-), 1398 (-), 526 (+),	115 (-), 1393 (-), 144 (-), 201 (-), 606 (-),	1385 (-), 1411 (+), 407 (-),	295 (-), 322 (-),	11 (-), 1155 (-), 1232 (+), 700 (-),	715 (-), 943 (-),	1026 (-), 190 (-), 364 (-), 530 (-), 604 (+), 756 (-), 818 (+), 967 (-),	1067 (+), 1320 (-), 1404 (-), 706 (+),	1009 (+), 1410 (+), 151 (+), 526 (+), 840 (-),	366 (-),	365 (+), 58 (+),	1010 (-), 1034 (-), 1342 (-), 170 (+), 187 (-), 711 (-), 824 (-),	1178 (+), 1186 (+), 129 (+), 1446 (-), 195 (-), 206 (+), 418 (-), 437 (+), 469 (+), 496 (-), 551 (-), 601 (-), 656 (-), 706 (-), 863 (-),	1319 (+), 251 (+), 472 (+), 475 (+), 587 (+), 672 (+),
RAV1BAT	1470 (+),	-	-	-	-	-	1070 (+),	60 (-),	-	-	-	-	-	1088 (+), 474 (+)	-	716 (-),	1376 (+),	-	1386 (+),	569 (+),	-	860 (-),	-	-	386 (+), 682 (+)
RBCSCONSENSUS	168 (-),	-	160 (+),	-	-	-	800 (+), 870 (+),	-	-	-	-	-	124 (-), 331 (+), 700 (+), 953 (+)	-	-	-	507 (-),	-	-	-	943 (-),	-	123 (-),	-	450 (+),
REALPHALGLHCB21	-	629 (+),	-	1156 (-),	1010 (-), 640 (+),	385 (-),	-	889 (+),	146 (+),	1062 (+), 1106 (+),	1369 (-),	1121 (-), 1385 (-),	420 (-), 474 (-),	-	20 (-), 37 (-), 851 (-),	144 (-), 399 (-), 452 (-), 884 (+),	-	-	1436 (-), 358 (-), 883 (-),	-	1319 (-), 133 (-), 1406 (-),	-	-	-	-
REBETALGLHCB21	-	1088 (-), 248 (-)	-	-	-	218 (-),	-	256 (-),	-	1008 (+),	-	-	-	917 (-),	-	1487 (+),	1311 (+),	-	-	-	-	-	-	-	-
RGATAOS	-	-	-	-	-	-	-	-	-	-	-	-	-	-	-	-	-	-	-	-	-	-	-	-	-
RHERPATEXPA7	378 (+),	129 (+), 131 (-), 222 (-), 707 (+), 83 (+),	1411 (+), 88 (-),	1051 (-),	939 (+),	113 (+), 15 (+), 506 (-),	1166 (-), 862 (-),	1345 (+), 429 (+), 65 (+),	1298 (+), 878 (+),	723 (-), 786 (+), 871 (-), 929 (+),	691 (+),	52 (+), 549 (-),	1071 (-), 1127 (+), 227 (-), 768 (-),	373 (+), 375 (-),	362 (-), 408 (+), 410 (-), 919 (-), 994 (+),	921 (+), 976 (+),	700 (+), 969 (-),	612 (+),	1028 (+),	101 (-), 1178 (-), 141 (-), 501 (+), 611 (-), 615 (+), 668 (-),	1095 (+), 168 (-), 379 (+),	36 (-), 732 (+), 914 (+), 986 (+),	-	1078 (-), 1162 (+), 757 (-),	1144 (+), 1456 (+), 363 (-), 399 (+), 698 (-), 759 (+), 993 (-),
ROOTMOTIFTAPOX1	-	1051 (-), 446 (-), 56 (+),	1135 (+), 1140 (+), 1287 (-), 1288 (+), 808 (-), 834 (+),	103 (-), 1034 (-), 1035 (+), 104 (+), 1047 (-), 1296 (-), 1354 (-), 1456 (-), 201 (-), 247 (-), 367 (+),	1053 (-), 764 (-), 959 (-), 960 (+), 965 (+),	239 (+), 906 (-), 907 (+),	1128 (+), 1298 (-), 1299 (+), 189 (+), 226 (+), 260 (-), 261 (+), 444 (-), 472 (-), 683 (-), 684 (+), 836 (-), 837 (+), 89 (+),	1044 (-), 1110 (+), 730 (-), 731 (+),	1060 (-), 242 (+), 307 (-), 308 (+), 493 (-), 50 (-), 638 (-), 639 (+), 805 (-), 817 (+),	1011 (+), 1150 (-), 1163 (-), 1164 (+), 1177 (-), 1178 (+), 1195 (-), 228 (+),	307 (-), 308 (+), 6 (-), 663 (-), 664 (+), 67 (+), 7 (+), 706 (+), 736 (-), 755 (-),	1217 (-), 1218 (+),	1033 (+), 1034 (+), 120 (+), 825 (+),	1361 (+), 1419 (+), 912 (+),	1018 (+), 1104 (-), 1318 (-),	1144 (-), 1271 (-), 231 (-), 250 (+), 493 (-), 494 (+),	1212 (-), 1366 (-), 149 (+), 411 (-), 412 (+), 528 (-), 531 (+), 898 (-), 899 (+), 905 (+), 989 (-), 990 (+),	111 (-), 1316 (+), 593 (+), 654 (+), 676 (-), 677 (+), 729 (-), 730 (+),	56 (-), 57 (+), 595 (-), 596 (+), 759 (-),	1381 (-),	29 (+), 328 (-),	1033 (+), 1044 (-), 1047 (+), 1092 (-), 1100 (+), 1173 (-), 1179 (+),	1371 (-), 1372 (+), 427 (-), 622 (-), 623 (+), 809 (-), 810 (+), 944 (-),	158 (-), 159 (+), 378 (-), 392 (-), 445 (+), 506 (-), 507 (+), 557 (-), 578 (+), 611 (-), 612 (+), 683 (+), 834 (-), 835 (+),	-
RYREPEATBNNAPA	501 (+), 710 (-), 788 (-),	-	-	1054 (-), 652 (-), 654 (+),	617 (+),	331 (+), 333 (-), 359 (-), 928 (-), 989 (+),	1265 (-), 8 (+),	-	132 (-),	-	-	47 (-), 49 (+), 959 (-),	1012 (+),	1012 (-), 1014 (+),	696 (-), 991 (+),	1050 (+),	-	157 (+), 834 (-),	637 (+),	836 (-),	1092 (+),	39 (-), 971 (-), 973 (+), 975 (-),	-	792 (+),	-
RYREPEATGMGY2	709 (-),	-	-	-	617 (+),	331 (+), 332 (-),	-	-	-	-	-	-	-	-	-	-	-	-	-	-	-	973 (+), 974 (-),	-	792 (+),	-
RYREPEATLEGUMINBOX	709 (-),	-	-	1053 (-), 651 (-),	617 (+),	331 (+), 332 (-), 989 (+),	1264 (-),	-	-	-	-	49 (+),	1012 (+),	1014 (+),	695 (-), 991 (+),	-	-	-	-	-	1092 (+),	38 (-), 973 (+), 974 (-),	-	792 (+),	-
RYREPEATVFLEB4	-	-	-	-	-	331 (+), 331 (-),	-	-	-	-	-	-	-	-	-	-	-	-	-	-	-	973 (+), 973 (-),	-	-	-
S1FBOXSORPS1L21	-	-	-	1185 (+), 864 (+)	-	-	741 (-),	766 (+),	-	1452 (-),	-	1207 (-),	-	879 (+),	1237 (+),	-	126 (+), 722 (+)	-	620 (+),	-	1444 (-),	330 (-),	-	-	-
S1FSORPL21	-	-	-	-	-	-	-	-	-	-	-	-	-	-	-	-	722 (+)	-	-	-	-	-	-	-	-
S2FSORPL21	-	-	-	-	-	-	-	-	-	-	-	-	-	-	-	-	-	-	-	-	-	-	-	-	-
SBOXATRBCS	-	-	-	-	-	-	-	-	-	-	-	-	-	-	251 (-)	-	-	-	226 (-)	-	-	-	-	-	-
SEBFCONSSTPR10A	1035 (+),	-	-	1255 (+),	-	471 (-),	864 (-),	1176 (+), 143 (-),	-	-	-	1313 (+),	-	-	-	-	-	75 (-), 769 (-),	819 (-),	165 (+), 749 (-),	1323 (+), 546 (-),	1257 (-),	93 (+),	1240 (+),	-
SEF1MOTIF	-	-	-	1292 (-),	-	-	684 (+),	-	-	-	-	1218 (+),	-	1361 (+),	-	-	899 (+), 900 (+)	677 (+),	-	1377 (-),	-	1088 (-), 1120 (+)	-	159 (+),	-
SEF3MOTIFGM	164 (-),	1337 (-), 910 (+),	460 (-),	152 (-), 561 (-),	-	1216 (+),	-	-	474 (+), 829 (-),	1041 (+),	-	113 (+), 678 (-),	1432 (-), 880 (+),	1106 (-),	1480 (+),	1173 (+), 1438 (-), 416 (-), 468 (-), 473 (-), 799 (+), 833 (+), 838 (+), 867 (+),	453 (-), 628 (-),	407 (+), 712 (+),	-	-	579 (+),	-	1405 (-),	-	883 (+), 894 (+),
SEF4MOTIFGM7S	1084 (-), 349 (-), 701 (+),	625 (-), 662 (+), 941 (-),	1339 (-), 911 (-),	590 (+), 727 (+),	1083 (-),	743 (-), 766 (-),	1145 (-), 160 (+),	695 (+),	108 (-), 310 (+), 426 (-), 711 (-), 801 (-),	473 (+),	368 (+), 666 (+), 857 (-),	1166 (+),	313 (+), 325 (+), 914 (-),	-	1020 (+), 1267 (+),	1021 (+),	1230 (+), 1257 (+),	1234 (+), 1332 (+), 461 (-),	606 (-),	1426 (-), 940 (-),	226 (+), 443 (-), 45 (+), 513 (-), 575 (-), 865 (-), 896 (-), 937 (-)	1406 (-), 184 (+), 337 (+), 891 (+),	1425 (+), 284 (+), 345 (+), 982 (+),	1262 (-), 336 (+), 855 (-),	840 (-),
Si029660m	-	-	-	-	-	-	-	-	-	-	-	-	-	-	-	-	-	-	-	-	-	-	-	-	-
SITEIIATCYTC	1049 (+), 1234 (-), 1249 (-),	-	1473 (-), 274 (+), 276 (-), 392 (-), 454 (-),	644 (+),	1048 (-), 1137 (-), 1159 (-), 53 (+), 983 (+),	982 (+),	524 (-),	1214 (-), 1249 (+), 1265 (+), 1312 (-), 1321 (-),	-	1230 (+),	1095 (-),	137 (+), 162 (+), 184 (+), 198 (+), 200 (-),	-	1484 (+), 590 (+),	438 (+), 46 (+), 872 (-),	-	1381 (-),	-	1184 (+),	303 (-),	1086 (-), 119 (-), 551 (-), 659 (-), 701 (+),	511 (-),	1128 (+), 177 (+), 45 (+),	1182 (-),	1235 (-), 146 (+),
SITEIOSPCNA	-	-	-	-	-	-	1069 (-)	-	-	-	-	-	-	-	-	-	1375 (-)	-	-	-	-	-	-	-	-
SORLIP1AT	186 (-), 403 (-),	1244 (+), 265 (-), 281 (-), 324 (+),	172 (+), 376 (-), 41 (-),	-	123 (+), 1286 (+), 1332 (-), 15 (+), 424 (-), 72 (+),	233 (+), 316 (+), 377 (-),	1406 (+),	1240 (+), 1459 (+), 29 (+), 82 (+),	1468 (+), 418 (-), 539 (+), 933 (+),	664 (+), 739 (+), 773 (+), 808 (+), 880 (+),	-	1279 (-), 1356 (+), 446 (+), 673 (-), 769 (-), 783 (-), 894 (-), 924 (-),	631 (-),	129 (+), 141 (-), 42 (+), 472 (+), 50 (+), 514 (-), 741 (+), 985 (-),	151 (-), 159 (+), 211 (+), 566 (-), 661 (-), 916 (+),	973 (-),	1128 (-), 1390 (-), 1439 (+),	1036 (-), 66 (-),	1226 (+),	1080 (+), 325 (+), 335 (-), 434 (-), 473 (+), 513 (-), 578 (-), 584 (-), 60 (+), 66 (+),	1007 (+), 1119 (-), 1137 (+), 1242 (+), 607 (+), 965 (+),	1433 (-), 371 (+), 609 (+), 996 (-),	225 (+), 501 (+),	1075 (+), 1169 (+), 500 (+), 605 (+), 710 (+),	1043 (+), 235 (+), 5 (-), 607 (-), 680 (+), 80 (-), 969 (+), 997 (+),
SORLIP2AT	1050 (+), 1272 (-), 62 (-), 731 (+), 732 (-),	109 (+), 1359 (-), 1392 (-), 1476 (+), 1477 (-),	275 (+), 276 (-), 378 (-), 391 (+), 392 (-), 454 (-),	-	1048 (-), 1063 (+), 1137 (-), 1159 (-), 54 (+), 984 (+),	610 (-),	1384 (+), 524 (-),	1200 (+), 1230 (+), 1312 (-), 1321 (-),	-	619 (-),	1146 (+), 1465 (+), 899 (+),	163 (+), 199 (+), 200 (-), 829 (+),	152 (+),	131 (-), 287 (-), 591 (+),	312 (+), 313 (-), 339 (+), 340 (-), 439 (+), 872 (-),	-	1381 (-), 1392 (-), 386 (-),	-	-	1390 (-), 161 (-), 256 (-), 406 (+), 645 (+), 879 (-), 886 (-),	1085 (+), 1086 (-), 119 (-), 658 (+), 659 (-), 702 (+), 774 (+), 775 (-),	-	-	1028 (+), 1043 (-),	147 (+), 496 (-),
SORLIP5AT	-	-	1382 (+),	-	-	-	369 (+),	1180 (-), 969 (-)	-	-	-	-	-	1405 (-),	-	-	-	-	-	-	-	-	-	808 (+),	-
SORLREP3AT	-	-	-	-	-	-	-	-	337 (+)	-	-	-	-	-	-	-	-	-	-	-	-	-	-	-	-
SORLREP4AT	-	-	-	-	-	-	-	-	-	-	-	-	-	-	-	-	-	-	-	-	-	-	-	-	-
SP8BFIBSP8BIB	538 (-),	-	1069 (-),	-	-	-	-	-	-	-	-	-	-	-	-	280 (-),	-	-	-	1087 (-), 991 (-),	-	-	-	267 (+),	-
SPHCOREZMC1	-	-	-	-	-	-	-	-	-	-	-	-	-	-	-	-	-	-	-	-	-	-	-	-	-
SREATMSD	-	316 (-),	-	1171 (-),	-	-	266 (+),	574 (+),	-	1158 (-), 1187 (-),	-	-	-	916 (+),	-	-	854 (+),	853 (+),	-	-	1 (-), 181 (-),	111 (-), 543 (+), 573 (+)	1232 (-), 1328 (+), 577 (+)	371 (+), 963 (+),	-
SURE1STPAT21	-	973 (-),	-	-	-	-	-	-	-	-	-	-	-	-	-	-	-	-	-	-	-	-	-	581 (-), 686 (-)	-
SURE2STPAT21	-	-	-	-	-	-	-	-	409 (+)	-	-	-	-	-	-	-	-	-	-	-	-	-	-	-	-
SURECOREATSULTR11	763 (-),	1268 (-), 1462 (-), 229 (-), 439 (-), 725 (+), 932 (+), 955 (+), 967 (+),	1373 (+), 1434 (-),	1257 (-),	1262 (+),	-	-	1178 (-), 223 (+),	1234 (-),	164 (+), 307 (-),	1338 (+), 336 (-),	1315 (-),	1289 (-), 1305 (+), 1354 (-),	-	932 (+),	1040 (+), 69 (-), 78 (-),	568 (-), 768 (+), 790 (+), 848 (+),	75 (+), 769 (+),	342 (+), 819 (+),	167 (-),	1325 (-), 619 (+),	1257 (+), 356 (+),	115 (+), 149 (+),	1064 (+),	115 (+), 120 (+), 1347 (-), 169 (-),
SV40COREENHAN	392 (+), 744 (-)	-	-	374 (+),	-	1025 (+),	-	-	-	1045 (-),	-	-	-	-	750 (+), 760 (-)	-	-	481 (-),	-	-	-	-	-	-	240 (-),
TAAAGSTKST1	296 (-), 493 (-), 737 (-),	-	1177 (-), 874 (-),	137 (-), 399 (-),	-	1013 (-), 1337 (+), 1373 (+), 1434 (-), 352 (-), 920 (+),	1088 (-), 1091 (+), 1246 (-), 241 (-), 45 (+), 697 (-),	-	1036 (-), 626 (+),	121 (+), 1237 (-), 289 (+), 485 (+),	1005 (-), 166 (+), 176 (-), 33 (+), 549 (+), 829 (+),	1193 (-),	38 (+), 513 (+),	1221 (+),	-	959 (+),	1119 (-), 446 (-), 57 (-), 695 (-), 772 (+), 803 (-),	352 (-),	406 (-), 651 (+), 746 (+), 802 (+), 852 (-),	-	273 (+), 92 (+),	1069 (+), 1272 (-), 1290 (+), 155 (+), 342 (+), 483 (+), 541 (-), 869 (-)	1320 (+), 1473 (+), 912 (+),	1250 (-), 260 (-), 905 (-),	194 (+), 91 (+),
TATABOX2	-	-	-	1179 (-), 1292 (+), 509 (-),	-	-	-	636 (+),	-	-	-	-	-	1363 (-),	-	-	901 (-), 992 (-), 995 (+),	469 (-),	-	-	-	-	-	-	-
TATABOX3	-	-	508 (-),	-	-	1299 (+),	1295 (-),	-	635 (-),	-	-	1234 (+),	-	-	-	228 (-),	-	-	-	-	298 (-),	-	-	-	-
TATABOX4	-	-	-	70 (+),	-	-	-	-	1062 (+),	-	-	1195 (-),	-	-	-	-	-	1238 (-),	-	788 (+),	17 (+), 198 (+)	-	-	174 (+), 380 (+)	-
TATABOX5	298 (+),	1009 (+), 1013 (+), 1017 (+), 1047 (-), 666 (+), 739 (-), 811 (+)	1008 (-),	1132 (+), 1482 (+), 385 (-),	968 (+),	1015 (+), 1334 (-), 1401 (-), 451 (+), 497 (+), 547 (+), 769 (-)	-	671 (-), 786 (+),	1080 (+), 44 (-), 938 (+),	1144 (-), 1167 (+),	115 (+), 182 (+), 620 (-), 981 (-),	1065 (+), 1252 (+), 995 (+),	1436 (+), 424 (+),	-	1252 (-),	1129 (-), 1136 (-), 1249 (-), 781 (-),	1152 (+), 1159 (+), 1184 (+), 448 (+), 730 (+),	252 (+), 386 (+), 390 (+), 681 (+),	1234 (-),	-	324 (-), 510 (-),	1040 (-), 1183 (+), 1251 (+), 29 (-), 456 (-),	105 (+), 140 (+), 282 (+), 516 (+), 645 (-), 907 (+),	27 (-), 347 (+),	282 (-),
TATABOXOSPAL	-	-	-	-	-	202 (+), 548 (+), 803 (-)	-	727 (-),	-	1208 (-),	-	1219 (+),	-	-	1250 (-),	-	731 (+),	-	-	1378 (-),	322 (-),	1022 (-), 1089 (-), 1184 (-)	908 (+),	-	-
TATAPVTRNALEU	-	-	-	-	-	-	-	-	-	-	-	1194 (+),	-	-	-	-	-	1237 (+),	-	-	-	-	-	380 (-),	-
TATCCACHVAL21	-	-	-	-	-	-	-	575 (+)	-	-	-	-	844 (-)	-	-	-	-	-	-	-	-	-	1329 (+)	-	-
TATCCAOSAMY	926 (-),	-	-	110 (-), 1170 (-), 337 (-),	-	-	267 (+),	575 (+),	-	1157 (-),	-	-	215 (+), 845 (-),	1079 (+), 1358 (-),	-	749 (+),	855 (+),	854 (+),	211 (-),	-	180 (-),	1081 (+), 110 (-), 574 (+),	1231 (-), 1329 (+), 578 (+),	372 (+),	-
TATCCAYMOTIFOSRAMY	925 (-),	-	-	109 (-),	-	-	-	575 (+),	-	1156 (-),	-	-	215 (+), 844 (-),	1079 (+),	-	749 (+),	855 (+),	854 (+),	210 (-),	-	-	1081 (+),	1329 (+),	-	-
TBOXATGAPB	1032 (+),	690 (-),	1032 (-), 569 (-),	-	1254 (-),	1417 (-), 1444 (-),	528 (-), 874 (-),	348 (+),	1172 (-), 478 (-),	-	239 (+), 342 (-), 450 (-),	-	-	-	-	-	-	1300 (+), 982 (-),	1209 (+),	1224 (-),	-	-	1101 (+), 600 (+), 738 (-),	-	-
TE2F2NTPCNA	-	-	-	-	-	-	-	-	-	-	-	99 (+)	-	-	-	-	-	-	-	-	-	-	-	-	1403 (+)
TELOBOXATEEF1AA1	-	-	-	-	-	182 (-)	-	-	-	-	-	682 (-)	-	-	-	-	-	-	-	-	-	-	-	-	1426 (-)
TGACGTVMAMY	1368 (+), 750 (-),	225 (+),	-	-	-	-	-	-	-	-	-	-	1069 (+),	-	766 (+),	-	702 (-),	1041 (+), 1043 (-), 438 (-),	-	-	1097 (-),	52 (+), 54 (-),	-	-	-
T/GBOXATPIN2	-	423 (-),	1167 (+),	-	940 (-),	16 (-), 725 (-)	-	66 (-),	-	-	-	-	336 (+), 767 (+)	-	-	-	-	486 (-),	-	-	-	1328 (+), 991 (+)	-	-	-
TGTCACACMCUCUMISIN	-	-	-	-	-	470 (-)	-	-	-	-	-	-	-	-	-	-	-	-	90 (-)	-	545 (-)	-	-	-	-
TRANSINITDICOTS	157 (-)	-	-	-	292 (-)	-	-	-	-	-	-	-	-	-	244 (-)	-	-	-	-	-	-	669 (-)	-	-	-
TRANSINITMONOCOTS	157 (-),	866 (-),	449 (-), 781 (-)	-	150 (-), 292 (-)	-	-	-	1442 (-),	751 (-),	-	911 (-),	-	-	244 (-),	1225 (-),	-	-	-	-	-	669 (-),	-	-	1328 (-),
UP1ATMSD	1046 (-),	-	276 (+),	-	1048 (+),	-	-	-	-	-	-	195 (-),	-	-	-	-	-	-	-	-	698 (-),	-	-	-	-
UP2ATMSD	-	1380 (-)	1466 (+)	-	-	183 (-)	-	-	-	1490 (+)	-	683 (-)	1416 (-)	-	-	1391 (+)	-	-	-	-	1400 (-)	-	-	-	1427 (-)
UPRMOTIFIAT	748 (+)	-	-	-	-	-	-	-	-	-	-	-	-	-	-	-	-	-	-	-	-	-	-	-	-
UPRMOTIFIIAT	734 (+)	-	375 (-)	-	-	-	-	-	-	-	-	-	-	-	759 (+)	508 (-)	-	-	1375 (-)	-	-	-	-	-	-
VSF1PVGRP18	-	-	-	-	156 (+)	-	-	-	-	-	-	-	-	-	-	-	-	-	-	-	-	-	-	-	-
WBBOXPCWRKY1	-	-	-	-	1142 (-), 575 (+),	-	-	-	1169 (-),	-	24 (-), 38 (+),	190 (-),	547 (-),	-	-	828 (-),	812 (-),	-	-	-	-	-	602 (+), 735 (-), 752 (+)	-	-
WBOXATNPR1	1100 (-), 1126 (+), 323 (+), 330 (+), 752 (-),	1197 (-), 216 (+), 301 (-),	13 (-), 1334 (-), 616 (+),	1127 (-), 447 (-),	1143 (-), 458 (-), 576 (+), 684 (+),	-	1051 (-), 206 (+), 284 (-), 904 (+),	1194 (-), 551 (-),	1170 (-), 253 (-), 302 (-), 642 (+),	246 (+), 948 (+),	1127 (+), 126 (+), 141 (-), 25 (-), 311 (+), 39 (+),	191 (-), 33 (+),	1027 (+), 187 (+), 548 (-), 751 (-),	1146 (+), 771 (+),	1191 (-), 1345 (+), 1419 (-), 765 (+),	829 (-),	813 (-),	1045 (-), 1261 (-), 1415 (+), 937 (-),	1322 (+), 139 (+),	972 (-),	317 (-),	1131 (+), 56 (-),	1182 (-), 168 (-), 603 (+), 736 (-), 753 (+),	127 (-),	585 (-), 835 (+),
WBOXHVISO1	1030 (+), 1099 (-), 756 (-),	300 (-), 705 (-),	1333 (-),	1126 (-), 446 (-), 661 (+), 951 (-),	1142 (-), 577 (+),	-	283 (-), 554 (+), 952 (+),	1277 (+), 1352 (+), 550 (-),	1169 (-), 1286 (+), 328 (-),	86 (+),	1058 (-), 1128 (+), 24 (-), 312 (+),	124 (+), 190 (-),	1065 (+), 547 (-),	1142 (+), 1147 (+), 1347 (+), 944 (+),	-	993 (-), 998 (+),	692 (+), 812 (-), 820 (+),	1009 (+), 1117 (+), 1416 (+), 936 (-),	1323 (+), 232 (+),	1026 (-), 754 (-),	316 (-),	1132 (+), 1224 (-),	1181 (-), 754 (+),	-	263 (+), 42 (-),
WBOXNTCHN48	-	1485 (+),	-	-	-	1144 (+),	1410 (+), 951 (+),	1276 (+),	-	592 (+),	562 (-),	-	180 (+),	1141 (+), 1346 (+), 943 (+)	813 (-),	-	-	1008 (+),	1487 (+),	1026 (-), 754 (-),	1215 (-),	-	-	987 (+),	958 (+),
WBOXNTERF3	1030 (+), 1099 (-), 1127 (+), 1228 (+), 756 (-),	1196 (-), 1486 (+), 300 (-), 705 (-),	1333 (-),	1126 (-), 446 (-), 661 (+), 951 (-),	1142 (-), 231 (-), 302 (+), 457 (-), 577 (+),	111 (-), 1145 (+), 13 (-), 647 (+), 79 (+),	1050 (-), 1411 (+), 283 (-), 554 (+), 952 (+),	1277 (+), 1352 (+), 550 (-),	1169 (-), 1286 (+), 328 (-),	1257 (+), 247 (+), 593 (+), 86 (+),	1058 (-), 1128 (+), 127 (+), 24 (-), 312 (+), 40 (+), 480 (+), 562 (-),	124 (+), 190 (-),	1065 (+), 181 (+), 547 (-), 750 (-),	1142 (+), 1147 (+), 1347 (+), 944 (+),	716 (-), 813 (-),	828 (-), 993 (-), 998 (+),	692 (+), 812 (-), 820 (+),	1009 (+), 1117 (+), 1416 (+), 936 (-),	1323 (+), 1328 (-), 1488 (+), 232 (+), 324 (+), 633 (+), 66 (+),	1026 (-), 754 (-),	1215 (-), 316 (-),	1132 (+), 1224 (-),	1181 (-), 604 (+), 735 (-), 754 (+),	126 (-), 842 (+), 988 (+),	263 (+), 42 (-), 836 (+), 959 (+),
WRKY71OS	1030 (+), 1037 (-), 1100 (-), 1127 (+), 1228 (+), 1284 (+), 1368 (+), 324 (+), 331 (+), 446 (-), 752 (-), 757 (-), 817 (+),	1197 (-), 145 (+), 1486 (+), 217 (+), 225 (+), 301 (-), 706 (-),	13 (-), 1334 (-), 188 (+), 617 (+),	1123 (-), 1127 (-), 170 (+), 447 (-), 661 (+), 709 (+), 722 (-), 952 (-),	1133 (-), 1143 (-), 116 (+), 1435 (+), 1478 (+), 232 (-), 302 (+), 377 (-), 458 (-), 577 (+), 685 (+), 88 (+),	112 (-), 1145 (+), 14 (-), 402 (-), 472 (+), 482 (-), 490 (+), 647 (+), 79 (+),	1051 (-), 1269 (+), 1411 (+), 207 (+), 284 (-), 554 (+), 865 (+), 905 (+), 952 (+),	1194 (-), 1277 (+), 1352 (+), 144 (+), 239 (+), 376 (+), 544 (-), 551 (-),	1140 (-), 1170 (-), 1281 (-), 1286 (+), 1476 (-), 253 (-), 297 (-), 302 (-), 329 (-), 643 (+), 69 (-),	1257 (+), 247 (+), 593 (+), 86 (+), 949 (+),	1059 (-), 1128 (+), 127 (+), 141 (-), 25 (-), 312 (+), 40 (+), 480 (+), 518 (-), 563 (-),	1052 (-), 124 (+), 191 (-), 34 (+),	1028 (+), 1065 (+), 1069 (+), 1264 (-), 181 (+), 188 (+), 548 (-), 683 (-), 751 (-),	1086 (-), 1142 (+), 1147 (+), 1347 (+), 462 (-), 702 (-), 772 (+), 944 (+),	1191 (-), 1346 (+), 1419 (-), 396 (+), 700 (+), 717 (-), 766 (+), 814 (-),	217 (-), 713 (+), 829 (-), 994 (-), 998 (+),	692 (+), 704 (-), 739 (-), 813 (-), 820 (+), 863 (+),	1009 (+), 1041 (+), 1045 (-), 1094 (+), 1117 (+), 1184 (+), 1261 (-), 1416 (+), 205 (+), 232 (+), 440 (-), 569 (+), 871 (-), 937 (-),	1323 (+), 1329 (-), 1364 (+), 1370 (+), 140 (+), 1488 (+), 232 (+), 324 (+), 633 (+), 66 (+), 92 (+),	1027 (-), 250 (+), 750 (+), 755 (-), 844 (-), 852 (-), 901 (-), 972 (-),	1099 (-), 1216 (-), 317 (-), 547 (+), 597 (+),	1132 (+), 1198 (-), 1225 (-), 1456 (+), 194 (+), 52 (+), 56 (-), 864 (+),	1182 (-), 168 (-), 482 (+), 604 (+), 736 (-), 754 (+), 95 (-),	1242 (-), 127 (-), 842 (+), 988 (+),	106 (+), 263 (+), 39 (-), 43 (-), 585 (-), 701 (+), 836 (+), 959 (+),
WUSATAg	-	534 (+), 759 (-)	-	-	-	-	1017 (+), 828 (-)	-	-	-	-	-	-	-	-	-	-	-	-	-	-	-	-	975 (-),	304 (-),
XYLAT	-	-	-	790 (-)	-	536 (-)	-	-	117 (-)	-	-	-	-	1467 (-)	-	-	-	902 (-)	-	-	-	-	-	133 (+)	1250 (+)
ZDNAFORMINGATCAB1	-	-	-	-	-	-	-	-	-	-	-	-	-	-	-	-	-	-	-	-	-	-	-	-	-

PYRIMIDINEBOXOSRAMY	658 (+),	125 (-),	566 (+), 818 (-),	344 (-), 425 (+), 436 (+), 450 (+)	308 (-), 555 (-),	993 (+),	1384 (-), 851 (-),	1351 (+), 1478 (-), 370 (+), 757 (+)	-	1162 (-),	1354 (+), 273 (-),	1228 (-),	-	145 (+),	321 (-), 409 (-),	1011 (+), 85 (-), 851 (+), 896 (-),	-	514 (+), 672 (-),	-	158 (-),	1366 (-), 808 (+),	575 (-),	1445 (+),	1073 (-), 322 (+), 338 (+),	-	-
QARBNEXTA	-	-	-	-	-	27 (-),	-	699 (-),	-	-	-	-	-	-	-	-	-	-	965 (-),	-	-	-	-	-	-	-
QELEMENTZMZM13	-	-	-	1464 (-),	-	-	1481 (+),	1481 (+),	-	566 (-), 680 (-)	119 (+),	755 (-),	361 (+),	-	13 (+),	-	-	-	394 (+),	-	-	578 (+),	852 (-),	165 (+),	625 (-),	-
RAV1AAT	1148 (-), 356 (-), 585 (-), 618 (-),	118 (-), 215 (-), 241 (-), 762 (+),	215 (-), 372 (+), 392 (-), 485 (+), 957 (+),	1262 (-), 1359 (-), 368 (+),	549 (+), 624 (-), 648 (+), 651 (+), 768 (+), 879 (+),	866 (-), 898 (+),	1313 (-), 46 (-), 541 (+), 632 (-),	50 (+), 533 (+), 853 (-),	1238 (+), 1472 (+), 168 (+), 269 (+), 376 (-), 571 (+), 919 (-),	1119 (+), 1460 (-), 262 (-), 469 (-), 619 (+), 757 (+), 889 (-),	1129 (-), 225 (-), 25 (-), 384 (-), 403 (+),	1042 (-), 243 (+), 436 (-), 534 (+), 948 (-),	840 (+),	1183 (+), 119 (+), 1272 (-), 1319 (+), 1415 (+), 16 (-), 439 (+), 442 (+), 470 (-), 486 (+), 491 (-),	1105 (-), 1154 (+), 351 (+), 500 (+),	1080 (-), 1277 (+), 1370 (+), 1385 (-), 1491 (-),	1014 (-), 1310 (+), 1360 (+), 53 (+),	1067 (-), 140 (-), 15 (-), 575 (+), 590 (-), 713 (+), 770 (-), 999 (-),	1037 (-), 1359 (+), 334 (-), 784 (-), 91 (-),	1243 (+), 416 (+), 52 (-), 817 (-), 912 (+), 918 (+),	105 (-), 1321 (+), 1414 (-), 155 (+), 183 (+), 536 (-), 851 (-), 921 (-),	375 (-), 571 (+),	1011 (+), 1223 (-), 1409 (+), 312 (+), 671 (-),	1431 (+),	1073 (-), 265 (-), 505 (+), 552 (+),	1077 (-), 1243 (+), 800 (-),
RAV1BAT	-	-	-	-	-	1013 (+),	1363 (-),	-	-	760 (+),	-	112 (-), 2 (-), 786 (-)	421 (+), 893 (-)	1407 (+), 1430 (-)	1144 (-),	889 (+),	1119 (+), 246 (+)	-	-	242 (-),	-	411 (+),	1109 (-), 1359 (+)	1414 (+),	-	-
RBCSCONSENSUS	-	-	-	-	457 (-),	-	-	300 (-),	-	-	-	573 (+),	-	886 (-),	-	-	27 (+),	1382 (+), 485 (+),	-	175 (+),	713 (-),	-	-	-	-	-
REALPHALGLHCB21	-	1044 (+), 1376 (+), 334 (-), 610 (+), 614 (+), 776 (-)	-	1124 (+),	645 (+),	-	15 (-),	-	89 (-),	-	1076 (+),	-	1076 (-), 1301 (-), 555 (+),	551 (+), 695 (-), 98 (-),	-	-	1095 (-), 32 (+),	-	1464 (+), 513 (-), 592 (+), 940 (+),	1006 (+), 1025 (+), 1066 (+), 861 (+),	-	1 (-), 568 (+),	-	-	1428 (+),	1413 (+),
REBETALGLHCB21	-	-	1307 (+),	758 (+),	-	-	-	-	-	-	-	1480 (-), 605 (+)	-	-	-	-	1442 (+),	-	117 (-),	-	651 (-),	-	-	-	-	-
RGATAOS	-	-	-	-	-	-	-	-	-	-	-	-	-	-	-	-	-	-	-	-	-	-	-	-	129 (-)	-
RHERPATEXPA7	207 (+), 674 (-), 84 (-),	40 (+),	1469 (-), 318 (-), 509 (-), 560 (-),	-	939 (+),	388 (+), 390 (-), 623 (-), 666 (+),	37 (+),	1157 (-), 1405 (+),	1068 (-), 1342 (-), 793 (-),	118 (-), 372 (-),	863 (+),	1171 (-),	989 (+),	127 (+),	892 (-),	712 (-),	743 (+),	534 (+), 652 (-),	1057 (-),	-	1017 (+), 1048 (-), 1093 (-), 505 (-), 584 (+), 899 (+),	935 (-),	1050 (+), 168 (-),	1161 (-), 281 (+), 707 (-), 822 (+),	1104 (-),	1427 (+),
ROOTMOTIFTAPOX1	1164 (+), 1237 (+), 1305 (+), 1361 (+), 336 (-), 749 (+),	1049 (-), 1178 (+), 1253 (+), 557 (-),	123 (+), 238 (-),	585 (-), 586 (+), 620 (+), 685 (+),	1368 (-), 1388 (-), 1461 (-), 1462 (+), 319 (-), 593 (+),	200 (-), 201 (+), 264 (+), 594 (-), 595 (+), 726 (+),	555 (-), 622 (+),	111 (+), 1263 (+), 1282 (-), 1314 (-), 1315 (+), 503 (+), 514 (-), 667 (-), 782 (-), 814 (-), 815 (+), 876 (-), 896 (+),	713 (-), 842 (-),	149 (+), 534 (+), 79 (+),	1300 (+), 1475 (-), 1476 (+), 1481 (+), 158 (+), 423 (+), 498 (-), 683 (+), 923 (-), 955 (-),	1208 (+), 1242 (-), 1243 (+),	492 (+),	1130 (-), 1131 (+), 352 (-),	228 (-), 236 (+), 241 (+), 264 (+), 334 (+), 417 (-), 737 (-),	1073 (+), 1125 (+), 1426 (-),	751 (-), 752 (+),	734 (+),	1099 (+), 1142 (+), 148 (-), 151 (+), 319 (+), 430 (-), 431 (+),	1118 (+), 383 (-), 922 (-),	44 (-), 45 (+), 501 (-), 530 (+),	1186 (-), 680 (-), 681 (+), 708 (-), 918 (-), 997 (-), 998 (+),	390 (+), 586 (-), 587 (+), 621 (-), 622 (+), 678 (-), 679 (+), 817 (-), 818 (+), 847 (+), 936 (-), 937 (+), 958 (-), 959 (+),	1092 (-),	127 (-), 396 (-), 712 (-),	1068 (-), 1201 (-), 1202 (+), 352 (-), 372 (-), 393 (-), 488 (+),
RYREPEATBNNAPA	134 (+),	145 (+), 267 (+),	96 (+),	488 (-),	280 (-), 293 (-),	246 (-),	-	979 (-),	178 (+), 550 (+), 552 (-),	225 (+), 788 (+),	-	897 (+), 899 (-),	794 (+),	-	-	1039 (-),	-	1063 (-),	-	252 (+), 604 (+),	408 (-),	-	-	960 (+),	1121 (-), 976 (-),	114 (-), 12 (-), 1291 (+), 1293 (-), 14 (+), 16 (-), 18 (+), 553 (-), 555 (+)
RYREPEATGMGY2	-	-	-	-	-	245 (-),	-	978 (-),	550 (+), 551 (-),	-	-	897 (+), 898 (-),	-	-	-	-	-	-	-	-	407 (-),	-	-	-	1120 (-),	113 (-), 1291 (+), 1292 (-), 14 (+), 15 (-), 553 (-)
RYREPEATLEGUMINBOX	-	-	-	-	-	245 (-),	-	978 (-),	550 (+), 551 (-),	225 (+),	-	897 (+), 898 (-),	794 (+),	-	-	1038 (-),	-	-	-	-	407 (-),	-	-	-	1120 (-), 975 (-),	113 (-), 1291 (+), 1292 (-), 14 (+), 15 (-), 552 (-), 555 (+)
RYREPEATVFLEB4	-	-	-	-	-	-	-	-	550 (+), 550 (-),	-	-	897 (+), 897 (-),	-	-	-	-	-	-	-	-	-	-	-	-	-	1291 (+), 1291 (-), 14 (+), 14 (-),
S1FBOXSORPS1L21	-	1158 (+),	-	-	435 (-),	399 (+),	457 (-),	188 (+), 748 (-)	391 (+), 708 (-)	-	-	-	-	-	-	-	-	125 (-), 1258 (-)	-	680 (+),	-	-	-	-	-	50 (+),
S1FSORPL21	-	-	-	-	-	-	-	-	-	-	-	-	-	-	-	-	-	-	-	-	-	-	-	-	-	50 (+)
S2FSORPL21	-	-	-	-	1341 (+)	-	-	-	-	-	-	-	-	-	-	-	-	-	-	-	-	-	-	-	-	-
SBOXATRBCS	-	-	-	-	-	-	-	-	831 (+)	-	-	-	-	-	-	-	-	-	-	-	915 (-)	-	-	-	-	-
SEBFCONSSTPR10A	1365 (+),	919 (+), 98 (-),	-	-	-	308 (+),	817 (+),	-	1094 (-), 1216 (+), 1394 (-)	710 (+),	29 (-),	1173 (-),	-	-	1487 (+),	1446 (-),	-	1307 (+),	1373 (+),	-	-	-	1402 (+),	-	1016 (-),	586 (-),
SEF1MOTIF	-	-	-	620 (+),	-	-	-	-	-	-	-	-	-	-	264 (+),	-	-	734 (+),	-	-	-	-	-	-	-	-
SEF3MOTIFGM	435 (+),	-	968 (+),	-	-	1176 (+), 437 (+),	105 (-),	364 (+),	-	1069 (+),	-	-	142 (+), 695 (+),	186 (-), 191 (-), 196 (-),	-	-	-	608 (-),	-	-	657 (+),	404 (-), 6 (-),	-	1338 (+),	1218 (-), 1435 (+), 227 (-),	1098 (+), 1452 (-), 437 (+),
SEF4MOTIFGM7S	958 (+),	1236 (-), 540 (-),	262 (+),	329 (+), 578 (-),	148 (-),	-	1057 (-), 1081 (+), 1447 (+), 606 (+),	1295 (-), 304 (+), 887 (+),	1354 (-),	1455 (+), 554 (+),	106 (-), 1448 (+), 262 (+),	640 (+), 687 (+), 927 (-),	1249 (-), 1304 (+), 180 (-),	421 (+),	1390 (+), 483 (-),	1307 (+), 1374 (+),	1186 (+), 23 (-),	421 (-), 800 (+),	221 (+), 48 (+),	151 (-),	-	-	335 (+), 834 (+), 867 (-),	-	1404 (-),	1262 (-), 368 (-),
Si029660m	-	-	-	-	-	-	-	-	-	-	-	-	-	-	-	-	-	-	-	-	-	-	-	-	-	-
SITEIIATCYTC	-	139 (+),	711 (+), 823 (-),	1019 (+), 1069 (-), 1089 (-),	1084 (+), 219 (-), 559 (-), 850 (-),	-	1295 (+), 1316 (+), 138 (+), 188 (+), 657 (-), 712 (+), 747 (-), 773 (+), 798 (-),	24 (+),	1061 (+), 1124 (-), 1289 (+), 1294 (+), 1304 (-),	884 (+),	-	1203 (-), 1237 (-), 1264 (-), 1275 (-), 48 (-),	-	-	-	-	1292 (-), 1298 (-), 1356 (-), 140 (+), 632 (-),	1438 (-), 1470 (-),	-	1360 (+), 1415 (+), 1444 (-),	379 (+), 615 (+), 689 (-), 737 (+), 754 (-),	-	1352 (-), 1378 (-), 220 (+),	1046 (-), 1477 (-), 641 (+),	731 (+), 806 (+), 832 (+), 864 (+),	1080 (+), 1093 (-), 72 (+),
SITEIOSPCNA	-	-	-	-	-	1012 (-)	-	-	-	-	-	-	-	-	-	-	-	-	-	-	-	-	-	-	-	-
SORLIP1AT	480 (-), 6 (-), 912 (-),	1390 (-), 229 (-), 232 (+), 37 (-), 605 (-),	1369 (-), 171 (+), 382 (+), 695 (-),	1056 (+), 1275 (+), 503 (-), 518 (-),	1195 (+), 485 (+), 913 (-),	1456 (+), 99 (+),	636 (-), 745 (-), 779 (-),	1491 (-),	1234 (+), 415 (+), 589 (+), 676 (+), 970 (+),	1098 (-), 1487 (-),	1108 (+),	1166 (+), 1318 (+), 387 (-), 414 (-), 426 (-), 491 (-), 674 (-),	1322 (+), 1464 (+),	773 (-),	-	1030 (-), 1273 (+),	1398 (+), 1427 (+), 215 (+), 600 (+),	1432 (+), 1476 (+), 692 (-),	1486 (+), 973 (-),	1105 (+), 312 (+), 545 (+), 887 (+),	1296 (+), 1486 (+), 968 (+),	1122 (-), 379 (+), 948 (+),	1074 (+), 113 (-),	38 (-), 423 (+), 44 (-), 65 (-), 751 (-),	1364 (+), 1400 (+), 341 (-), 535 (-), 835 (+), 867 (+), 957 (+),	1083 (+), 1487 (+), 620 (-), 832 (+), 916 (-),
SORLIP2AT	709 (+),	312 (-),	1063 (+), 1183 (+), 1184 (+), 1199 (-), 1381 (-), 811 (-), 822 (+), 823 (-),	1020 (+), 1069 (-), 1089 (-), 975 (-), 993 (+),	1035 (+), 1193 (+), 559 (-), 760 (-),	1008 (-), 1190 (-), 1482 (+), 1483 (-),	1317 (+), 139 (+), 153 (-), 174 (+), 189 (+), 521 (-), 682 (+), 713 (+), 747 (-), 798 (-),	335 (-),	1041 (-), 1056 (-), 1062 (+), 1124 (-), 1330 (+), 1349 (+),	1174 (+),	1272 (-),	1237 (-), 299 (+),	-	-	846 (+),	1482 (+), 349 (+), 350 (-), 455 (-),	1292 (-), 1298 (-), 1425 (+), 149 (+), 226 (+), 227 (-), 356 (+),	1277 (+), 1438 (-), 511 (+),	975 (-),	1361 (+), 1444 (-),	380 (+), 381 (-), 616 (+), 635 (+), 738 (+), 754 (-),	-	1352 (-), 1378 (-), 1389 (+),	1132 (-), 1484 (-), 642 (+),	732 (+), 807 (+), 833 (+), 865 (+),	1081 (+), 1375 (+), 1445 (-), 73 (+), 883 (+),
SORLIP5AT	251 (+),	1429 (+),	-	-	-	-	-	-	-	-	-	-	-	785 (-),	-	-	-	851 (-),	-	-	-	-	-	-	-	-
SORLREP3AT	610 (+)	-	-	-	-	-	-	-	-	-	-	-	-	-	-	-	-	-	-	-	-	-	-	-	-	-
SORLREP4AT	-	-	-	194 (+)	1236 (-)	608 (-)	-	-	-	-	-	-	-	-	-	-	-	-	-	-	-	-	-	-	-	-
SP8BFIBSP8BIB	-	-	-	-	490 (-),	-	291 (-),	-	-	-	811 (+), 914 (+),	419 (-),	-	1152 (+),	-	-	-	-	382 (+), 453 (+), 768 (+)	-	-	-	-	1151 (+),	-	-
SPHCOREZMC1	-	-	-	-	-	-	-	-	-	-	-	898 (-)	-	-	-	-	-	-	-	-	-	-	-	-	-	1289 (+)
SREATMSD	-	-	629 (+),	544 (+), 650 (+),	-	905 (+),	-	182 (+), 308 (+),	-	35 (-),	1321 (+), 602 (-), 645 (+)	1479 (+),	-	1126 (-),	413 (-),	902 (-),	1443 (-),	1100 (+), 408 (-),	-	-	-	-	86 (+),	209 (-), 360 (+),	358 (+),	1 (+),
SURE1STPAT21	-	733 (-),	-	-	-	-	-	-	-	-	-	-	900 (-),	-	-	-	-	-	-	-	-	-	-	-	-	-
SURE2STPAT21	-	-	-	-	-	-	-	-	-	-	-	-	-	-	-	-	-	-	-	-	-	-	-	-	-	-
SURECOREATSULTR11	266 (+), 787 (-),	1307 (-), 1401 (+), 248 (+), 921 (-), 98 (+),	-	135 (-), 39 (+), 788 (+),	30 (-), 348 (+), 545 (-),	298 (+), 670 (+), 765 (-),	70 (+), 813 (-), 819 (-), 830 (+),	-	1094 (+), 1218 (-), 1447 (-), 1488 (-), 228 (+), 505 (+),	2 (+), 510 (+),	1405 (+), 534 (+),	1337 (+), 84 (-), 961 (+), 99 (-),	325 (+), 707 (-),	1400 (-), 1475 (+),	134 (+), 1489 (-), 800 (+),	188 (-),	1075 (+), 1259 (+), 1411 (+), 923 (-),	1309 (-), 133 (+), 1356 (-), 823 (-), 890 (+),	841 (-),	218 (-), 342 (-), 449 (-),	133 (+), 324 (+),	743 (+), 851 (-),	-	1289 (-), 398 (-), 431 (-), 926 (+),	1016 (+), 366 (-),	1230 (-), 586 (+),
SV40COREENHAN	984 (+),	-	526 (-),	1101 (-), 948 (+)	1440 (+),	321 (-),	1046 (+), 1117 (+)	-	-	-	-	-	-	-	883 (-),	-	-	378 (+),	-	-	234 (+),	-	-	-	402 (+),	-
TAAAGSTKST1	942 (-),	1197 (+),	653 (+), 899 (+), 923 (-),	389 (+), 648 (-),	1352 (+), 432 (-),	455 (-), 562 (-),	1407 (+), 1437 (+), 340 (+), 549 (+), 66 (+),	778 (-),	-	425 (+), 602 (+),	1115 (+), 1288 (+), 182 (+), 608 (+),	338 (-), 709 (+),	155 (+), 202 (-),	37 (-), 379 (+), 682 (-), 828 (+),	1229 (-), 176 (-), 273 (-), 727 (-), 931 (+),	779 (-),	-	362 (+), 493 (-),	1123 (-), 1406 (+), 663 (+), 84 (-), 878 (+),	119 (-), 578 (-), 671 (+),	176 (-), 287 (+), 3 (+),	1238 (+), 1366 (-), 221 (-), 477 (+), 802 (-),	1126 (-), 1131 (-), 1158 (+), 248 (-), 39 (+), 436 (+), 772 (-), 859 (-),	101 (+), 78 (+), 946 (-),	840 (+), 872 (+),	496 (+),
TATABOX2	-	-	231 (+),	-	1347 (-),	154 (+),	-	435 (-),	292 (-), 64 (+),	-	1296 (-), 772 (+),	265 (+),	-	-	266 (-), 428 (+),	-	-	-	-	-	771 (+),	710 (+),	981 (+),	-	-	1182 (-), 1185 (+), 251 (+), 482 (-)
TATABOX3	-	-	-	-	-	-	-	816 (+), 817 (-)	-	-	-	-	-	315 (+),	492 (+), 493 (-)	-	1007 (+),	-	427 (-),	-	-	915 (-),	-	-	-	-
TATABOX4	-	-	229 (+),	-	144 (+),	-	566 (-),	437 (-), 438 (+)	-	-	-	-	-	-	-	-	-	-	1066 (-),	-	-	873 (+),	774 (-), 775 (+)	-	-	484 (-),
TATABOX5	1178 (+), 1183 (+), 1269 (+), 1384 (-),	1057 (-), 1068 (-), 1089 (+), 1144 (+), 780 (-), 972 (+),	234 (-),	581 (-), 624 (+),	-	-	1192 (+), 1392 (-), 1404 (-), 722 (-),	222 (+), 488 (+), 506 (+), 595 (-), 869 (-), 941 (-),	453 (+),	1112 (-),	1218 (+), 1314 (+), 695 (+), 840 (+), 935 (-),	1082 (-), 356 (+), 628 (+), 638 (+), 726 (-),	1171 (-), 1225 (+), 398 (-),	152 (+), 913 (-),	1056 (-), 705 (+), 829 (+), 876 (+),	-	990 (-),	1038 (+), 1220 (-), 471 (+), 521 (+),	671 (+),	204 (-), 350 (-),	-	474 (-), 583 (-),	413 (-), 918 (-), 943 (-),	-	299 (-),	239 (+), 505 (-), 87 (-),
TATABOXOSPAL	-	1145 (+),	-	-	-	-	-	-	-	-	-	-	-	-	-	-	-	1218 (-), 735 (+),	-	-	-	-	916 (-),	-	-	-
TATAPVTRNALEU	-	-	229 (-),	-	144 (-),	-	565 (+),	436 (+), 438 (-)	-	-	-	-	-	-	-	-	-	-	-	-	-	-	773 (+), 775 (-)	-	-	483 (+),
TATCCACHVAL21	522 (-)	-	-	-	-	-	-	-	-	-	646 (+)	-	-	-	-	-	-	-	-	-	905 (-)	-	-	-	1389 (+)	-
TATCCAOSAMY	523 (-),	1051 (+),	-	545 (+),	-	906 (+),	-	183 (+),	-	-	1322 (+), 155 (-), 601 (-), 646 (+), 680 (-),	-	-	-	-	-	-	372 (+),	345 (+), 58 (-),	707 (-),	906 (-),	-	87 (+),	208 (-), 361 (+),	1389 (+), 359 (+),	-
TATCCAYMOTIFOSRAMY	522 (-),	-	-	545 (+),	-	-	-	183 (+),	-	-	1322 (+), 154 (-), 646 (+)	-	-	-	-	-	-	-	57 (-),	706 (-),	905 (-),	-	87 (+),	207 (-),	1389 (+), 359 (+),	-
TBOXATGAPB	1291 (+),	-	-	-	564 (+),	540 (-),	-	390 (-), 764 (+),	1258 (+), 508 (+),	353 (-),	1010 (-), 1044 (+),	1070 (+), 1452 (-), 846 (+), 954 (+)	567 (-),	-	-	1034 (-), 1099 (-),	-	-	-	-	-	131 (-),	-	-	-	101 (-), 1236 (-), 1344 (-), 545 (+)
TE2F2NTPCNA	-	-	-	-	-	-	-	-	-	1256 (+)	-	-	-	-	-	-	-	-	-	-	-	-	-	-	-	-
TELOBOXATEEF1AA1	-	-	1284 (-)	-	-	-	-	-	-	105 (+)	-	-	-	-	-	-	-	-	-	-	-	-	-	-	-	-
TGACGTVMAMY	-	1083 (-), 661 (+), 950 (+), 952 (-)	-	398 (+),	-	-	483 (-),	-	791 (+),	529 (-),	-	-	549 (+),	-	-	197 (+),	1040 (+), 1042 (-),	582 (-),	66 (+),	811 (+),	-	-	1052 (-),	-	1102 (+),	1464 (+), 1466 (-),
T/GBOXATPIN2	-	234 (-),	-	-	-	28 (-), 622 (+)	-	700 (-),	852 (-),	-	-	-	-	-	891 (+),	-	-	-	966 (-),	-	-	934 (+),	-	282 (-),	-	-
TGTCACACMCUCUMISIN	-	-	-	-	-	584 (+)	-	-	-	711 (+)	28 (-)	-	770 (-)	-	-	1445 (-)	-	-	-	-	-	-	-	-	-	-
TRANSINITDICOTS	-	-	120 (-)	-	63 (-)	396 (-)	-	451 (-)	-	-	420 (-)	-	-	-	-	1070 (-)	-	-	-	677 (-)	-	-	-	-	-	-
TRANSINITMONOCOTS	-	-	120 (-),	-	63 (-),	396 (-),	-	451 (-), 615 (-)	-	726 (-),	420 (-),	-	-	-	-	1070 (-),	-	-	-	677 (-),	637 (-),	-	-	658 (-),	-	-
UP1ATMSD	-	-	823 (+),	-	-	-	-	-	-	-	-	1237 (+),	-	-	-	-	1298 (+),	-	-	-	612 (-), 734 (-)	-	-	-	-	-
UP2ATMSD	-	-	1285 (-)	-	-	1371 (-)	-	-	-	105 (+)	-	-	-	-	-	-	-	-	-	-	-	-	-	-	-	-
UPRMOTIFIAT	-	1081 (+)	-	-	-	-	-	-	-	-	-	-	-	-	-	-	-	-	66 (-)	-	-	-	-	-	-	-
UPRMOTIFIIAT	-	-	1071 (-)	-	-	-	-	-	1137 (-)	-	-	-	-	-	-	-	-	-	-	-	1020 (-)	-	-	-	-	-
VSF1PVGRP18	-	-	-	-	-	-	-	-	-	-	-	-	-	-	-	-	-	-	-	-	-	-	-	-	-	819 (+)
WBBOXPCWRKY1	-	-	-	-	68 (-),	-	-	-	-	350 (-),	-	-	-	-	92 (+),	-	-	-	-	-	-	579 (-),	350 (+), 850 (+),	166 (-),	-	568 (+), 83 (-),
WBOXATNPR1	1411 (+), 977 (-),	954 (-),	1152 (-), 192 (-), 477 (+),	101 (-),	69 (-), 840 (+),	731 (-),	194 (+), 539 (-),	1237 (-), 1483 (-), 526 (+), 87 (+), 952 (+), 971 (+)	251 (+), 982 (+),	351 (-), 384 (+), 664 (-), 935 (+),	1014 (-), 121 (-), 623 (-), 68 (-), 686 (+), 948 (+),	754 (+), 849 (+), 986 (-),	223 (-), 348 (-), 355 (+), 519 (+), 548 (+),	548 (-),	1080 (-), 15 (-), 757 (+), 93 (+),	171 (+), 989 (-),	1044 (-), 889 (-),	1396 (-), 288 (-), 528 (-),	1118 (-),	768 (+),	1425 (+), 307 (-), 332 (-),	580 (-), 618 (-), 677 (-),	1041 (+), 1264 (+), 274 (-), 351 (+), 675 (-), 851 (+),	167 (-), 973 (+),	1173 (-), 1188 (+), 236 (-),	1205 (+), 1342 (-), 399 (-), 569 (+), 84 (-),
WBOXHVISO1	1078 (+), 1402 (+),	163 (+), 256 (+),	-	-	124 (+), 195 (-), 211 (-), 633 (+),	-	-	1160 (+), 1236 (-), 527 (+), 953 (+),	1379 (+), 341 (+), 612 (-), 939 (+),	1403 (-), 350 (-),	1013 (-), 687 (+),	1115 (-), 985 (-),	570 (-), 599 (+),	1026 (+), 1439 (+), 1469 (+), 957 (-),	1245 (-), 653 (+), 94 (+),	1178 (+),	1159 (-), 1165 (+), 1238 (+), 593 (-), 623 (+)	1395 (-), 532 (-), 995 (+),	1165 (+), 1452 (-), 249 (+), 883 (+),	364 (+),	73 (+), 88 (-),	-	352 (+), 548 (+),	684 (-), 817 (-), 974 (+), 978 (+),	1172 (-),	1206 (+), 398 (-), 570 (+), 591 (-), 954 (+),
WBOXNTCHN48	-	255 (+),	-	1463 (+),	123 (+), 211 (-),	-	1482 (-), 243 (+),	-	340 (+), 612 (-),	1403 (-), 565 (+),	-	-	-	1468 (+),	1245 (-), 652 (+),	-	1159 (-), 1483 (+),	-	395 (-),	363 (+),	149 (+), 480 (-),	-	-	817 (-), 977 (+),	1426 (-),	953 (+),
WBOXNTERF3	1078 (+), 130 (+), 1402 (+), 1412 (+),	163 (+), 256 (+),	305 (+), 478 (+),	1464 (+),	1045 (-), 124 (+), 195 (-), 211 (-), 259 (-), 633 (+), 68 (-), 685 (+),	894 (+),	1482 (-), 244 (+), 698 (+),	1160 (+), 1236 (-), 1482 (-), 527 (+), 538 (+), 953 (+),	1155 (-), 1379 (+), 322 (+), 341 (+), 612 (-), 939 (+),	1403 (-), 350 (-), 566 (+), 680 (+),	1013 (-), 120 (-), 1398 (-), 687 (+),	1115 (-), 755 (+), 985 (-),	362 (-), 570 (-), 599 (+),	1026 (+), 1439 (+), 1469 (+), 957 (-),	1245 (-), 14 (-), 653 (+), 94 (+),	1138 (-), 1178 (+), 172 (+),	1159 (-), 1165 (+), 1238 (+), 1484 (+), 593 (-), 623 (+),	1395 (-), 194 (+), 532 (-), 995 (+),	1165 (+), 1452 (-), 249 (+), 395 (-), 883 (+),	364 (+),	1327 (-), 1426 (+), 150 (+), 331 (-), 480 (-), 666 (-), 73 (+), 88 (-),	579 (-),	1042 (+), 146 (-), 273 (-), 352 (+), 548 (+), 736 (+), 852 (+),	166 (-), 684 (-), 817 (-), 974 (+), 978 (+),	1172 (-), 1426 (-), 513 (+), 625 (+),	1206 (+), 398 (-), 570 (+), 591 (-), 611 (+), 707 (+), 83 (-), 954 (+),
WRKY71OS	1078 (+), 1218 (-), 130 (+), 1367 (-), 1402 (+), 1412 (+), 16 (+), 232 (-), 567 (+), 977 (-),	1085 (-), 1105 (-), 1205 (-), 163 (+), 256 (+), 319 (-), 661 (+), 950 (+), 954 (-),	1152 (-), 1263 (+), 192 (-), 219 (-), 305 (+), 478 (+), 66 (-), 834 (+),	101 (-), 1140 (+), 1464 (+), 222 (+), 270 (+), 276 (+), 398 (+), 853 (+), 859 (+), 888 (+),	1014 (+), 1046 (-), 124 (+), 133 (+), 196 (-), 212 (-), 260 (-), 265 (-), 600 (+), 633 (+), 685 (+), 69 (-), 841 (+),	133 (-), 310 (-), 432 (-), 585 (-), 731 (-), 894 (+),	1483 (-), 195 (+), 244 (+), 485 (-), 539 (-), 698 (+), 908 (+),	1160 (+), 1237 (-), 1360 (+), 1483 (-), 527 (+), 538 (+), 620 (-), 88 (+), 953 (+), 972 (+),	11 (-), 1156 (-), 1379 (+), 1395 (+), 252 (+), 322 (+), 341 (+), 382 (+), 568 (+), 613 (-), 690 (-), 791 (+), 939 (+), 953 (+), 983 (+),	1142 (-), 1404 (-), 351 (-), 385 (+), 531 (-), 566 (+), 664 (-), 680 (+), 712 (-), 936 (+), 959 (-), 977 (+),	1014 (-), 121 (-), 1243 (-), 1399 (-), 219 (+), 30 (+), 571 (+), 623 (-), 673 (+), 68 (-), 687 (+), 738 (+), 949 (+),	1116 (-), 1174 (+), 602 (+), 755 (+), 772 (+), 790 (+), 850 (+), 986 (-),	1356 (-), 223 (-), 348 (-), 356 (+), 363 (-), 520 (+), 549 (+), 571 (-), 599 (+), 772 (+),	1026 (+), 1439 (+), 1453 (+), 1469 (+), 417 (+), 548 (-), 608 (-), 958 (-),	1080 (-), 1246 (-), 15 (-), 185 (+), 653 (+), 758 (+), 94 (+),	1121 (+), 1139 (-), 116 (-), 1178 (+), 1447 (+), 1463 (-), 172 (+), 197 (+), 285 (-), 950 (-), 989 (-),	1040 (+), 1044 (-), 1124 (-), 1160 (-), 1165 (+), 1233 (-), 1238 (+), 1250 (-), 1484 (+), 594 (-), 623 (+), 641 (-), 692 (-), 742 (-), 889 (-),	1071 (-), 1396 (-), 194 (+), 288 (-), 528 (-), 533 (-), 548 (+), 584 (+), 96 (-), 995 (+),	1118 (-), 1165 (+), 1290 (+), 1375 (-), 1453 (-), 249 (+), 396 (-), 66 (+), 883 (+),	1323 (+), 1379 (-), 364 (+), 769 (+), 811 (+),	1328 (-), 1380 (+), 1401 (+), 1421 (-), 1426 (+), 150 (+), 307 (-), 332 (-), 481 (-), 667 (-), 73 (+), 801 (+), 89 (-),	298 (+), 514 (-), 556 (-), 580 (-), 618 (-), 677 (-), 938 (+),	1042 (+), 1054 (-), 1265 (+), 1294 (-), 1369 (+), 1404 (-), 147 (-), 274 (-), 352 (+), 548 (+), 675 (-), 736 (+), 852 (+),	1265 (+), 167 (-), 518 (-), 685 (-), 818 (-), 974 (+), 978 (+),	1102 (+), 1169 (-), 1173 (-), 1189 (+), 137 (-), 1427 (-), 236 (-), 451 (-), 513 (+), 625 (+),	1206 (+), 1342 (-), 1393 (-), 1464 (+), 1468 (-), 399 (-), 524 (+), 570 (+), 592 (-), 611 (+), 707 (+), 84 (-), 954 (+),
WUSATAg	-	713 (+),	-	-	-	-	-	-	-	-	-	-	-	-	794 (+),	-	-	-	307 (-), 425 (-)	-	-	-	-	-	-	-
XYLAT	1019 (-)	-	-	-	-	-	1428 (-)	718 (+)	-	-	-	-	-	-	-	-	-	-	-	-	-	-	-	-	-	-
ZDNAFORMINGATCAB1	605 (+)	177 (+)	-	-	-	-	-	-	-	-	-	-	-	-	-	-	-	-	-	-	-	-	-	-	-	-

- not present

Table S5. Distribution of methylation percentase for TSS and Gene region of *SiSET* genes

NIPGR ID	Chrom	Gene Start	Gene End	IC04_CHG	IC04_CHH	IC04_CpG	TSS Start	TSS End	IC04_CHG	IC04_CHH	IC04_CpG
SiSET01	1	9262162	9263521	28	55	65	9261961	9262161	0	0	0
SiSET02	1	27431267	27443199	6	22	388	27431066	27431266	10	14	32
SiSET03	1	30584825	30593856	7	44	109	30584624	30584824	0	0	0
SiSET05	1	31530485	31535057	1	1	38	31530284	31530484	0	0	0
SiSET06	1	33041260	33043602	4	6	92	33041059	33041259	0	0	1
SiSET07	1	36022715	36033504	36	60	3468	36022514	36022714	0	0	3
SiSET08	1	36742251	36746753	9	24	72	36742050	36742250	2	1	0
SiSET09	1	37260864	37263996	9	14	84	37260663	37260863	21	30	79
SiSET10	2	7396424	7403493	13	20	177	7396223	7396423	0	0	1
SiSET11	2	11909548	11915017	24	40	403	11909347	11909547	8	20	42
SiSET12	2	19909313	19923580	151	125	2008	19909112	19909312	0	0	0
SiSET13	2	22841147	22843798	48	58	944	22840946	22841146	10	26	25
SiSET14	2	25532388	25541393	10	6	237	25532187	25532387	0	0	0
SiSET15	2	28151597	28156452	12	38	302	28151396	28151596	0	0	0
SiSET16	2	39722445	39733003	62	79	2731	39722244	39722444	0	0	1
SiSET17	2	40099240	40102634	71	51	1773	40099039	40099239	0	1	1
SiSET18	2	46973422	46978473	10	23	126	46973221	46973421	0	0	0
SiSET19	3	5778304	5783126	0	11	43	5778103	5778303	0	0	0
SiSET20	3	8565460	8570027	3	11	98	8565259	8565459	0	0	0
SiSET21	3	14937095	14942247	7	23	218	14936894	14937094	0	0	0
SiSET22	3	22555774	22562876	4	12	66	22555573	22555773	10	18	32
SiSET23	3	49605515	49612901	13	44	1121	49605314	49605514	0	0	6
SiSET24	4	1082524	1090272	5	14	88	1082323	1082523	0	0	0
SiSET25	4	4508310	4517397	1	8	235	4508109	4508309	0	0	1
SiSET26	4	7728850	7732975	2	4	120	7728649	7728849	0	0	0
SiSET27	5	13713202	13720765	43	80	281	13713001	13713201	0	0	0
SiSET28	5	30969211	30976908	29	52	463	30969010	30969210	0	0	2
SiSET29	5	38137796	38139908	25	20	69	38137595	38137795	0	0	0
SiSET30	5	39622569	39627037	5	10	173	39622368	39622568	29	65	79
SiSET31	5	42910037	42912248	10	42	87	42909836	42910036	0	0	0
SiSET32	5	45431833	45439471	3	17	159	45431632	45431832	0	1	0
SiSET33	5	46948448	46951251	29	52	197	46948247	46948447	7	8	7
SiSET36	6	.	.	15	46	1530	3266415	3266615	0	0	0
SiSET37	6	9104290	9107203	1	4	31	9104089	9104289	0	0	0
SiSET38	6	24675305	24677638	108	158	171	24675104	24675304	2	8	15
SiSET39	6	24819022	24822852	39	49	123	24818821	24819021	16	25	74
SiSET41	6	35909936	35911855	1	1	101	35909735	35909935	0	0	4
SiSET42	6	35945072	35948105	0	4	109	35944871	35945071	0	2	1
SiSET44	7	20122709	20128474	1	2	88	20122508	20122708	8	12	15
SiSET45	7	27469561	27473548	301	59	390	27469360	27469560	8	0	26
SiSET46	7	30627990	30631961	3	2	84	30627789	30627989	0	0	0
SiSET47	7	34631824	34633773	0	2	38	34631623	34631823	0	0	0
SiSET48	8	271043	276325	2	10	25	270842	271042	14	167	39
SiSET49	9	7774932	7779473	2	12	61	7774731	7774931	14	20	45
SiSET50	9	14179183	14184721	0	2	66	14178982	14179182	0	14	0
SiSET51	9	21436977	21439968	0	3	34	21436776	21436976	0	2	0
SiSET52	9	48379173	48386403	6	6	155	48378972	48379172	2	5	1
SiSET53	9	55283355	55285198	18	26	92	55283154	55283354	1	0	0

Table S6. List of primers for 21 *SiSET* genes used in quantitative real time-PCR expression profiling.

Name	FORWARD PRIMERS (5′- 3′)	REVERSE PRIMERS (5′- 3′)
**SiSET01**	GGTGGTGTCCAAGAAGAAGAGG	CTGGTAGGTGAGGTGGTTGCTG
**SiSET02**	TCCATAGGCAGTGATGTTGCTC	CCTCCTTCAGATTCAGGGGTTC
**SiSET06**	CAAGTTCCGCTTCCAGGTCT	CCTTTTCTGTGCCTCTTCTTCCTC
**SiSET09**	GGCCGTCCAGTTTAGATTGCAG	CCTAAGCGGTGGATTTCTGTGG
**SiSET14**	GTGGGATGCTGTTCTCTTGTTG	GGTCTGTGCTGGAATGGTTTG
**SiSET15**	GGTCTCACCCATACGCACATTTC	CTTCTAACGGCAACCCCTCCTC
**SiSET19**	GGTGATTCTGTTCGGTTGGTG	AGCCTGGAGTTTGTTTGTGGTC
**SiSET22**	CCGTCAACAGAAAACTGCAC	AACATGCCCACGGATAACTC
**SiSET23**	AACCAAAATCTGCTGGGCTCTC	CATCCTCTGGGCTACTGCTAAGG
**SiSET25**	AGCCTGATAGAGCCCCTGC	GTTGCCCGTCATCCTTTCCTC
**SiSET26**	TCACTCCCTCGCAAACTCCTC	CCTGCCCCTCTTCCTCTTACTG
**SiSET27**	GGGTCTCCTGTGGTCTATCTGG	GGTATCGTGCTTCCCTCAAGTC
**SiSET28**	GTGTGATGCTATTTGGCTGGTG	TGCTTAGTTCTGTCTGCCCTTG
**SiSET30**	AAGGACCGCATCATTTCACC	AGTTGGCATCACCAGGCAGAC
**SiSET36**	TTCAGGGATAACACGCCAAGTC	ACGAAATGGGCTACGGTCTC
**SiSET37**	TCAAGGAGCAGGAAGAGGAATG	TCAAGAGCAGTATCCCCCACAC
**SiSET39**	TGCGACCTACACCATTCCGATAC	TCCCCTCCATTCTTCACAGCAC
**SiSET42**	ATGGGAGTGGCTGACTTTGGAC	CCTATGGCTGGTTTGTGGTGA
**SiSET45**	ACGGAAGTGATGTCGGGTC	CAACTGATGGCAAACGGAAC
**SiSET49**	GATTCTTCCTTCGCATGAGC	TACAATCCAGTCCCAAGAGGTC
**SiSET53**	GCTCGGGAAGAATGGTGTT	TTGGGGTGGCAAGAATGG
